# Prevalence of *pfk13* and *pfmdr1* polymorphisms in Bounkiling, Southern Senegal

**DOI:** 10.1371/journal.pone.0249357

**Published:** 2021-03-26

**Authors:** Ambroise Ahouidi, Rafael Oliveira, Lis Lobo, Cyrille Diedhiou, Souleymane Mboup, Fatima Nogueira

**Affiliations:** 1 Laboratory of Bacteriology and Virology, Hospital Aristide Le Dantec, Dakar, Senegal; 2 Institute for Health Research, Epidemiological Surveillance and Training (IRESSEF), Dakar, Senegal; 3 Global Health and Tropical Medicine, GHTM, Instituto de Higiene e Medicina Tropical, IHMT, Universidade NOVA de Lisboa, UNL, Lisboa, Portugal; Instituto Rene Rachou, BRAZIL

## Abstract

**Background:**

Delayed *Plasmodium falciparum* parasite clearance has been associated with Single Nucleotide Polymorphisms (SNPs) in the kelch protein propeller domain (coded by *pfk13* gene). SNPs in the *Plasmodium falciparum* multidrug resistance gene 1 (*pfmdr1)* are associated with multi-drug resistance including the combination artemether-lumefantrine. To our knowledge, this is the first work providing information on the prevalence of k13-propeller and *pfmdr1* mutations from Sédhiou, a region in the south of Senegal.

**Methods:**

147 dried blood spots on filter papers were collected from symptomatic patients attending a hospital located in Bounkiling City, Sédhiou Region, Southern Senegal. All samples were collected between 2015–2017 during the malaria transmission season. Specific regions of the gene *pfk13* and *pfmdr1* were analyzed using PCR amplification and Sanger sequencing.

**Results:**

The majority of parasites (92.9%) harboured the *pfk13* wild type sequence and 6 samples harboured synonymous changes. Regarding *pfmdr1*, wild-type alleles represented the majority except at codon 184. Overall, prevalence of 86Y was 11.9%, 184F was 56.3% and 1246Y was 1.5%. The mutant allele 184F decreased from 73.7% in 2015 to 40.7% in 2017. The prevalence of haplotype NFD decreased from 71.4% in 2015 to 20.8% in 2017.

**Conclusions:**

This study provides the first description of *pfk13* and *pfmdr1* genes variations in Bounkiling, a city in the Sédhiou Region of Senegal, contributing to closing the gap of information on anti-malaria drug resistance molecular markers in southern Senegal.

## Introduction

Malaria caused by *P*. *falciparum* remains a public health problem with the majority of cases and deaths occurring in sub-Saharan Africa [1]. In Senegal, incremental interventions have significantly reduced malaria morbidity and mortality rates [2,3]. Nonetheless, in Senegal half a million people in the country suffered from malaria during 2018 [1].

Prevalence of malaria in Senegal varies across regions, with extremely low prevalence in northern (0.1%) and western (0.2%) regions, and higher prevalence (up to 5.9%) in the southern region [2,3]. Following World Health Organization (WHO) recommendations, in 2006, the Senegalese health authorities changed the first line antimalarial drug to fixed dose artemisinin combination therapies (ACTs); artemether+lumefantrine (AL) and artesunate+amodiaquine (ASAQ), as first and second lines of treatment for uncomplicated *P*. *falciparum* malaria [4]. Artemisinin resistance, as well as resistance to other antimalarial partner drugs are present in 5 countries of the Greater Mekong subregion (in Southeast Asia) [5–7] and although it has not yet been irrefutably documented in Africa [1], some reports have emerged lately [8–10].

Genome-wide analysis of artemisinin resistance in *P*. *falciparum* has demonstrated that mutations in the propeller domain of the gene encoding the Kelch 13 (K13) protein (*pfk13*) are associated with resistant *in vitro* and *in vivo* phenotypes in Southeast Asia [6,11,12]. Nine nonsynonymous mutations have been validated (F446**I**, N458**Y**, M476**I**, Y493**H**, R5397**T**, I543**T**, P553**L**, R561**H** and C580**Y**) in the *Pfk13* gene as molecular markers, alongside with eleven candidate mutations [13]. In sub-Saharan Africa, increasing frequencies of nonsynonymous mutations on the *pfk13* gene have been reported throughout the continent [14–16] including in Senegal [17,18] and the neighbouring The Gambia [19,20], Guinea-Bissau [21] and Mali [22].

The *P*. *falciparum multidrug resistance transporter 1* (*pfmdr1*) gene encodes an ABC transporter protein located in the digestive vacuole of the parasite [23]. *Pfmdr1* polymorphisms at codons N86Y, Y184F, S1034C, N1042D, and D1246Y have been associated with drug resistance to several antimalarials [24–26]. Drug pressure (*in vivo*) due to ACT partner drugs has resulted in directional selection of *pfmdr1* variants: 86Y, Y184 and 1246Y for amodiaquine (AMQ) and N86, 184F and D1246 for AL [24–27] The same tendency is also observed *in vitro* and *ex vivo* susceptibility assays [28,29]. Interestingly, allelic replacement of *pfmdr1* 86Y was able to increase parasite susceptibility to dihydroartemisinin (DHA) *in vitro* [30].

**Fig 1:**
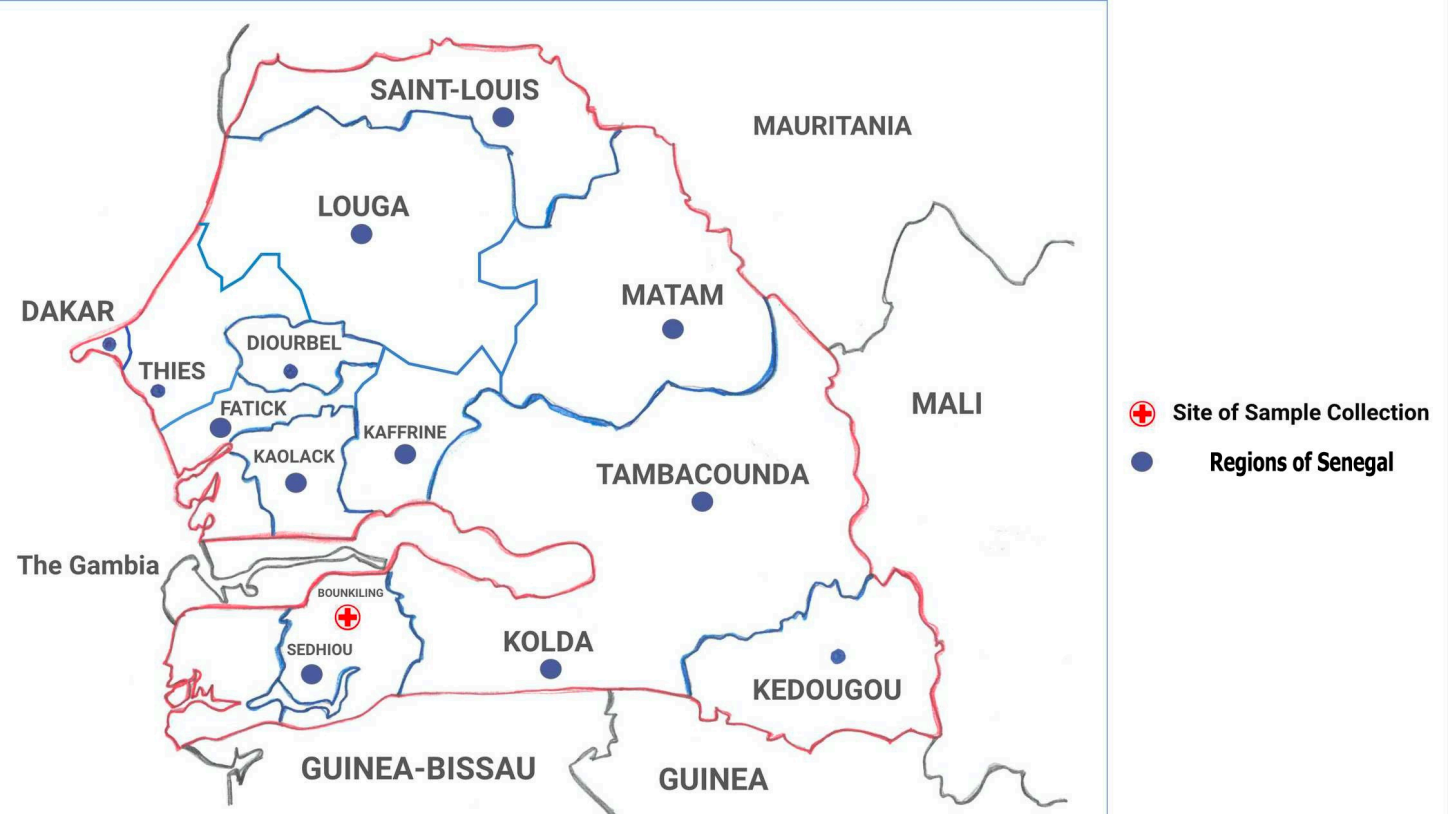
Map of Senegal showing the different regions and the sample collection site.

*Ex vivo* susceptibilities to ACT partner drugs, lumefantrine (LUM) and AMQ, have been decreasing since 2008 in Senegal and neighboring The Gambia and Mali [19,20,31]. In The Gambia, some isolates have shown declining sensitivity to DHA in in vitro assays [19]. Despite sustained drug pressure (accessible through public and private health facilities) and a high burden of malaria, few studies on *P*. *falciparum* molecular markers of drug resistance surveillance are available from southern regions of Senegal [32]. Most of the studies of molecular marker surveillance in Senegal have been performed in Dakar and Thiés [17,31,33–41] (Fig 1). Our study provides information on *pfk13* and *pfmdr1* from Bounkiling, a city located in the Sédhiou region, in the South of Senegal (Fig 1).

Sédhiou has year-round malaria transmission that peaks during the rainy season (June through October) with sustained but lower transmission the rest of the year. This region lies between two malaria endemic countries: The Gambia (66 cases/1000 people at risk) and Guinea-Bissau (123 cases/1000 people at risk) [1].

## Materials and methods

### Sample collection

In this study, we collected 147 samples (blood spots on Whatman filter paper) from *P*. *falciparum* infected patients with uncomplicated malaria, attending the health center in Bounkiling, Senegal. After laboratory confirmation of malaria infection by microscopy or rapid diagnostic test, patients were asked to participate in the study. Written informed consent was obtained from all patients before sample collection. The study was reviewed and approved by the Ethical Committee of the Ministry of Health of Senegal (082 MSAS/DPRS/CNERS). Samples were collected during the peak malaria transmission period (October to December) in 2015, 2016 and 2017.

### Genotyping of *pfmdr1* and *pfk13* genes

*P*. *falciparum* mutations associated with resistance to ACT components were typed using PCR amplification and Sanger sequencing. We evaluated specific regions of the genes *pfk13* (codon 436 to 706) and *pfmdr1* (codons 86, 184 and 1246). DNA from 147 blood spots was extracted using Chelex method [42] and DNA was stored at -20°C.

For *pfk13* one fragment containing the main polymorphisms associated with delayed clearance in Southeast Asia was amplified by nested PCR as described elsewhere [43] with modifications. Briefly, specific primers were developed for this purpose (forward—3’ GAAAGAAGCAGAATTTTATGG5’; reverse—3’ GCTTGGCCCATCTTTATTAGTTCCC 5’, obtaining a fragment of 856bp. A semi-nested PCR was performed in some samples using the inner forward primer 3’ GTGTAGAATATTTAAATTCG 5’ obtaining a fragment of 788bp.

For *pfmdr1*, specific primers were designed for the amplification of the fragment of 452bp containing codons 86 and 184 (forward—3’ GTATGTGCTGTATTATCAGGAGGA 5’; reverse—3’ TTAATTTATGTTTGTGGTGTCATATG 5’) and for the amplification of the fragment of 508bp containing codon 1246 (forward—3’ CTACAGCAATCGTTGGAGAA 5’ reverse—3’ GAGAATAGCTATAGCTAGAGC 5’). PCR conditions 94°C 2 min; [94°C 1 min, 56°C 1 min, 72°C 1 min] 10X; [94°C 1 min, 50°C 1 min, 72°C 1 min] 30X; 72°C 3 min. An aliquot of the PCR products was analyzed by electrophoresis on a 2% agarose gel stained with GreenSafe Premium (Nzytech, Portugal) to confirm single band amplification. All PCR products were then purified using SureClean Plus (Bioline) before Sanger sequencing at Eurofins Genomics (GATC services, Germany).

### Data analysis

Sequences were analyzed using Multalin software (http://multalin.toulouse.inra.fr/multalin/multalin.html; free online) using *P*. *falciparum*-3D7 strain as wild type genotype.

The prevalence of a particular mutant allele was calculated as the proportion of the specific mutant samples among the total number of samples successfully analyzed for this mutation. All graphical representations and calculations were performed with GraphPad Prism PRISM 8 for MAC (V8.4.2).

## Results

### Sample characteristics

Among the 147 samples analyzed, twenty-one of them tested negative for *P*. *falciparum* by PCR (6/39 in 2015, 6/65 in 2016 and 9/43 in 2017), hence removed from further analysis. Of the remaining 126 blood samples, 7 had no slide available, so they were not considered for the parasitemia evaluation that varied between 0.02–9.33% with a median value of 0.67%. Of the 126 subjects, 72 (57.14%) were men and 54 (42.86%) were women, and age varied between 3–70 years, with a median of 18 years. The majority 46.83% (n = 59) were adults (over 20 years), 40.48% (n = 51) were adolescents and 12.69% (n = 16) were children between the age of 3 and 10 years.

### Molecular markers of anti-malarial drug resistance

All 126 samples (from day 0 before treatment), were successfully sequenced for *pfk13*, 120 were wild type (identical to the reference, 3D7 strain) and 6 carried synonymous mutations (Table 1). One of the synonymous mutations, C469C was previously described in Senegal [17]. None of the *pfk13* variants associated with artemisinin resistance in Southeast Asia [13] were observed in our study (Table 1).

**Table 1:** Mutations in *pfk13* and *pfmdr1 genes*.

Year (n)	*pfk13*	*pfmdr1*						
		Allele prevalence (%)						Haplotype (%)
	Codon (*)	N86	86Y	Y184	184F	D1246	1246Y	
**2015 (33)**	F583F (2)	90.9	9.1	26.3	73.7	100	0	NFD (71.4)
								NYD (14.3)
								YFD (14.3)
**2016 (59)**	F491F (1)	90.2	9.8	42.0	58.0	100	0	NFD (54.5)
								NYD (40.9)
								YFD (4.5)
**2017 (34)**	G545G (1)	82.1	17.9	59.3	40.7	96.0	4.0	NFD (20.8)
	C469C (1)							NYD (62.5)
	A627A (1)							YFD (16.7)
**overall**	6*/126	88.1	11.9	43.8	56.3	98.5	1.5	

*number of samples with synonymous mutation.

**Fig 2:**
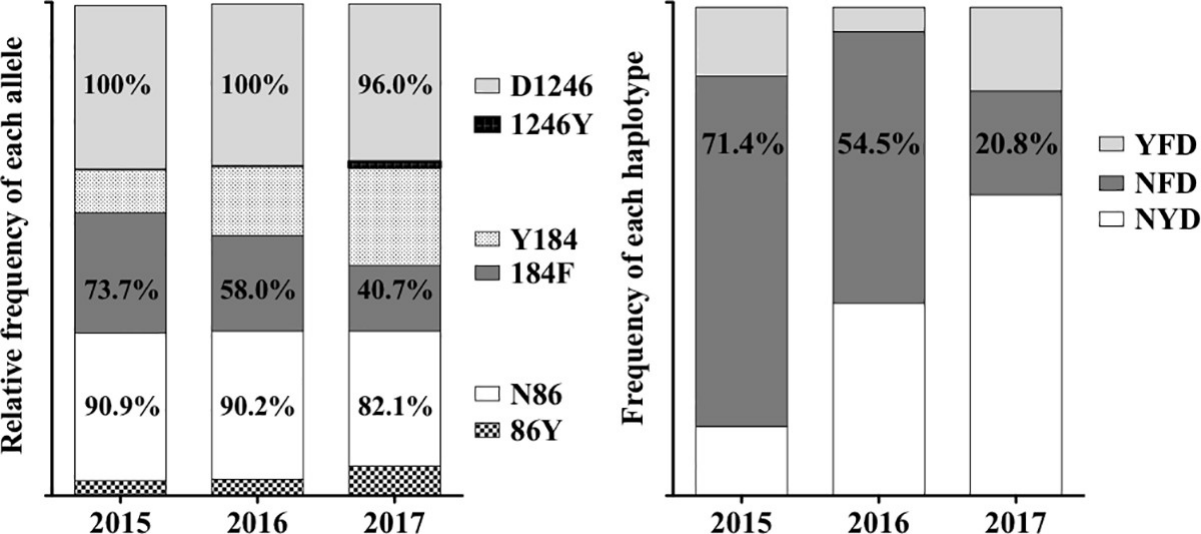
Temporal changes of prevalence at codons 86, 184 and 1246 of *pfmdr1*. Percentage (%) inserted in the graphic bars represents the prevalence of the alleles and haplotypes in each year.

A total of 101, 96 and 68 samples were successfully genotyped for *pfmdr1* codons 86, 184, and 1246, respectively. Mixed *pfmdr1* alleles were not observed. Overall, wild-type alleles predominated (Fig 2), except for codon 184 where the mutant allele 184F occurred more frequently (56.3%) than the wild-type allele Y184 (43.8%; Table 1). When analyzed separately over time, the prevalence of the mutant allele 184F decreased from 73.7% in 2015 to 58.0% in 2016 and to 40.7% in 2017 (Table 1).

For *pfmdr1*, only three haplotypes were identified with the following overall prevalence: NFD (45.0%), NYD (43.3%) and YFD (11.7%). No triple *pfmdr1* mutants were found at codon 86, 184, or 1246 (YFY). When compared between years, the prevalence of the triple haplotype NFD decreased significantly from 71.4% in 2015 to 54.5% in 2016 and 20.8% in 2017 (Fig 2).

## Discussion

Considering the trends of artemisinin decreasing activity [5–7], treatment efficacy should be monitored at regular intervals. Monitoring should be performed by directly measuring efficacy *in vivo*, or indirectly, by molecular markers surveillance, which can reveal drug response trends that inform on the possible therapeutic life-span of a given treatment [13]. In Senegal, ACTs (AL, ASAQ and DHA-piperaquine; DHA-PPQ) remain highly effective for the treatment of uncomplicated *P*. *falciparum* malaria. In the few reported cases of recrudescence after ACT treatment, parasite samples did not carry mutations in the K13-propeller domain [32]. These observations seem to follow the regional tendency.

In Senegal, there are currently no systematic (nationwide) epidemiological studies on molecular markers of drug resistance. Although a number of studies have been published in Senegal reporting mutations in *pfk13* [20,31,32,36–38,44], samples from those studies originate mostly from Thies and Dakar regions (near and around the capital). In one exception, a recent study reported *pfk13* SNPs from Diourbel (east of Thiés) and Kedougou, in the southeast of the country (Fig 1); [32]. Hence the importance of our study, which analyses samples from Bounkiling City, in the Sédhiou region in the south of Senegal (Fig 1). Our analysis revealed that all but 6 of the isolates carried wild type K13-propeller. This is in line with recent observations in Senegal, where most of the samples were also wild type for K13-propeller domain [17,32,38]. None of the 5 identified SNPs in our samples occurred in more than two samples (Table 1). Nevertheless one study including 207 isolates from Thiés and Dakar [17], identified a total of 22 SNPs in *pfk13* propeller domain (7 synonymous and 15 non-synonymous). In our work, we have identified only 6 polymorphisms 5 synonymous and one non-synonymous (Table 1). Most of these polymorphisms were limited to single isolates, suggesting that they are likely transient polymorphisms part of naturally evolving parasite populations. The fact that some of these polymorphisms tend to occur in the same positions, might indicate that certain regions of the gene are more disposed to variations then others. Also, *pfk13* artemisinin-resistance associated alleles being selected in Africa, may not be the same as the ones selected in Southeast Asia. The synonymous polymorphism C469C might be particularly relevant because, in this position, two variant amino acids (C469F and C469Y) have been previously associated with slow clearance of parasitemia, after ACT therapy [45,46]. Additionally, this polymorphism was also previously reported in samples from Thiés and Dakar in Senegal [17] and in Ghana [47,48]. In the two countries bordering Sedhiou, The Gambia to the north and Guinea-Bissau to the south (Fig 1), wild type *pfk13* is highly prevalent and SNPs associated with artemisinin resistance in Southeast Asia, have not been identified [19–21]. Nevertheless, monitoring the prevalence of these loci over time may help to infer which alleles are biologically relevant or selected under drug pressure.

The molecular marker *pfmdr1* 86Y (together with *pfcrt*, initially driven through the parasite population by the previous widespread use of chloroquine) has been decreasing in many parts of Africa. This declining prevalence is accelerated in countries using AL, consistent with the expected direction of selection [49,50]. Here we report a high prevalence of wild type alleles N86 and D1246, which follow the tendency reported from northern Senegal, namely from Thiés and Dakar [19,20,28,33,35,44,51–54], the neighbouring The Gambia [19,20,54], and other West Africa countries [16,55]. The *pfmdr1* 86Y is associated with a longer time to reinfection after AL treatment and a shorter time after ASAQ [49], reinforcing its informative value as a molecular marker of antimalarial drug susceptibility.

For codon 184, our analysis of *pfmdr1* revealed differences in the distribution of mutant allele between years. The high prevalence of 184F in 2015 (73.7%) decreased over time to 40.7% in 2017 (Fig 2; Table 1). Declining of 184F prevalence was previously reported in parasite samples collected between 2012 and 2014 in northern Senegal [20,31,51] and in The Gambia [20]. This decrease, in Senegal, was correlated with a local and temporary change to ASAQ as first the line of treatment [31]. Three *pfmdr1* haplotypes were identified in our study: NFD, NYD and YFD. The haplotype YYY was not identified; however, this was not surprising since YYY is believed to be selected under the pressure of DHA-PPQ treatment from a genetic background 86Y and Y184 [56,57]. DHA-PPQ has not been widely used in Senegal, except to compensate for antimalarial drug shortages in 2010 and 2011 [32]. Other studies have reported absence or low prevalence of the allele 1246Y [35] or the haplotype YYY haplotype in Senegal (northern regions) [31]. The two haplotypes NFD and NYD were the most frequent triple haplotypes found in our samples, as was the case in other studies from northern Senegal [31,51] in the neighbouring The Gambia [19,20] and generally in Africa [50]. These observations argue in favor of lumefantrine selecting the triple haplotype N86F184D1246 (NFD) [28,31,35,58–60] and that this selection might be sequential; first N86, second D1246 and the last 184F [24,50,61,62]. A high prevalence of haplotype NFD was observed in 2015 (71.4%) samples, however, a significant decrease was detected in 2017 (20%) driven by the decline of the mutant allele 184F (Fig 2). This decrease could be explained by therapy policy change, and/or intense migration of parasite populations to Bounkiling from other regions of Senegal or the neighboring The Gambia or Guinea-Bissau. Malaria therapy policy did not change in Bounkiling during 2016 and 2017, which could cause decreased in AL pressure hence accounting for the observed decrease in 184F frequency. Recently, a decreasing trend in frequency of the 184F allele around the same period, was reported from the neighboring Guinea-Bissau [21,63]. Hence intense migration of parasite populations to Bounkiling from Guinea-Bissau, could account (at least in part) for the observed 184F decreasing frequency trend. To the best of our knowledge, there is no published information on the prevalence of these markers between 2015 and 2017 (or since 2017) either from other regions in Senegal or from the neighboring The Gambia. A higher number of samples analyzed with next generation sequencing, would increase the robustness of our results. A national survey of molecular markers of drug resistance would help to clarify these results and better inform National Public Health Authorities on first-line treatment regimens to manage uncomplicated *P*. *falciparum* malaria.

## Conclusions

All isolates carried wild type *pfk13* gene, except for 6 samples that carried 5 different synonymous SNPs. Prevalence of haplotype NFD in pfmdr1 gene, decreased over time from 2015 to 2017. To our knowledge, this is the first work providing information on the prevalence of k13-propeller and *pfmdr1* mutations in Sédhiou, a region in the south of Senegal. We are contributing to the surveillance of molecular markers of drug resistance in Africa.

## Supporting information

S1 File Primers used in this study. (PDF)

S2 File Comprehensive data of isolate genotype for *pfmdr1* N86Y, Y184F and D1246Y. (PDF)

S3 File Comprehensive data showing sequence alignment for pfK13 with highlighted SNPs. (PDF)
