# Supplementary material for: Prevalence of pfk13 and pfmdr1 polymorphisms in Bounkiling, Southern Senegal
Source: PLoS One. 2021 Mar 26;16(3):e0249357. doi: 10.1371/journal.pone.0249357 (PMC7996989; doi:10.1371/journal.pone.0249357)
Supplement: S1 File — (PDF) [file pone.0249357.s001.pdf]

S1 : Primers used for *pfK13* fragment amplification

Forward	*3' GAAAGAAGCAGAATTTTATGG5'*
Reverse	3' GCTTGGCCCATCTTTATTAGTTCCC 5'
Forward	3' GTGTAGAATATTTAAATTCG 5'

Primers used for *pfmdr1* fragment amplification

Forward	3' GTATGTGCTGTATTATCAGGAGGA 5'
Reverse	3' TTAATTTATGTTTGTGGTGTCATATG 5'
