# Supplementary material for: Prevalence of pfk13 and pfmdr1 polymorphisms in Bounkiling, Southern Senegal
Source: PLoS One. 2021 Mar 26;16(3):e0249357. doi: 10.1371/journal.pone.0249357 (PMC7996989; doi:10.1371/journal.pone.0249357)
Supplement: S2 File — (PDF) [file pone.0249357.s002.pdf]

Samples from 2015, gene *pfmdr1* N86Y and Y184F

	201					256				300
CBF005_138	CAGGAGGAAC	ATTACCTTTT	TTTATATCTG	TGTTTGGTGT	AATATTAAAG	AACATGAATT	TAGGTGATGA	TATTAATCCT	ATAATATTAT	CATTAGTATC
CBE627_138	CAGGAGGAAC	ATTACCTTTT	TTTATATCTG	TGTTTGGTGT	AATATTAAAG	AACATGAATT	TAGGTGATGA	TATTAATCCT	ATAATATTAT	CATTAGTATC
CBE624_138	CAGGAGGAAC	ATTACCTTTT	TTTATATCTG	TGTTTGGTGT	AATATTAAAG	AACATGTATT	TAGGTGATGA	TATTAATCCT	ATAATATTAT	CATTAGTATC
CBE628_138	CAGGAGGAAC	ATTACCTTTT	TTTATATCTG	TGTTTGGTGT	AATATTAAAG	AACATGAATT	TAGGTGATGA	TATTAATCCT	ATAATATTAT	CATTAGTATC
CBE630_138	CAGGAGGAAC	ATTACCTTTT	TTTATATCTG	TGTTTGGTGT	AATATTAAAG	AACATGAATT	TAGGTGATGA	TATTAATCCT	ATAATATTAT	CATTAGTATC
CBF008_138	CAGGAGGAAC	ATTACCTTTT	TTTATATCTG	TGTTTGGTGT	AATATTAAAG	AACATGAATT	TAGGTGATGA	TATTAATCCT	ATAATATTAT	CATTAGTATC
CBE625_138	CAGGAGGAAC	ATTACCTTTT	TTTATATCTG	TGTTTGGTGT	AATATTAAAG	AACATGAATT	TAGGTGATGA	TATTAATCCT	ATAATATTAT	CATTAGTATC
CBE631_138	CAGGAGGAAC	ATTACCTTTT	TTTATATCTG	TGTTTGGTGT	AATATTAAAG	AACATGAATT	TAGGTGATGA	TATTAATCCT	ATAATATTAT	CATTAGTATC
CBE645_138	CAGGAGGAAC	ATTACCTTTT	TTTATATCTG	TGTTTGGTGT	AATATTAAAG	AACATGTATT	TAGGTGATGA	TATTAATCCT	ATAATATTAT	CATTAGTATC
**3D7**	**CAGGAGGAAC**	**ATTACCTTTT**	**TTTATATCTG**	**TGTTTGGTGT**	**AATATTAAAG**	**AACATGAATT**	**TAGGTGATGA**	**TATTAATCCT**	**ATAATATTAT**	**CATTAGTATC**
CBE634_138	CAGGAGGAAC	ATTACCTTTT	TTTATATCTG	TGTTTGGTGT	AATATTAAAG	AACATGAATT	TAGGTGATGA	TATTAATCCT	ATAATATTAT	CATTAGTATC
CBE632_138	CAGGAGGAAC	ATTACCTTTT	TTTATATCTG	TGTTTGGTGT	AATATTAAAG	AACATGAATT	TAGGTGATGA	TATTAATCCT	ATAATATTAT	CATTAGTATC
CBE639_138	CAGGAGGAAC	ATTACCTTTT	TTTATATCTG	TGTTTGGTGT	AATATTAAAG	AACATGAATT	TAGGTGATGA	TATTAATCCT	ATAATATTAT	CATTAGTATC
CBE626_138	CAGGAGGAAC	ATTACCTTTT	TTTATATCTG	TGTTTGGTGT	AATATTAAAG	AACATGAATT	TAGGTGATGA	TATTAATCCT	ATAATATTAT	CATTAGTATC
CBE640_138	CAGGAGGAAC	ATTACCTTTT	TTTATATCTG	TGTTTGGTGT	AATATTAAAG	AACATGAATT	TAGGTGATGA	TATTAATCCT	ATAATATTAT	CATTAGTATC
CBE641_138	CAGGAGGAAC	ATTACCTTTT	TTTATATCTG	TGTTTGGTGT	AATATTAAAG	AACATGAATT	TAGGTGATGA	TATTAATCCT	ATAATATTAT	CATTAGTATC
CBE629_138	CAGGAGGAAC	ATTACCTTTT	TTTATATCTG	TGTTTGGTGT	AATATTAAAG	AACATGAATT	TAGGTGATGA	TATTAATCCT	ATAATATTAT	CATTAGTATC
CBE633_138	CAGGAGGAAC	ATTACCTTTT	TTTATATCTG	TGTTTGGTGT	AATATTAAAG	AACATGTATT	TAGGTGATGA	TATTAATCCT	ATAATATTAT	CATTAGTATC
CBE636_138	CAGGAGGAAC	ATTACCTTTT	TTTATATCTG	TGTTTGGTGT	AATATTAAAG	AACATGAATT	TAGGTGATGA	TATTAATCCT	ATAATATTAT	CATTAGTATC
CBE516_138	CAGGAGGAAC	ATTACCTTTT	TTTATATCTG	TGTTTGGTGT	AATATTAAAG	AACATGAATT	TAGGTGATGA	TATTAATCCT	ATAATATTAT	CATTAGTATC
CBE521_138	CAGGAGGAAC	ATTACCTTTT	TTTATATCTG	TGTTTGGTGT	AATATTAAAG	AACATGAATT	TAGGTGATGA	TATTAATCCT	ATAATATTAT	CATTAGTATC
CBE539_138	CAGGAGGAAC	ATTACCTTTT	TTTATATCTG	TGTTTGGTGT	AATATTAAAG	AACATGAATT	TAGGTGATGA	TATTAATCCT	ATAATATTAT	CATTAGTATC
CBE658_138	CAGGAGGAAC	ATTACCTTTT	TTTATATCTG	TGTTTGGTGT	AATATTAAAG	AACATGAATT	TAGGTGATGA	TATTAATCCT	ATAATATTAT	CATTAGTATC
CBE538_138	CAGGAGGAAC	ATTACCTTTT	TTTATATCTG	TGTTTGGTGT	AATATTAAAG	AACATGAATT	TAGGTGATGA	TATTAATCCT	ATAATATTAT	CATTAGTATC

	301									400
CBF005_138	TATAGGTTTA	GTACAATTTA	TATTATCAAT	GATATCAAGT	TATTGTATGG	ATGTAATTAC	ATCAAAAATA	TTAAAAACTT	TAAAGCTTGA	ATATTTAAGA
CBE627_138	TATAGGTTTA	GTACAATTTA	TATTATCAAT	GATATCAAGT	TATTGTATGG	ATGTAATTAC	ATCAAAAATA	TTAAAAACTT	TAAAGCTTGA	ATATTTAAGA
CBE624_138	TATAGGTTTA	GTACAATTTA	TATTATCAAT	GATATCAAGT	TATTGTATGG	ATGTAATTAC	ATCAAAAATA	TTAAAAACTT	TAAAGCTTGA	ATATTTAAGA
CBE628_138	TATAGGTTTA	GTACAATTTA	TATTATCAAT	GATATCAAGT	TATTGTATGG	ATGTAATTAC	ATCAAAAATA	TTAAAAACTT	TAAAGCTTGA	ATATTTAAGA
CBE630_138	TATAGGTTTA	GTACAATTTA	TATTATCAAT	GATATCAAGT	TATTGTATGG	ATGTAATTAC	ATCAAAAATA	TTAAAAACTT	TAAAGCTTGA	ATATTTAAGA
CBF008_138	TATAGGTTTA	GTACAATTTA	TATTATCAAT	GATATCAAGT	TATTGTATGG	ATGTAATTAC	ATCAAAAATA	TTAAAAACTT	TAAAGCTTGA	ATATTTAAGA
CBE625_138	TATAGGTTTA	GTACAATTTA	TATTATCAAT	GATATCAAGT	TATTGTATGG	ATGTAATTAC	ATCAAAAATA	TTAAAAACTT	TAAAGCTTGA	ATATTTAAGA
CBE631_138	TATAGGTTTA	GTACAATTTA	TATTATCAAT	GATATCAAGT	TATTGTATGG	ATGTAATTAC	ATCAAAAATA	TTAAAAACTT	TAAAGCTTGA	ATATTTAAGA
CBE645_138	TATAGGTTTA	GTACAATTTA	TATTATCAAT	GATATCAAGT	TATTGTATGG	ATGTAATTAC	ATCAAAAATA	TTAAAAACTT	TAAAGCTTGA	ATATTTAAGA
**3D7**	**TATAGGTTTA**	**GTACAATTTA**	**TATTATCAAT**	**GATATCAAGT**	**TATTGTATGG**	**ATGTAATTAC**	**ATCAAAAATA**	**TTAAAAACTT**	**TAAAGCTTGA**	**ATATTTAAGA**
CBE634_138	TATAGGTTTA	GTACAATTTA	TATTATCAAT	GATATCAAGT	TATTGTATGG	ATGTAATTAC	ATCAAAAATA	TTAAAAACTT	TAAAGCTTGA	ATATTTAAGA
CBE632_138	TATAGGTTTA	GTACAATTTA	TATTATCAAT	GATATCAAGT	TATTGTATGG	ATGTAATTAC	ATCAAAAATA	TTAAAAACTT	TAAAGCTTGA	ATATTTAAGA
CBE639_138	TATAGGTTTA	GTACAATTTA	TATTATCAAT	GATATCAAGT	TATTGTATGG	ATGTAATTAC	ATCAAAAATA	TTAAAAACTT	TAAAGCTTGA	ATATTTAAGA
CBE626_138	TATAGGTTTA	GTACAATTTA	TATTATCAAT	GATATCAAGT	TATTGTATGG	ATGTAATTAC	ATCAAAAATA	TTAAAAACTT	TAAAGCTTGA	ATATTTAAGA
CBE640_138	TATAGGTTTA	GTACAATTTA	TATTATCAAT	GATATCAAGT	TATTGTATGG	ATGTAATTAC	ATCAAAAATA	TTAAAAACTT	TAAAGCTTGA	ATATTTAAGA
CBE641_138	TATAGGTTTA	GTACAATTTA	TATTATCAAT	GATATCAAGT	TATTGTATGG	ATGTAATTAC	ATCAAAAATA	TTAAAAACTT	TAAAGCTTGA	ATATTTAAGA
CBE629_138	TATAGGTTTA	GTACAATTTA	TATTATCAAT	GATATCAAGT	TATTGTATGG	ATGTAATTAC	ATCAAAAATA	TTAAAAACTT	TAAAGCTTGA	ATATTTAAGA
CBE633_138	TATAGGTTTA	GTACAATTTA	TATTATCAAT	GATATCAAGT	TATTGTATGG	ATGTAATTAC	ATCAAAAATA	TTAAAAACTT	TAAAGCTTGA	ATATTTAAGA
CBE636_138	TATAGGTTTA	GTACAATTTA	TATTATCAAT	GATATCAAGT	TATTGTATGG	ATGTAATTAC	ATCAAAAATA	TTAAAAACTT	TAAAGCTTGA	ATATTTAAGA
CBE516_138	TATAGGTTTA	GTACAATTTA	TATTATCAAT	GATATCAAGT	TATTGTATGG	ATGTAATTAC	ATCAAAAATA	TTAAAAACTT	TAAAGCTTGA	ATATTTAAGA
CBE521_138	TATAGGTTTA	GTACAATTTA	TATTATCAAT	GATATCAAGT	TATTGTATGG	ATGTAATTAC	ATCAAAAATA	TTAAAAACTT	TAAAGCTTGA	ATATTTAAGA
CBE539_138	TATAGGTTTA	GTACAATTTA	TATTATCAAT	GATATCAAGT	TATTGTATGG	ATGTAATTAC	ATCAAAAATA	TTAAAAACTT	TAAAGCTTGA	ATATTTAAGA

```
CBE658_138   TATAGGTTTA GTACAATTTA TATTATCAAT GATATCAAGT TATTGTATGG ATGGAATGTG AATTACGATC GAAGAAGA
CBE538_138   TATAGGTTTA GTACAATTTA TATTATCAAT GATATCAAGT TATTGTATGG ATGTAATTAC ATCAAAAATA TTAAAAACTT TAAAGCTTGA ATATTTA

             401                                                                                              500
CBF005_138   AGTGTTTTTT ATCAAGATGG ACAATTTCAT GATAATAATC CTGGATCTAA ATTAAGATCT GATTTAGATT TTTATTTAGA ACAAGTGAGT TCAGGAATTG
CBE627_138   AGTGTTTTTT ATCAAGATGG ACAATTTCAT GATAATAATC CTGGATCTAA ATTAAGATCT GATTTAGATT TTTATTTAGA ACAAGTGAGT TCAGGAATTG
CBE624_138   AGTGTTTTTT ATCAAGATGG ACAATTTCAT GATAATAATC CTGGATCTAA ATTAAGATCT GATTTAGATT TTTATTTAGA ACAAGTGAGT TCAGGAATTG
CBE628_138   AGTGTTTTTT ATCAAGATGG ACAATTTCAT GATAATAATC CTGGATCTAA ATTAAGATCT GATTTAGATT TTTATTTAGA ACAAGTGAGT TCAGGAATTG
CBE630_138   AGTGTTTTTT ATCAAGATGG ACAATTTCAT GATAATAATC CTGGATCTAA ATTAAGATCT GATTTAGATT TTTATTTAGA ACAAGTGAGT TCAGGAATTG
CBF008_138   AGTGTTTTTT ATCAAGATGG ACAATTTCAT GATAATAATC CTGGATCTAA ATTAAGATCT GATTTAGATT TTTATTTAGA ACAAGTGAGT TCAGGAATTG
CBE625_138   AGTGTTTTTT ATCAAGATGG ACAATTTCAT GATAATAATC CTGGATCTAA ATTAAGATCT GATTTAGATT TTTATTTAGA ACAAGTGAGT TCAGGAATTG
CBE631_138   AGTGTTTTTT ATCAAGATGG ACAATTTCAT GATAATAATC CTGGATCTAA ATTAAGATCT GATTTAGATT TTTATTTAGA ACAAGTGAGT TCAGGAATTG
CBE645_138   AGTGTTTTTT ATCAAGATGG ACAATTTCAT GATAATAATC CTGGATCTAA ATTAAGATCT GATTTAGATT TTTATTTAGA ACAAGTGAGT TCAGGAATTG
       3D7   AGTGTTTTTT ATCAAGATGG ACAATTTCAT GATAATAATC CTGGATCTAA ATTAAGATCT GATTTAGATT TTTATTTAGA ACAAGTGAGT TCAGGAATTG
CBE634_138   AGTGTTTTTT ATCAAGATGG ACAATTTCAT GATAATAATC CTGGATCTAA ATTAAGATCT GATTTAGATT TTTATTTAGA ACAAGTGAGT TCAGGAATTG
CBE632_138   AGTGTTTTTT ATCAAGATGG ACAATTTCAT GATAATAATC CTGGATCTAA ATTAAGATCT GATTTAGATT TTTATTTAGA ACAAGTGAGT TCAGGAATTG
CBE639_138   AGTGTTTTTT ATCAAGATGG ACAATTTCAT GATAATAATC CTGGATCTAA ATTAAGATCT GATTTAGATT TTTATTTAGA ACAAGTGAGT TCAGGAATTG
CBE626_138   AGTGTTTTTT ATCAAGATGG ACAATTTCAT GATAATAATC CTGGATCTAA ATTAAGATCT GATTTAGATT TTTATTTAGA ACAAGTGAGT TCAGGAATTG
CBE640_138   AGTGTTTTTT ATCAAGATGG ACAATTTCAT GATAATAATC CTGGATCTAA ATTAAGATCT GATTTAGATT TTTATTTAGA ACAAGTGAGT TCAGGAATTG
CBE641_138   AGTGTTTTTT ATCAAGATGG ACAATTTCAT GATAATAATC CTGGATCTAA ATTAAGATCT GATTTAGATT TTTATTTAGA ACAAGTGAGT TCAGGAATTG
CBE629_138   AGTGTTTTTT ATCAAGATGG ACAATTTCAT GATAATAATC CTGGATCTAA ATTAAGATCT GATTTAGATT TTTATTTAGA ACAAGTGAGT TCAGGAATTG
CBE633_138   AGTGTTTTTT ATCAAGATGG ACAATTTCAT GATAATAATC CTGGATCTAA ATTAAGATCT GATTTAGATT TTTATTTAGA ACAAGTGAGT TCAGGAATTG
CBE636_138   AGTGTTTTTT ATCAAGATGG ACAATTTCAT GATAATAATC CTGGATCTAA ATTAAGATCT GATTTAGATT TTTATTTAGA ACAAGTGAGT TCAGGAATTG
CBE516_138   AGTGTTTTTT ATCAAGATGG ACAATTTCAT GATAATAATC CTGGATCTAA ATTAAGATCT GATTTAGATT TTTATTTAGA ACAAGTGAGT TCAGGAATTG
CBE521_138   AGTGTTTTTT ATCAAGATGG ACAATTTCAT GATAATAATC CTGGATCTAA ATTAAGATCT GATTTAGATT TTTATTTAGA ACAAGTGAGT TCAGGAATTG
CBE539_138   AGTGTTTTTT ATCAAGATGG ACAATTTCAT GATAATAATC CTGGATCTAA ATTAAGATCT GATTTAGATT TTTATTTAGA ACAAGTGAGT TCGCGGA
CBE658_138
CBE538_138

             501                                                    551                                       600
CBF005_138   GTACGAAATT TATAACAATT TTTACATATG CCAGTTCCTT TTTAGGTTTA TTTATTTGGT CATTAATAAA AAATGCACGT TTGACTTTAT GTATTACTTG
CBE627_138   GTACGAAATT TATAACAATT TTTACATATG CCAGTTCCTT TTTAGGTTTA TATATTTGGT CATTAATAAA AAATGCACGT TTGACTTTAT GTATTACTTG
CBE624_138   GTACGAAATT TATAACAATT TTTACATATG CCAGTTCCTT TTTAGGTTTA TTTATTTGGT CATTAATAAA AAATGCACGT TTGACTTTAT GTATTACTTG
CBE628_138   GTACGAAATT TATAACAATT TTTACATATG CCAGTTCCTT TTTAGGTTTA TTTATTTGGT CATTAATAAA AAATGCACGT TTGACTTTAT GTATTACTTG
CBE630_138   GTACGAAATT TATAACAATT TTTACATATG CCAGTTCCTT TTTAGGTTTA TTTATTTGGT CATTAATAAA AAATGCACGT TTGACTTTAT GTATTACTTG
CBF008_138   GTACGAAATT TATAACAATT TTTACATATG CCAGTTCCTT TTTAGGTTTA TATATTTGGT CATTAATAAA AAATGCACGT TTGACTTTAT GTATTACTTG
CBE625_138   GTACGAAATT TATAACAATT TTTACATATG CCAGTTCCTT TTTAGGTTTA TATATTTGGT CATTAATAAA AAATGCACGT TTGACTTTAT GTATTACTTG
CBE631_138   GTACGAAATT TATAACAATT TTTACATATG CCAGTTCCTT TTTAGGTTTA TATATTTGGT CATTAATAAA AAATGCACGT TTGACTTTAT GTATTACTTG
CBE645_138   GTACGAAATT TATAACAATT TTTACATATG CCAGTTCCTT TTTAGGTTTA TTTATTTGGT CATTAATAAA AAATGCACGT TTGACTTTAT GTATTACTTG
       3D7   GTACGAAATT TATAACAATT TTTACATATG CCAGTTCCTT TTTAGGTTTA TATATTTGGT CATTAATAAA AAATGCACGT TTGACTTTAT GTATTACTTG
CBE634_138   GTACGAAATT TATAACAATT TTTACATATG CCAGTTCCTT TTTAGGTTTA TATATTTGGT CATTAATAAA AAATGCACGT TTGACTTTAT GTATTACTTG
CBE632_138   GTACGAAATT TATAACAATT TTTACATATG CCAGTTCCTT TTTAGGTTTA TTTATTTGGT CATTAATAAA AAATGCACGT TTGACTTTAT GTATTACTTG
CBE639_138   GTACGAAATT TATAACAATT TTTACATATG CCAGTTCCTT TTTAGGTTTA TATATTTGGT CATTAATAAA AAATGCACGT TTGACTTTAT GTATTACTTG
CBE626_138   GTACGAAATT TATAACAATT TTTACATATG CCAGTTCCTT TTTAGGTTTA TTTATTTGGT CATTAATAAA AAATGCACGT TTGACTTTAT GTATTACTTG
CBE640_138   GTACGAAATT TATAACAATT TTTACATATG CCAGTTCCTT TTTAGGTTTA TTTATTTGGT CATTAATAAA AAATGCACGT TTGACTTTAT GTATTACTTG
CBE641_138   GTACGAAATT TATAACAATT TTTACATATG CCAGTTCCTT TTTAGGTTTA TTTATTTGGT CATTAATAAA AAATGCACGT TTGACTTTAT GTATTACTTG
CBE629_138   GTACGAAATT TATAACAATT TTTACATATG CCAGTTCCTT TTTAGGTTTA TTTATTTGGT CATTAATAAA AAATGCACGT TTGACTTTAT GTATTACTTG
CBE633_138   GTACGAAATT TATAACAATT TTTACATATG CCAGTTCCTT TTTAGGTTTA TTTATTTGGT CATTAATAAA AAATGCACGT TTGACTTTAT GTATTACTTG
CBE636_138   GTACGAAATT TATAACAATT TTTACATATG CCAGTTCCTT TTTAGGTTTA TATATTTGGT CATTAATAAA AAATGCACGT TTGACTTTAT GTATTACTTG
CBE516_138   GTACGAAATT TATAACAATT TTTACATATG CCAGTTCCTT TTTAGGTTTA TTTATTTGGT CATTAATAAA AAATGCACGT TTGACTTTAA GGGTAATT
CBE521_138   GTACGAAATT TATAACAATT TTTACATATG CCAGTTCCTT TTTAGGTTTA TTTATTTGGT CATTAATAAA AAATGCACGT TTGACTTTAT
```

```
CBE539_138
CBE658_138
CBE538_138
```

Samples from 2015, gene *pfmdr1* D1246Y

```
            3601                                                                                                     3700
CBE656_138  ATAATAATTC ATTGGTTTTA AAAAATGTAA ATGAATTTTC AAACCAATCT GGATCTGCAG AAGATTATAC TGTATTTAAT AATAATGGAG AAATATTATT
CBE668_138  ATAATAATTC ATTGGTTTTA AAAAATGTAA ATGAATTTTC AAACCAATCT GGATCTGCAG AAGATTATAC TGTATTTAAT AATAATGGAG AAATATTATT
CBE672_138  ATAATAATTC ATTGGTTTTA AAAAATGTAA ATGAATTTTC AAACCAATCT GGATCTGCAG AAGATTATAC TGTATTTAAT AATAATGGAG AAATATTATT
CBE654_138  ATAATAATTC ATTGGTTTTA AAAAATGTAA ATGAATTTTC AAACCAATCT GGATCTGCAG AAGATTATAC TGTATTTAAT AATAATGGAG AAATATTATT
CBE659_138  ATAATAATTC ATTGGTTTTA AAAAATGTAA ATGAATTTTC AAACCAATCT GGATCTGCAG AAGATTATAC TGTATTTAAT AATAATGGAG AAATATTATT
CBE667_138  ATAATAATTC ATTGGTTTTA AAAAATGTAA ATGAATTTTC AAACCAATCT GGATCTGCAG AAGATTATAC TGTATTTAAT AATAATGGAG AAATATTATT
CBE662_138  ATAATAATTC ATTGGTTTTA AAAAATGTAA ATGAATTTTC AAACCAATCT GGATCTGCAG AAGATTATAC TGTATTTAAT AATAATGGAG AAATATTATT
CBE678_138  ATAATAATTC ATTGGTTTTA AAAAATGTAA ATGAATTTTC AAACCAATCT GGATCTGCAG AAGATTATAC TGTATTTAAT AATAATGGAG AAATATTATT
CBE664_138  ATAATAATTC ATTGGTTTTA AAAAATGTAA ATGAATTTTC AAACCAATCT GGATCTGCAG AAGATTATAC TGTATTTAAT AATAATGGAG AAATATTATT
       3D7  ATAATAATTC ATTGGTTTTA AAAAATGTAA ATGAATTTTC AAACCAATCT GGATCTGCAG AAGATTATAC TGTATTTAAT AATAATGGAG AAATATTATT
CBE657_138  ATAATAATTC ATTGGTTTTA AAAAATGTAA ATGAATTTTC AAACCAATCT GGATCTGCAG AAGATTATAC TGTATTTAAT AATAATGGAG AAATATTATT
CBE660_138  ATAATAATTC ATTGGTTTTA AAAAATGTAA ATGAATTTTC AAACCAATCT GGATCTGCAG AAGATTATAC TGTATTTAAT AATAATGGAG AAATATTATT
CBE658_138  ATAATAATTC ATTGGTTTTA AAAAATGTAA ATGAATTTTC AAACCAATCT GGATCTGCAG AAGATTATAC TGTATTTAAT AATAATGGAG AAATATTATT
CBE663_138  ATAATAATTC ATTGGTTTTA AAAAATGTAA ATGAATTTTC AAACCAATCT GGATCTGCAG AAGATTATAC TGTATTTAAT AATAATGGAG AAATATTATT
CBE674_138  ATAATAATTC ATTGGTTTTA AAAAATGTAA ATGAATTTTC AAACCAATCT GGATCTGCAG AAGATTATAC TGTATTTAAT AATAATGGAG AAATATTATT
CBE680_138  ATAATAATTC ATTGGTTTTA AAAAATGTAA ATGAATTTTC AAACCAATCT GGATCTGCAG AAGATTATAC TGTATTTAAT AATAATGGAG AAATATTATT
CBE669_138         TAC ATTGGTTTTA AAAAATGTAA ATGAATTTTC AAACCAATCT GGATCTGCAG AAGATTATAC TGTATTTAAT AATAATGGAG AAATATTATT
CBE655_138                                A ATGAATTTTC AAACCAATCT GGATCTGCAG AAGATTATAC TGTATTTAAT AATAATGGAG AAATATTATT
CBE696_138                                A ATGAATTTTC AAACCAATCT GGATCTGCAG AAGATTATAC TGTATTTAAT AATAATGGAG AAATATTATT
CBE661_138                                                                                                 TGGAT AAATATTATT

            3701                                 3738                                                                3800
CBE656_138  AGATGATATT AATATATGTG ATTATAACTT AAGAGATCTT AGAAACTTAT TTTCAATAGT TAGTCAAGAA CCCATGTTAT TTAATATGTC CATATATGAA
CBE668_138  AGATGATATT AATATATGTG ATTATAACTT AAGAGATCTT AGAAACTTAT TTTCAATAGT TAGTCAAGAA CCCATGTTAT TTAATATGTC CATATATGAA
CBE672_138  AGATGATATT AATATATGTG ATTATAACTT AAGAGATCTT AGAAACTTAT TTTCAATAGT TAGTCAAGAA CCCATGTTAT TTAATATGTC CATATATGAA
CBE654_138  AGATGATATT AATATATGTG ATTATAACTT AAGAGATCTT AGAAACTTAT TTTCAATAGT TAGTCAAGAA CCCATGTTAT TTAATATGTC CATATATGAA
CBE659_138  AGATGATATT AATATATGTG ATTATAACTT AAGAGATCTT AGAAACTTAT TTTCAATAGT TAGTCAAGAA CCCATGTTAT TTAATATGTC CATATATGAA
CBE667_138  AGATGATATT AATATATGTG ATTATAACTT AAGAGATCTT AGAAACTTAT TTTCAATAGT TAGTCAAGAA CCCATGTTAT TTAATATGTC CATATATGAA
CBE662_138  AGATGATATT AATATATGTG ATTATAACTT AAGAGATCTT AGAAACTTAT TTTCAATAGT TAGTCAAGAA CCCATGTTAT TTAATATGTC CATATATGAA
CBE678_138  AGATGATATT AATATATGTG ATTATAACTT AAGAGATCTT AGAAACTTAT TTTCAATAGT TAGTCAAGAA CCCATGTTAT TTAATATGTC CATATATGAA
CBE664_138  AGATGATATT AATATATGTG ATTATAACTT AAGAGATCTT AGAAACTTAT TTTCAATAGT TAGTCAAGAA CCCATGTTAT TTAATATGTC CATATATGAA
       3D7  AGATGATATT AATATATGTG ATTATAACTT AAGAGATCTT AGAAACTTAT TTTCAATAGT TAGTCAAGAA CCCATGTTAT TTAATATGTC CATATATGAA
CBE657_138  AGATGATATT AATATATGTG ATTATAACTT AAGAGATCTT AGAAACTTAT TTTCAATAGT TAGTCAAGAA CCCATGTTAT TTAATATGTC CATATATGAA
CBE660_138  AGATGATATT AATATATGTG ATTATAACTT AAGAGATCTT AGAAACTTAT TTTCAATAGT TAGTCAAGAA CCCATGTTAT TTAATATGTC CATATATGAA
CBE658_138  AGATGATATT AATATATGTG ATTATAACTT AAGAGATCTT AGAAACTTAT TTTCAATAGT TAGTCAAGAA CCCATGTTAT TTAATATGTC CATATATGAA
CBE663_138  AGATGATATT AATATATGTG ATTATAACTT AAGAGATCTT AGAAACTTAT TTTCAATAGT TAGTCAAGAA CCCATGTTAT TTAATATGTC CATATATGAA
CBE674_138  AGATGATATT AATATATGTG ATTATAACTT AAGAGATCTT AGAAACTTAT TTTCAATAGT TAGTCAAGAA CCCATGTTAT TTAATATGTC CATATATGAA
```

CBE680_138 AGATGATATT AATATATGTG ATTATAACTT AAGAGATCTT AGAAACTTAT TTTCAATAGT TAGTCAAGAA CCCATGTTAT TTAATATGTC CATATATGAA
CBE669_138 AGATGATATT AATATATGTG ATTATAACTT AAGAGATCTT AGAAACTTAT TTTCAATAGT TAGTCAAGAA CCCATGTTAT TTAATATGTC CATATATGAA
CBE655_138 AGATGATATT AATATATGTG ATTATAACTT AAGAGATCTT AGAAACTTAT TTTCAATAGT TAGTCAAGAA CCCATGTTAT TTAATATGTC CATATATGAA
CBE696_138 AGATGATATT AATATATGTG ATTATAACTT AAGAGATCTT AGAAACTTAT TTTCAATAGT TAGTCAAGAA CCCATGTTAT TTAATATGTC CATATATGAA
CBE661_138 AGATGATATT AATATATGTG ATTATAACTT AAGAGATCTT AGAAACTTAT TTTCAATAGT TAGTCAAGAA CCCATGTTAT TTAATATGTC CATATATGAA

3801 3900
CBE656_138 AATATCAAAT TTGGAAGAGA AGATGCAACA TTGGAAGATG TTAAACGTGT TAGTAAGTTT GCTGCTATAG ATGAATTTAT CGAATCATTA CCAAATAAAT
CBE668_138 AATATCAAAT TTGGAAGAGA AGATGCAACA TTGGAAGATG TTAAACGTGT TAGTAAGTTT GCTGCTATAG ATGAATTTAT CGAATCATTA CCAAATAAAT
CBE672_138 AATATCAAAT TTGGAAGAGA AGATGCAACA TTGGAAGATG TTAAACGTGT TAGTAAGTTT GCTGCTATAG ATGAATTTAT CGAATCATTA CCAAATAAAT
CBE654_138 AATATCAAAT TTGGAAGAGA AGATGCAACA TTGGAAGATG TTAAACGTGT TAGTAAGTTT GCTGCTATAG ATGAATTTAT CGAATCATTA CCAAATAAAT
CBE659_138 AATATCAAAT TTGGAAGAGA AGATGCAACA TTGGAAGATG TTAAACGTGT TAGTAAGTTT GCTGCTATAG ATGAATTTAT CGAATCATTA CCAAATAAAT
CBE667_138 AATATCAAAT TTGGAAGAGA AGATGCAACA TTGGAAGATG TTAAACGTGT TAGTAAGTTT GCTGCTATAG ATGAATTTAT CGAATCATTA CCAAATAAAT
CBE662_138 AATATCAAAT TTGGAAGAGA AGATGCAACA TTGGAAGATG TTAAACGTGT TAGTAAGTTT GCTGCTATAG ATGAATTTAT CGAATCATTA CCAAATAAAT
CBE678_138 AATATCAAAT TTGGAAGAGA AGATGCAACA TTGGAAGATG TTAAACGTGT TAGTAAGTTT GCTGCTATAG ATGAATTTAT CGAATCATTA CCAAATAAAT
CBE664_138 AATATCAAAT TTGGAAGAGA AGATGCAACA TTGGAAGATG TTAAACGTGT TAGTAAGTTT GCTGCTATAG ATGAATTTAT CGAATCATTA CCAAATAAAT
3D7 AATATCAAAT TTGGAAGAGA AGATGCAACA TTGGAAGATG TTAAACGTGT TAGTAAGTTT GCTGCTATAG ATGAATTTAT CGAATCATTA CCAAATAAAT
CBE657_138 AATATCAAAT TTGGAAGAGA AGATGCAACA TTGGAAGATG TTAAACGTGT TAGTAAGTTT GCTGCTATAG ATGAATTTAT CGAATCATTA CCAAATAAAT
CBE660_138 AATATCAAAT TTGGAAGAGA AGATGCAACA TTGGAAGATG TTAAACGTGT TAGTAAGTTT GCTGCTATAG ATGAATTTAT CGAATCATTA CCAAATAAAT
CBE658_138 AATATCAAAT TTGGAAGAGA AGATGCAACA TTGGAAGATG TTAAACGTGT TAGTAAGTTT GCTGCTATAG ATGAATTTAT CGAATCATTA CCAAATAAAT
CBE663_138 AATATCAAAT TTGGAAGAGA AGATGCAACA TTGGAAGATG TTAAACGTGT TAGTAAGTTT GCTGCTATAG ATGAATTTAT CGAATCATTA CCAAATAAAT
CBE674_138 AATATCAAAT TTGGAAGAGA AGATGCAACA TTGGAAGATG TTAAACGTGT TAGTAAGTTT GCTGCTATAG ATGAATTTAT CGAATCATTA CCAAATAAAT
CBE680_138 AATATCAAAT TTGGAAGAGA AGATGCAACA TTGGAAGATG TTAAACGTGT TAGTAAGTTT GCTGCTATAG ATGAATTTAT CGAATCATTA CCAAATAAAT
CBE669_138 AATATCAAAT TTGGAAGAGA AGATGCAACA TTGGAAGATG TTAAACGTGT TAGTAAGTTT GCTGCTATAG ATGAATTTAT CGAATCATTA CCAAATAAAT
CBE655_138 AATATCAAAT TTGGAAGAGA AGATGCAACA TTGGAAGATG TTAAACGTGT TAGTAAGTTT GCTGCTATAG ATGAATTTAT CGAATCATTA CCAAATAAAT
CBE696_138 AATATCAAAT TTGGAAGAGA AGATGCAACA TTGGAAGATG TTAAACGTGT TAGTAAGTTT GCTGCTATAG ATGAATTTAT CGAATCATTA CCAAATAAAT
CBE661_138 AATATCAAAT TTGGAAGAGA AGATGCAACA TTGGAAGATG TTAAACGTGT TAGTAAGTTT GCTGCTATAG ATGAATTTAT CGAATCATTA CCAAATAAAT

Samples from 2016, gene *pfmdr1* N86Y and Y184F

201 258 300
CBE574_138 AGGAGGAACA TTACCTTTTT TTATATCTGT GTTTGGTGTA ATATTAAAGA ACATGAATTT AGGTGATGAT ATTAATCCTA TAATATTATC ATTAGTATCT
CBE577_138 AGGAGGAACA TTACCTTTTT TTATATCTGT GTTTGGTGTA ATATTAAAGA ACATGAATTT AGGTGATGAT ATTAATCCTA TAATATTATC ATTAGTATCT
3D7 AGGAGGAACA TTACCTTTTT TTATATCTGT GTTTGGTGTA ATATTAAAGA ACATGAATTT AGGTGATGAT ATTAATCCTA TAATATTATC ATTAGTATCT
CBE573_138 AGGAGGAACA TTACCTTTTT TTATATCTGT GTTTGGTGTA ATATTAAAGA ACATGAATTT AGGTGATGAT ATTAATCCTA TAATATTATC ATTAGTATCT
CBE576_138 AGGAGGAACA TTACCTTTTT TTATATCTGT GTTTGGTGTA ATATTAAAGA ACATGTATTT AGGTGATGAT ATTAATCCTA TAATATTATC ATTAGTATCT
CBE604_138 AGGAGGAACA TTACCTTTTT TTATATCTGT GTTTGGTGTA ATATTAAAGA ACATGAATTT AGGTGATGAT ATTAATCCTA TAATATTATC ATTAGTATCT
CBE572_138 AGGAGGAACA TTACCTTTTT TTATATCTGT GTTTGGTGTA ATATTAAAGA ACATGAATTT AGGTGATGAT ATTAATCCTA TAATATTATC ATTAGTATCT
CBE569_138 AGGAGGAACA TTACCTTTTT TTATATCTGT GTTTGGTGTA ATATTAAAGA ACATGAATTT AGGTGATGAT ATTAATCCTA TAATATTATC ATTAGTATCT
CBE570_138 AGGAGGAACA TTACCTTTTT TTATATCTGT GTTTGGTGTA ATATTAAAGA ACATGAATTT AGGTGATGAT ATTAATCCTA TAATATTATC ATTAGTATCT
CBE606_138 AGGAGGAACA TTACCTTTTT TTATATCTGT GTTTGGTGTA ATATTAAAGA ACATGAATTT AGGTGATGAT ATTAATCCTA TAATATTATC ATTAGTATCT
CBE581_138 AGGAGGAACA TTACCTTTTT TTATATCCTG GTTTGGTGTA ATATTAAAGA ACATGAATTT AGGTGATGAT ATTAATCCTA TAATATTATC ATTAGTATCT
CBE579_138 AGGAGGAACA TTACCTTTTT TTATATCTGT GTTTGGTGTA ATATTAAAGA ACATGAATTT AGGTGATGAT ATTAATCCTA TAATATTATC ATTAGTATCT
CBE618_138 AGGAGGAACA TTACCTTTTT TTATATCTGT GTTTGGTGTA ATATTAAAGA ACATGAATTT AGGTGATGAT ATTAATCCTA TAATATTATC ATTAGTATCT
CBE614_138 AGGAGGAACA TTACCTTTTT TTATATCTGT GTTTGGTGTA ATATTAAAGA ACATGAATTT AGGTGATGAT ATTAATCCTA TAATATTATC ATTAGTATCT

CBE617_138	AGGAGGAACA	TTACCTTTTT	TTATATCTGT	GTTTGGTGTA	ATATTAAAGA	ACATGAATTT	AGGTGATGAT	ATTAATCCTA	TAATATTATC	ATTAGTATCT
CBE620_138	AGGAGGAACA	TTACCTTTTT	TTATATCTGT	GTTTGGTGTA	ATATTAAAGA	ACATGAATTT	AGGTGATGAT	ATTAATCCTA	TAATATTATC	ATTAGTATCT
CBE597_138	AGGAGGAACA	TTACCTTTTT	TTATATCTGT	GTTTGGTGTA	ATATTAAAGA	ACATGAATTT	AGGTGATGAT	ATTAATCCTA	TAATATTATC	ATTAGTATCT
CBE586_138	AGGAGGAACA	TTACCTTTTT	TTATATCTGT	GTTTGGTGTA	ATATTAAAGA	ACATGAATTT	AGGTGATGAT	ATTAATCCTA	TAATATTATC	ATTAGTATCT
CBE612_138	AGGAGGAACA	TTACCTTTTT	TTATATCTGT	GTTTGGTGTA	ATATTAAAGA	ACATGAATTT	AGGTGATGAT	ATTAATCCTA	TAATATTATC	ATTAGTATCT
CBE583_138	AGGAGGAACA	TTACCTTTTT	TTATATCTGT	GTTTGGTGTA	ATATTAAAGA	ACATGTATTT	AGGTGATGAT	ATTAATCCTA	TAATATTATC	ATTAGTATCT
CBE599_138	AGGAGGAACA	TTACCTTTTT	TTATATCTGT	GTTTGGTGTA	ATATTAAAGA	ACATGAATTT	AGGTGATGAT	ATTAATCCTA	TAATATTATC	ATTAGTATCT
CBE602_138	AGGAGGAACA	TTACCTTTTT	TTATATCTGT	GTTTGGTGTA	ATATTAAAGA	ACATGAATTT	AGGTGATGAT	ATTAATCCTA	TAATATTATC	ATTAGTATCT
CBE607_138	AGGAGGAACA	TTACCTTTTT	TTATATCTGT	GTTTGGTGTA	ATATTAAAGA	ACATGAATTT	AGGTGATGAT	ATTAATCCTA	TAATATTATC	ATTAGTATCT
CBE568_138	AGGAGGAACA	TTACCTTTTT	TTATATCTGT	GTTTGGTGTA	ATATTAAAGA	ACATGAATTT	AGGTGATGAT	ATTAATCCTA	TAATATTATC	ATTAGTATCT
CBE589_138	AGGAGGAACA	TTACCTTTTT	TTATATCTGT	GTTTGGTGTA	ATATTAAAGA	ACATGTATTT	AGGTGATGAT	ATTAATCCTA	TAATATTATC	ATTAGTATCT
CBE605_138	AGGAGGAACA	TTACCTTTTT	TTATATCTGT	GTTTGGTGTA	ATATTAAAGA	ACATGAATTT	AGGTGATGAT	ATTAATCCTA	TAATATTATC	ATTAGTATCT
CBE611_138	AGGAGGAACA	TTACCTTTTT	TTATATCTGT	GTTTGGTGTA	ATATTAAAGA	ACATGAATTT	AGGTGATGAT	ATTAATCCTA	TAATATTATC	ATTAGTATCT
CBE622_138	AGGAGGAACA	TTACCTTTTT	TTATATCTGT	GTTTGGTGTA	ATATTAAAGA	ACATGAATTT	AGGTGATGAT	ATTAATCCTA	TAATATTATC	ATTAGTATTT
CBE585_138	AGGAGGAACA	TTACCTTTTT	TTATATCTGT	GTTTGGTGTA	ATATTAAAGA	ACATGAATTT	AGGTGATGAT	ATTAATCCTA	TAATATTATC	ATTAGTATCT
CBE619_138	AGGAGGAACA	TTACCTTTTT	TTATATCTGT	GTTTGGTGTA	ATATTAAAGA	ACATGAATTT	AGGTGATGAT	ATTAATCCTA	TAATATTATC	ATTAGTATCT
CBE587_138	AGGAGGAACA	TTACCTTTTT	TTATATCTGT	GTTTGGTGTA	ATATTAAAGA	ACATGAATTT	AGGTGATGAT	ATTAATCCTA	TAATATTATC	ATTAGTATCT
CBE648_138	AGGAGGAACA	TTACCTTTTT	TTATATCTGT	GTTTGGTGTA	ATATTAAAGA	ACATGAATTT	AGGTGATGAT	ATTAATCCTA	TAATATTATC	ATTAGTATCT
CBE652_138	AGGAGGAACA	TTACCTTTTT	TTATATCTGT	GTTTGGTGTA	ATATTAAAGA	ACATGAATTT	AGGTGATGAT	ATTAATCCTA	TAATATTATC	ATTAGTATCT
CBE593_138	AGGAGGAACA	TTACCTTTTT	TTATATCTGT	GTTTGGTGTA	ATATTAAAGA	ACATGAATTT	AGGTGATGAT	ATTAATCCTA	TAATATTATC	ATTAGTATCT
CBE590_138	AGGAGGAACA	TTACCTTTTT	TTATATCTGT	GTTTGGTGTA	ATATTAAAGA	ACATGAATTT	AGGTGATGAT	ATTAATCCTA	TAATATTATC	ATTAGTATCT
CBE592_138	AGGAGGAACA	TTACCTTTTT	TTATATCTGT	GTTTGGTGTA	ATATTAAAGA	ACATGAATTT	AGGTGATGAT	ATTAATCCTA	TAATATTATC	ATTAGTATCT
CBE601_138	AGGAGGAACA	TTACCTTTTT	TTATATCCTG	GTTTGGTGTA	ATATTAAAGA	ACATGAATTT	AGGTGATGAT	ATTAATCCTA	TAATATTATC	ATTAGTATCT
CBE609_138	AGGAGGAACA	TTACCTTTTT	TTATATCTGT	GTTTGGTGTA	ATATTAAAGA	ACATGAATTT	AGGTGATGAT	ATTAATCCTA	TAATATTATC	ATTAGTATCT
CBE610_138	AGGAGGAACA	TTACCTTTTT	TTATATCTGT	GTTTGGTGTA	ATATTAAAGA	ACATGAATTT	AGGTGATGAT	ATTAATCCTA	TAATATTATC	ATTAGTATCT
CBE616_138	AGGAGGAACA	TTACCTTTTT	TTATATCTGT	GTTTGGTGTA	ATATTAAAGA	ACATGAATTT	AGGTGATGAT	ATTAATCCTA	TAATATTATC	ATTAGTATCT
CBE575_138	AGGAGGAACA	TTACCTTTTT	TTATATCTGT	GTTTGGTGTA	ATATTAAAGA	ACATGAATTT	AGGTGATGAT	ATTAATCCTA	TAATATTATC	ATTAGTATCT
CBE598_138	AGGAGGAACA	TTACCTTTTT	TTATATACGT	GTTTGGTGTA	ATATTAAAGA	ACATGAATTT	AGGTGATGAT	ATTAATCCTA	TAATATTATC	ATTAGTATCT
CBE608_138	AGGAGGAACA	TTACCTTTTT	TTATATCTGT	GTTTGGTGTA	ATATTAAAGA	ACATGAATTT	AGGTGATGAT	ATTAATCCTA	TAATATTATC	ATTAGTATCT
CBE580_138	AGGAGGAACA	TTACCTTTTT	TTATATCCGT	GTTTGGTGTA	ATATTAAAGA	ACATGAATTT	AGGTGATGAT	ATTAATCCTA	TAATATTATC	ATTAGTATCT
CBE649_138	AGGAGGAACA	TTACCTTTTT	TGATATCTGT	GTTTGGTGTA	ATATTAAAGA	ACATGTATTT	AGGTGATGAT	ATTAATCCTA	TAATATTATC	ATTAGTATCT
CBE650_138	AGGAGGAACA	TTACCTTTTT	TTATATCTGT	GTTTGGTGTA	ATATTAAAGA	ACATGAATTT	AGGTGATGAT	ATTAATCCTA	TAATATTATC	ATTAGTATCT
CBE651_138	AGGAGGAACA	TTACCTTTTT	TTATATCTGT	GTTTGGAGTA	ATATTAAAGA	ACATGAATTT	AGGTGATGAT	ATTAATCCTA	TAATATTATC	ATTAGTATCT
CBE584_138	AGGAGGAACA	TTACCTTTTT	TTATATCTGT	GTTTGGTGTA	ATATTAAAGA	ACATGAATTT	AGGTGATGAT	ATTAATCCTA	TAATATTATC	ATTAGTATCT
CBE644_138	AGGAGGAACA	TTACCTTCCC	CTATATCTGT	GTTCGGTGTA	ATATTAAAGA	ACATGAATTT	AGGTGATGAT	ATTAATCCTA	TAATATTATC	ATTAGTATCT
CBE643_138	AGGAGGAACA	TTACCTTTTT	TTATATCTGT	GTTTGGTGTA	ATATTAAAGA	ACATGAATTT	AGGTGATGAT	ATTAATCCTA	TAATATTATC	ATTAGTATCT
CBE582_138	GAACG	TTACCTTTTT	TTATATCTGT	GTGTGGTGTA	ATATTAAAGA	ACGTGTATTT	AGGTG-TGAT	ATTAATCCTA	TAATATTATC	ATTAGTATCT
CBE621_138					ATATTAAAGA	ACATGAATTT	AGGTGATGAT	ATTAATCCTA	TAATATTATC	ATTAGTATCT

	301									400
CBE574_138	ATAGGTTTAG	TACAATTTAT	ATTATCAATG	ATATCAAGTT	ATTGTATGGA	TGTAATTACA	TCAAAAATAT	TAAAAACTTT	AAAGCTTGAA	TATTTAAGAA
CBE577_138	ATAGGTTTAG	TACAATTTAT	ATTATCAATG	ATATCAAGTT	ATTGTATGGA	TGTAATTACA	TCAAAAATAT	TAAAAACTTT	AAAGCTTGAA	TATTTAAGAA
**3D7**	**ATAGGTTTAG**	**TACAATTTAT**	**ATTATCAATG**	**ATATCAAGTT**	**ATTGTATGGA**	**TGTAATTACA**	**TCAAAAATAT**	**TAAAAACTTT**	**AAAGCTTGAA**	**TATTTAAGAA**
CBE573_138	ATAGGTTTAG	TACAATTTAT	ATTATCAATG	ATATCAAGTT	ATTGTATGGA	TGTAATTACA	TCAAAAATAT	TAAAAACTTT	AAAGCTTGAA	TATTTAAGAA
CBE576_138	ATAGGTTTAG	TACAATTTAT	ATTATCAATG	ATATCAAGTT	ATTGTATGGA	TGTAATTACA	TCAAAAATAT	TAAAAACTTT	AAAGCTTGAA	TATTTAAGAA
CBE604_138	ATAGGTTTAG	TACAATTTAT	ATTATCAATG	ATATCAAGTT	ATTGTATGGA	TGTAATTACA	TCAAAAATAT	TAAAAACTTT	AAAGCTTGAA	TATTTAAGAA
CBE572_138	ATAGGTTTAG	TACAATTTAT	ATTATCAATG	ATATCAAGTT	ATTGTATGGA	TGTAATTACA	TCAAAAATAT	TAAAAACTTT	AAAGCTTGAA	TATTTAAGAA
CBE569_138	ATAGGTTTAG	TACAATTTAT	ATTATCAATG	ATATCAAGTT	ATTGTATGGA	TGTAATTACA	TCAAAAATAT	TAAAAACTTT	AAAGCTTGAA	TATTTAAGAA
CBE570_138	ATAGGTTTAG	TACAATTTAT	ATTATCAATG	ATATCAAGTT	ATTGTATGGA	TGTAATTACA	TCAAAAATAT	TAAAAACTTT	AAAGCTTGAA	TATTTAAGAA
CBE606_138	ATAGGTTTAG	TACAATTTAT	ATTATCAATG	ATATCAAGTT	ATTGTATGGA	TGTAATTACA	TCAAAAATAT	TAAAAACTTT	AAAGCTTGAA	TATTTAAGAA
CBE581_138	ATAGGTTTAG	TACAATTTAT	ATTATCAATG	ATATCAAGTT	ATTGTATGGA	TGTAATTACA	TCAAAAATAT	TAAAAACTTT	AAAGCTTGAA	TATTTAAGAA

CBE579_138	ATAGGTTTAG	TACAATTTAT	ATTATCAATG	ATATCAAGTT	ATTGTATGGA	TGTAATTACA	TCAAAAATAT	TAAAAACTTT	AAAGCTTGAA	TATTTAAGAA
CBE618_138	ATAGGTTTAG	TACAATTTAT	ATTATCAATG	ATATCAAGTT	ATTGTATGGA	TGTAATTACA	TCAAAAATAT	TAAAAACTAT	AAAGCTTGAA	TATTTAAGAA
CBE614_138	ATAGGTTTAG	TACAATTTAT	ATTATCAATG	ATATCAAGTT	ATTGTATGGA	TGTAATTACA	TCAAAAATAT	TAAAAACTTT	AAAGCTTGAA	TATTTAAGAA
CBE617_138	ATAGGTTTAG	TACAATTTAT	ATTATCAATG	ATATCAAGTT	ATTGTATGGA	TGTAATTACA	TCAAAAATAT	TAAAAACTTT	AAAGCTTGAA	TATTTAAGAA
CBE620_138	ATAGGTTTAG	TACAATTTAT	ATTATCAATG	ATATCAAGTT	ATTGTATGGA	TGTAATTACA	TCAAAAATAT	TAAAATCTTT	AAAGCTTGAA	TATTTAAGAA
CBE597_138	ATAGGTTTAG	TACAATTTAT	ATTATCAATG	ATATCAAGTT	ATTGTATGGA	TGTAATTACA	TCAAAAATAT	TAAAAACTTT	AAAGCTTGAA	TATTTAAGAA
CBE586_138	ATAGGTTTAG	TACAATTTAT	ATTATCAATG	ATATCAAGTT	ATTGTATGGA	TGTAATTACA	TCAAAAATAT	TAAAAACTTT	AAAGCTTGAA	TATTTAAGAA
CBE612_138	ATAGGTTTAG	TACAATTTAT	ATTATCAATG	ATATCAAGTT	ATTGTATGGA	TGTAATTACA	TCAAAAATAT	TAAAAACTTT	AAAGCTTGAA	TATTTAAGAA
CBE583_138	ATAGGTTTAG	TACAATTTAT	ATTATCAATG	ATATCAAGTT	ATTGTATGGA	TGTAATTACA	TCAAAAATAT	TAAAAACTTT	AAAGCTTGAA	TATTTAAGAA
CBE599_138	ATAGGTTTAG	TACAATTTAT	ATTATCAATG	ATATCAAGTT	ATTGTATGGA	TGTAATTACA	TCAGGGATAT	TAAAAACTTT	AAAGCTTGAA	TATTTAAGAA
CBE602_138	ATAGGTTTAG	TACAATTTAT	ATTATCGATG	ATATCGAGTT	ATTGTATGGA	TGTAATTACA	TCAAAAATAT	TAAAAACTTT	AAAGCTTGAA	TATTTAAGAA
CBE607_138	ATAGGTTTAG	TACAATTTAT	ATTATCAATG	ATATCAAGTT	ATTGTATGGA	TGTAATTACA	TCAAAAATAT	TAAAAACTTT	AAAGCTTGAA	TATTTAAGTA
CBE568_138	ATAGGTTTAG	TACAATTTAT	ATTATCAATG	ATATCAAGTT	ATTGTATGGA	TGTAATTACA	TCAAAAATAT	TAAAAACTTT	AAAGCTTGAA	TATTTAAGAA
CBE589_138	ATAGGTTTAG	TACAATTTAT	ATTATCAATG	ATATCAAGTT	ATTGTATGGA	TGTAATTACA	TCAAAAATAT	TAAAAACTTT	AAAGCTTGAA	TATTTAAGAA
CBE605_138	ATAGGTTTAG	TACAATTTAT	ATTATCAATG	ATATCAAGTT	ATTGTATGGA	TGTAATTACA	TCAAAAATAT	TAAAAACTTT	AAAGCTTGAA	TATTTAAGAA
CBE611_138	ATAGGTTTAG	TACAATTTAT	ATTATCAATG	ATATCAAGTT	ATTGTATGGA	TGTAATTACA	TCAAAAATAT	TAAAAACTTT	AAAGCTTGAA	TATTTAAGAA
CBE622_138	ATAGGTTTAG	TACAATTTAT	ATTATCAATG	ATATCAAGTT	ATTGTATGGA	TGTAATTACA	TCAAAAATAT	TAAAAACTTT	AAAGCTTGAA	TATTTAAGAA
CBE585_138	ATAGGTTTAG	TACAATTTAT	ATTATCAATG	ATATCAAGTT	ATTGTATGGA	TGTAATTACA	TCAAAAATAT	TAAAAACTTT	AAAGCTTGAA	TATTTAAGAA
CBE619_138	ATAGGTTTAG	TACAATTTAT	ATTATCAATG	ATATCAAGTT	ATTGTATGGA	TGTAATTACA	TCAAAAATAT	TAAAAACTTT	AAAGCTTGAA	TATTTAAGAA
CBE587_138	ATAGGTTTAG	TACAATTTAT	ATTATCAATG	ATATCAAGTT	ATTGTATGGA	TGTAATTACA	TCAAAAATAT	TAAAAACTTT	AAAGCTTGAA	TATTTAAGAA
CBE648_138	ATAGGTTTAG	TACAATTTAT	ATTATCAATG	ATATCAAGTT	ATTGTATGGA	TGTAATTACA	TCAAAAATAT	TAAAAACTTT	AAAGCTTGAA	TATTTAAGAA
CBE652_138	ATAGGTTTAG	TACAATTTAT	ATTATCAATG	ATATCAAGTT	ATTGTATGGA	TGTAATTACA	TCAAAAATAT	TAAAAACTTT	AAAGCTTGAA	TATTTAAGAA
CBE593_138	ATAGGTTTAG	TACAATTTAT	ATTATCAATG	ATATCAAGTT	ATTGTATGGA	TGTGAATTGA	TCAAAAATAT	TAAAAACTTT	AAAGCTTGAA	TATTTAAGAA
CBE590_138	ATAGGTTTAG	TACAATTTAT	ATTATCAATG	ATATCAAGTT	ATTGTATGGA	TGTAATTACA	TCAAAAATAT	TAAAAACTTT	AAAGCTTGAA	TATTTAAGAA
CBE592_138	ATAGGTTTAG	TACAATTTAT	ATTATCAATG	ATATCAAGTT	ATTGTATGGA	TGTAATTACA	TCAAAAATAT	TAAAAACTTT	AAAGCTTGAA	TATTTAAGAA
CBE601_138	ATAGGTTTAG	TACAATTTAT	ATTATCAATG	ATATCAAGTT	ATTGTATGGA	TGTAATTACA	TCAAAAATAT	TAAAAACTTT	AAAGCTTGAA	TATTTAAGAA
CBE609_138	ATAGGTTTAG	TACAATTTAT	ATTATCAATG	ATATCAAGTT	ATTGTATGGA	TGTAATTACA	TCAAAAATAT	TAAAAACTTT	AAAGCTTGAA	TATTTAAGAA
CBE610_138	ATAGGCTTAG	TACAATTTAT	ATTATCAATG	ATATCAAGTT	ATTGTATGGA	TGTAATTACA	TCAAAAATAT	TAAAAACTTT	AAAGCTTGAA	TATTTAAGAA
CBE616_138	ATAGGTTTAG	TACAATTTAT	ATTATCAATG	ATATCAAGTT	ATTGTATGGA	TGTAATTACA	TCAAAAATAT	TAAAAACTTT	AAAGCTTGAA	TATTTAAGAA
CBE575_138	ATAGGTTTAG	TACAATTTAT	ATTATCAATG	ATATCGAGTT	ATTGTATGGG	TGTAATTACA	TCAGAGATAT	TAAAAACTTT	AAAGCTTGAA	TATTTAAGAA
CBE598_138	ATAGGTTTAG	TACAATTTAT	ATTATCAATG	ATATCAAGTT	ATTGTATGGA	TGTAATTACA	TCAAAAATAT	TAAAAACTTT	AAAGCTTGAA	TATTTAAGAA
CBE608_138	ATAGGTTTAG	TACAATTTAT	ATTATCGATG	ATATCAAGTT	ATTGTATGGA	TGTGATTACA	TCAGAGATAT	TAAAAACTTT	AAAGCTTGAA	TATTTAAGAA
CBE580_138	ATAGGTTTAG	TACAATTTAT	ATTATCAATG	ATATCAAGTT	ATTGTATGGA	TGTGAATTGA	TCAAAAATAT	TAAAAACTTT	AAAGCTTGAA	TATTTAAGAA
CBE649_138	ATAGGTTTAG	TACAATTTAT	ATTATCAATG	ATATCAAGTT	ATTGTATGGA	TGTAATTACA	TCAAAAATAT	TAAAAACTTT	AAAGCTTGAA	TATTTAAGAA
CBE650_138	ATAGGTTTAG	TACAATTTAT	ATTATCAATG	ATATCAAGTT	ATTGTATGGA	TGTAATTACA	TCAAAAATAT	TAAAAACTTT	AAAGCTTGAA	TATTTAAGAA
CBE651_138	ATAGGTTTAG	TACAATTTAT	ATTATCAATG	ATATCAAGTT	ATTGTATGGA	TGTAATTACA	TCAAAAATAT	TAAAAACTTT	AAAGCTTGAA	TATTTAAGAA
CBE584_138	ATAGGTTTAG	TACAATTTAT	ATTATCAATG	ATATCAAGTT	ATTGTATGGA	TGTAATTACA	TCAAAAATAT	TAAAAACTTT	AAAGCTTGAA	TATTTAAGAA
CBE644_138	ATAGGTTTAG	TACAATTTAT	ATTATCAATG	ATATCAAGTT	ATTGTATGGA	TGTAATTACA	TCAAAAATAT	TAAAAACTTT	AAAGCTTGAA	TATTTAAGAA
CBE643_138	ATAGGTTTAG	TACAATTTAT	ATTATCAATG	ATATCAAGTT	ATTGTATGGA	TGTAATTACA	TCAAAAATAT	TAAAAACTTT	AAAGCTTGAA	TATTTAAGAA
CBE582_138	ATAGGTTTAG	TACAATTTAT	ATTATCAATG	ATATCAAGTT	ATTGTATGGA	TGTAATTACA	TCAAAAATAT	TAAAAACTTT	AAAGCTTGAA	TATTTAAGAA
CBE621_138	ATAGGTTTAG	TACAATTTAT	ATTATCAATG	ATATCAAGTT	ATTGTATGGA	TGTAATTACA	TCAAAAATAT	TAAAAACTTT	AAAGCTTGAA	TATTTAAGAA

	401									500
CBE574_138	GTGTTTTTTA	TCAAGATGGA	CAATTTCATG	ATAATAATCC	TGGATCTAAA	TTAAGATCTG	ATTTAGATTT	TTATTTAGAA	CAAGTGAGTT	CAGGAATTGG
CBE577_138	GTGTTTTTTA	TCAAGATGGA	CAATTTCATG	ATAATAATCC	TGGATCTAAA	TTAAGATCTG	ATTTAGATTT	TTATTTAGAA	CAAGTGAGTT	CAGGAATTGG
**3D7**	**GTGTTTTTTA**	**TCAAGATGGA**	**CAATTTCATG**	**ATAATAATCC**	**TGGATCTAAA**	**TTAAGATCTG**	**ATTTAGATTT**	**TTATTTAGAA**	**CAAGTGAGTT**	**CAGGAATTGG**
CBE573_138	GTGTTTTTTA	TCAAGATGGA	CAATTTCATG	ATAATAATCC	TGGATCTAAA	TTAAGATCTG	ATTTAGATTT	TTATTTAGAA	CAAGTGAGTT	CAGGAATTGG
CBE576_138	GTGTTTTTTA	TCAAGATGGA	CAATTTCATG	ATAATAATCC	TGGATCTAAA	TTAAGATCTG	ATTTAGATTT	TTATTTAGAA	CAAGTGAGTT	CAGGAATTGG
CBE604_138	GTGTTTTTTA	TCAAGATGGA	CAATTTCATG	ATAATAATCC	TGGATCTAAA	TTAAGATCTG	ATTTAGATTT	TTATTTAGAA	CAAGTGAGTT	CAGGAATTGG
CBE572_138	GTGTTTTTTA	TCAAGATGGA	CAATTTCATG	ATAATAATCC	TGGATCTAAA	TTAAGATCTG	ATTTAGATTT	TTATTTAGAA	CAAGTGAGTT	CAGGAATTGG
CBE569_138	GTGTTTTTTA	TCAAGATGGA	CAATTTCATG	ATAATAATCC	TGGATCTAAA	TTAAGATCTG	ATTTAGATTT	TTATTTAGAA	CAAGTGAGTT	CAGGAATTGG

```
CBE570_138   GTGTTTTTTA TCAAGATGGA CAATTTCATG ATAATAATCC TGGATCTAAA TTAAGATCTG ATTTAGATTT TTATTTAGAA CAAGTGAGTT CAGGAATTGG
CBE606_138   GTGTTTTTTA TCAAGATGGA CAATTTCATG ATAATAATCC TGGATCTAAA TTAAGATCTG ATTTAGATTT TTATTTAGAA CAAGTGAGTT CAGGAATTGG
CBE581_138   GTGTTTTTTA TCAAGATGGA CAATTTCATG ATAATAATCC TGGATCTAAA TTAAGATCTG ATTTAGATTT TTATTTAGAA CAAGTGAGTT CAGGAATTGG
CBE579_138   GTGTTTTTTA TCAAGATGGA CAATTTCATG ATAATAATCC TGGATCTAAA TTAAGATCTG ATTTAGATTT TTATTTAGAA CAAGTGTATT CAGGAATTGG
CBE618_138   TTGTTTTTTA TCAAGATGGA CAATTTCATG ATAATAATCC TGGATCTAAA TTAAGATCTG ATTTAGATTT TTATTTAGAA CAAGTGAGTT CAGGAATTGG
CBE614_138   GTGTTTTTTA TCAAGATGGA CAATTTCATG ATAATAATCC TGGATCTAAA TTAAGATCTG ATTTAGATTT TTATTTAGAA CAAGTGAGTT CAGGAATTGG
CBE617_138   GTGTTTTTTA TCAAGATGGA CAATTTCATG ATAATAATCC TGGATCTAAA TTAAGATCTG ATTTAGATTT TTATTTAGAA CAAGTGAGTT CAGGAATTGG
CBE620_138   TTGTTTTTTA TCAAGATGGA CAATTTCATG ATAATAATCC TGGATCTAAA TTAAGATCTG ATTTAGATTT TTATTTAGAA CAAGTGAGTT CAGGAATTGG
CBE597_138   GTGTTTTTTA TCAAGATGGA CAATTTCATG ATAATAATCC TGGATCTAAA TTAAGATCTG ATTTAGATTT TTATTTAGAA CAAGTGAGTT CAGGAATTGG
CBE586_138   GTGTTTTTTA TCAAGATGGA CAATTTCATG ATAATAATCC TGGATCTAAA TTAAGATCTG ATTTAGATTT TTATTTAGAA CAAGTGAGTT CAGGAATTGG
CBE612_138   GTGTTTTTTA TCAAGATGGA CAATTTCATG ATAATAATCC TGGATCTAAA TTAAGATCTG ATTTAGATTT TTATTTAGAA CAAGTGAGTT CAGGAATTGG
CBE583_138   GTGTTTTTTA TCAAGATGGA CAATTTCATG ATAATAATCC TGGATCTAAA TTAAGATCTG ATTTAGATTT TTATTTAGAA CAAGTGAGTT CAGGAATTGG
CBE599_138   GTGTTTTTTA TCAAGATGGA CAATTTCATG ATAATAATCC TGGATCTAAA TTAAGATCTG ATTTAGATTT TTATTTAGAA CAAGTGAGTT CAGGAATTGG
CBE602_138   GTGTTTTTTA TCGAGATGGA CAATTTCATG ATAATAATCC TGGATCTAGA TTGAAGACTG ATTTAGATTT TTATTTAGAA CAAGTGAGTT CAGGAATTGG
CBE607_138   GTGTTTTTTA TCTAGATGGA CAATTTCATG ATAATAATCC TGGATCTAAA TTAAGATCTG TATTTGAGTT TTATTTAGAA CAAGTGAGTT CAGGAATTGG
CBE568_138   GTGTTTTTTA TCAAGATGGA CAATTTCATG ATAATAATCC TGGATCTAAA TTAAGATCTG ATTTAGATTT TTATTTAGAA CAAGTGAGTT CAGGAATTGG
CBE589_138   GTGTTTTTTA TCAAGATGGA CAATTTCATG ATAATAATCC TGGATCTAAA TTAAGATCTG ATTTAGATTT TTATTTAGAA CAAGTGAGTT CAGGAATTGG
CBE605_138   GTGTTTTTTA TCAAGATGGA CAATTTCATG ATAATAATCC TGGATCTAAA TTAAGATCTG ATTTAGATTT TTATTTAGAA CAAGTGAGTT CAGGAATTGG
CBE611_138   GTGTTTTTTA TCAAGATGGA CAATTTCATG ATAATAATCC TGGATCTAAA TTAAGATCTG ATTTAGATTT TTATTTAGAA CAAGTGAGTT CAGGAATTGG
CBE622_138   GTGTTTTTTA TCAAGATGGA CAATTTCATG ATAATAATCC TGGATCTAAA TTAAGATCTG ATTTAGATTT TTATTTAGAA CAAGTGAGTT CAGGAATTGG
CBE585_138   GTGTTTTTTA TCAAGATGGA CAATTTCATG ATAATAATCC TGGATCTAAA TTAAGATCTG ATTTAGATTT TTATTTAGAA CAAGTGAGTT CAGGAATTGG
CBE619_138   GTGTTTTTTA TCAAGATGGA CAATTTCATG ATAATAATCC TGGATCTAAA TTAAGATCTG ATTTAGATTT TTATTTAGAA CAAGTGAGTT CAGGAATTGG
CBE587_138   GTGTTTTTTA TCAAGATGGA CAATTTCATG ATAATAATCC TGGATCTAAA TTAAGATCTG ATTTAGATTT TTATTTAGAA CAAGTGAGTT CAGGAATTGG
CBE648_138   GTGTTTTTTA TCAAGATGGA CAATTTCATG ATAATAATCC TGGATCTAAA TTAAGATCTG ATTTAGATTT TTATTTAGAA CAAGTGAGTT CAGGAATTGG
CBE652_138   GTGTTTTTTA TCAAGATGGA CAATTTCATG ATAATAATCC TGGATCTAAA TTAAGATCTG ATTTAGATTT TTATTTAGAA CAAGTGAGTT CAGGAATTGG
CBE593_138   TTGTTTTTTA TCAAGATGGA CAATTTCATG ATAATAATCC TGGATCTAAA TTAAGATCTG ATTTAGATTT TTATTTAGAA CAAGTGAGTT CAGGAATTGG
CBE590_138   GTGTTTTTTA TCAAGATGGA CAATTTCATG ATAATAATCC TGGATCTAAA TTAAGATCTG ATTTAGATTT TTATTTAGAA CAAGTGAGTT CAGGAATTGG
CBE592_138   GTGTTTTTTA TCAAGATGGA CAATTTCATG ATAATAATCC TGGATCTAAA TTAAGATCTG ATTTAGATTT TTATTTAGAA CAAGTGAGTT CAGGAATTGG
CBE601_138   GTGTTTTTTA TCAAGATGGA CAATTTCATG ATAATAATCC TGGATCTAAA TTAAGATCTG ATTTAGATTT TTATTTAGAA CAAGTGAGTT CAGGAATTGG
CBE609_138   GTGTTTTTTA TCAAGATGGA CAATTTCATG ATAATAATCC TGGATCTAAA TTAAGATCTG ATTTAGATTT TTATTTAGAA CAAGTGAGTT CAGGAATTGG
CBE610_138   GTGTTTTTTA TCAAGATGGA CAATTTCATG ATAATAATCC TGGATCTAAA TTAAGATCTG ATTTAGATTT TTATTTAGAA CAAGTGAGTT CTGGAATTGG
CBE616_138   GTGTTTTTTA TCAAGATGGA CAATTTCATG ATAATAATCC TGGATCTAAA TTAAGATCTG ATTTAGATTT TTATTTAGAA CAAGTGAGTT CAGGAATTGG
CBE575_138   TTGTTTTTTA TCAAGATGGA CAATTTCATG ATAATAATCC TGGATCTAAA TTAAGATCTG ATTTAGATTT TTATTTAGAA CAAGTGAGTT CAGGAATTGG
CBE598_138   TTGTTTTTTA TCAAGATGGA CAATTTCATG ATAATAATCC TGGATCTAAA TTAAGATCTG ATTTAGATTT TTATTTAGAA CAAGTGAGTT CAGGAATTGG
CBE608_138   GTGTTTTTTA TCAAGATGGA CAATTTCATG ATAATAATCC TGGATCTAAA TTAAGATCTG ATTTAGATTT TTATTTAGAA CAAGTGAGTT CAGGAATTGG
CBE580_138   TTGTTTTTTA TCAAGATGGA CAATTTCATG ATAATAATCC TGGATCTAAA TTAAGATCTG ATTTAGATTT TTATTTAGAA CAAGTGAGTT CAGGAATTGG
CBE649_138   GTGTTTTTTA TCAAGATGGA CAATTTCATG ATAATAATCC TGGATCTAAA TTAAGATCTG ATTTAGATTT TTATTTAGAA CAAGTGAGTT CAGGAATTGG
CBE650_138   GTGTTTTT-A TCAAGATGGA CAATTTCATG ATAATAATCC TGGATCTAAA TTAAGATCTG ATTTAGATTT TTATTTAGAA CAAGTGAGTT CAGGAATTGG
CBE651_138   GTGTTTTTTA TCAAGATGGA CAATTTCATG ATAATAATCC TGGATCTAAA TTAAGATCTG ATTTAGATTT TTATTTAGAA CAAGTGAGTT CAGGAATTGG
CBE584_138   GTGTTTTTTA TCAAGATGGA CAATTTCATG ATAATAATCC TGGATCTAAA TTAAGATCTG ATTTAGATTT TTATTTAGAA CAAGTGAGTT CAGGAATTGG
CBE644_138   GTGTTTTTTA TCAAGATGGA CAATTTCATG ATAATAATCC TGGATCTAAA TTAAGATCTG ATTTAGATTT TTATTTAGAA CAAGTGAGTT CAGGAATTGG
CBE643_138   GTGTTTTTTA TCAAGATGGA CAATTTCATG ATAATAATCC TGGATCTAAA TTAAGATCTG ATTTAGATTT TTATTTAGAA CAAGTGAGTT CAGGAATTGG
CBE582_138   GTGTTTTTTA TCAAGATGGA CAATTTCATG ATAATAATCC TGGATCTAAA TTAAGATCTG ATTTAGATTT TTATTTAGAA CAAGTGAGTT CAGGAATTGG
CBE621_138   GTGTTTTTTA TCAAGATGGA CAATTTCATG ATAATAATCC TGGATCTAAA TTAAGATCTG ATTTAGATTT TTATTTAGAA CAAGTGAGTT CAGGAATTGG

             501                                                    552                                               600
CBE574_138   TACGAAATTT ATAACAATTT TTACATATGC CAGTTCCTTT TTAGGTTTAT TTATTTGGTC ATTAATAAAA AATGCACGTT TGACTTTATG TATTACT
CBE577_138   TACGAAATTT ATAACAATTT TTACATATGC CAGTTCCTTT TTAGGTTTAT TTATTTGGTC ATTAATAAAA AATGCACGTT TGACTTTATG TATTACT
       3D7   TACGAAATTT ATAACAATTT TTACATATGC CAGTTCCTTT TTAGGTTTAT ATATTTGGTC ATTAATAAAA AATGCACGTT TGACTTTATG TATTACT
CBE573_138   TACGAAATTT ATAACAATTT TTACATATGC CAGTTCCTTT TTAGGTTTAT ATATTTGGTC ATTAATAAAA AATGCACGTT TGACTTTATG TATTACT
CBE576_138   TACGAAATTT ATAACAATTT TTACATATGC CAGTTCCTTT TTAGGTTTAT TTATTTGGTC ATTAATAAAA AATGCACGTT TGACTTTATG TATTACT
```

```
CBE604_138  TACGAAATTT ATAACAATTT TTACATATGC CAGTTCCTTC TTAGGTTTAT TTATTTGGTC ATTAATAAAA AATGCACATT TGACTTTATC GTATAGC
CBE572_138  TACGAAATTT ATAACAATTT TTACATATGC CAGTTCCTTT TTAGGTTTAT TTATTTGGTC ATTAATAAAA AATGCACGTT TGACTTTATG TTTACTT
CBE569_138  TACGAAATTT ATAACAATTT TTACATATGC CAGTTCCTTT TTAGGTTTAT TTATTTGGTC ATTAATAAAA AATGCACGTT TGACTTTATG TATTACT
CBE570_138  TACGAAATTT ATAACAATTT TTACATATGC CAGTTCCTTT TTAGGTTTAT TTATTTGGTC ATTAATAAAA AATGCACGTT TGACTTTATG TAT-ACT
CBE606_138  TACGAAATTT ATAACAATTT TTACATATGC CAGTTCCTTT TTAGGTTTAT ATATTTGGTC ATTAATAAAA AATGCACGTT TGACTTGTAT GTATAAC
CBE581_138  TACGAAATTT ATAACAATTT TTACATATGC CAGTTCCTTC TTAGGTTTAT TTATTTGGTC ATTAATAAAA AATGCACGTT TGACATTTAT GTATTAC
CBE579_138  TACGAAATTT ATAACAATTT TTACATATGC CAGTTCCTTT TTAGGTTTAT TTATTTGGTC ATTAATAAAA AATGCACGTT TGACTTTATG TATTACT
CBE618_138  TACGAAATTT ATAACAATTT TTACATATGC CAGTTCCTTT TTAGGTTTAT ATATTTGGTC ATTAATAAAA AATGCACGTT TGACTTTATC TATGACT
CBE614_138  TACGAAATTT ATAACAATTT TTACATATGC CAGTTCCTTT TTAGGTTTAT ATATTTGGTC ATTAATAAAA AATGCACGTT TGACTTTATG TATTACT
CBE617_138  TACGAAATTT ATAACAATCT ATTGGTATGC CAGTTCCTTT TTAGGTTTAT ATATTTGGTC ATTAATAAAA AATGCACGTT TGACTTTATG TATTACT
CBE620_138  TACGAAATTT ATAACAATAT TTACATATGC CAGTTCCTTT GTAGGTTTAT TTTATTGGTC ATTAATAAAA AATGCACGTT TGACTTTATC TATGACT
CBE597_138  TACGAAATTT ATAACAATTT ATACATATGC CAGTTCCTTT TTAGGTTTAT ATATTTGGTC ATTAATAAAA AATGCACGTT TGACTTTATG TATTACT
CBE586_138  TACGAAATTT ATAACAATCT ATACATATGC CAGTTCCTTT TTAGGTTTAT ATATTTGGTC ATTAATAAAA AATGCACGTT TGACTTTATG TATTACT
CBE612_138  TACGAAATTT ATAACAATTT ATACATATGA CCGTACCTTT TTAGGTTTAT ATATTTGGTC ATTAATAAAA AATGCACGTT TGACTTTATG TATTACT
CBE583_138  TACGAAATTT ATAACGATTT TTACATATGC CAGTTCCTTC TTAGGTTTAT TTATTTGGTC ATTAATAAAA AATGCACGTT TGACTTTATG TATTACC
CBE599_138  TACGAAATTT ATAACAATTT TTACATATGC CAGTTCCTTT TTAGGTTTAT ATATTTGGTC ATTAATAAAA AATGCACGTT TGACTTTATG TATTACT
CBE602_138  TACGAAATTT ATAACAATTT TTACATATGC CAGTTCCTTT TTAGGTTTAT ATATTTGGTC ATTAATAAAA AATGCACGTT TGACTTTATG TATTACT
CBE607_138  TACGAAATTT ATAACAATTT TTACATATGC CAGTTCCTTT TTAGGTTTAT ATATTTGGTC ATTAATAAAA AATGCACGTT TGACTTTATG TATTACT
CBE568_138  TACGAAATTT ATAACAATTT TTACATATGC CAGTTCCTTT TTAGGTTTAT ATATTTGGTC ATTAATAAAA AATGCACGTT TGACTGTATA GTGT
CBE589_138  TACGAAATTT ATAACAATTT TTACATATGC CAGTTCCTTT TTAGGTTTAT ATATTTGGTC ATTAATAAAA AATGCACGTT TGAACTTATT GTATTAA
CBE605_138  TACGAAATTT ATAACAATTT TTACATATGC CAGTTCCTTT TTAGGTTTAT TTATTTGGTC ATTAATAAAA AATGCACGTT TGACTTTATG TTATTAA
CBE611_138  TACGAAATTT ATAACAATTT TTACATATGC CAGTTCCTTT TTAGGTTTAT TTATTTGGTC ATTAATAAAA AATGCACGAT TGA
CBE622_138  TACGAAATTT ATAACAATTT TTACATATGC CAGTTCCTTT TTAGGTTTAT TTATTTGGTC ATTAATAAAA AATGCCTATT T
CBE585_138  TACGAAATTT ATAACAATTT TTACATATGC CAGTTCCTTT TTAGGTTTAT TTATTTGGTC ATTAATAAAA AATGCACTAT TTGTA
CBE619_138  TACGAAATTT ATAACAATTT TTACATATGC CAGTTCCTTT TTAGGTTTAT TTATTTGGTC ATTAATAAAA AATGCATTAT TTGT
CBE587_138  TACGAAATTT ATAACAATTT TTACATATGC CAGTTCCTTT TTAGGTTTAT ATATTAGGTC ATTAATAAAA AATGCATTTT TG
CBE648_138  TACGAAATTT ATAACAATTT TTACATATGC CAGTTCCTTT TTAGGTTTAT TTATTTGGTC ATTAATAAAA AATGCACGTT TGACTTTATG TATTACT
CBE652_138  TACGAAATTT ATAACAATTT TTACATATGC CAGTTCCTTT TTAGGTTTAT TTATTTGGTC ATTAATAAAA AATGCACGTT TGACTTTATG TATTACT
CBE593_138  TACGAAATTT ATAACAATTT TTACATATGC CAGTTCCTTT TTAGGTTTAT TTATTTGGTC ATTAATAAAA AATGCACGTT TGACTTTATG TATTACT
CBE590_138  TACGAAATTT ATAACAATTT TTACATATGC CAGTTCCTTT TTAGGTTTAT TTATTTGGTC ATTTATAAAA
CBE592_138  TACGAAATTT ATAACGATTT TTACATATGC CAGTTCCTTT TTAGGTTTAT TTATTTGGTC ATTAATAAAA G
CBE601_138  TACGAAATTT ATAACGATTT TTACATATGC CAGTTCCTTT TTAGGTTTAT TTATTTGGTC ATTAATAAAA G
CBE609_138  TACGAAATTT ATAACAATTT TTACATATGC CAGTTCCTTT TTAGGTTTAT TTATTTGGTC ATTAAATAAA
CBE610_138  TACGAAATTT ATAACGATTT TTACATATGC CGGTTCCTTT TTAGGTTTAT ATATTTGGTC ATTAATA
CBE616_138  TACGAAATTT ATAACGATTT TTACATATGC CAGTTCCTTT TTAGGTTTAT TTATTTGGTC ATTTTTGA
CBE575_138  TACGAAATTT ATAACAATTT TTACATATGC CAGTTCCTTT TTAGGTTTAT ATATTTGGTC ATTAATAAAA AATGCACGTT TGACTTTATG TATTACT
CBE598_138  TACGAAATTT ATAACAATTT TTACATATGC CAGTTCCTTT TTAGGTTTAT ATATTTGGTC ATTAATAAAA AATGCACGTT TGACTTTATG TATTACT
CBE608_138  TACGAAATTT ATAACAATTT TTACATATGC CAGTTCCTTT TTAGGTTTAT ATATTTGGTC ATTAATAAAA AATGCACGTT TGACTTTATG TATTACT
CBE580_138  TACGAAATTT ATAACAATTT TTACATATGC CAGTTCCTTT TTAGGTTTAT ATATTTGGTC ATTAATAAAA AATGCACGTT TGACTTTATG TATTACT
CBE649_138  TACGAAATTT ATAACAATTT TTACATATGC CAGTTCCTTT TTAGGTTTAT TTATTTGGTC ATTAATAAAA AATGCACGTT TGACTTTATG TATTACT
CBE650_138  TACGAAATTT ATAACAATTT TTACATATGC CAGTTCCTTT TTAGGTTTAT TTATTTGGTC ATTAATAAAA AATGCACGTT TGACTTTATG TATTACT
CBE651_138  TACGAAATTT ATAACAATTT TTACATATGC CAGTTCCTTT TTAGGTTTAT TTATTTGGTC ATTAATAAAA AATGCACGTT TGACTTTATG TATTACT
CBE584_138  TACGAAATTT ATAACAATTT TTACATATGC CAGTTCCTTT TTAGGTTTAT TTATTTGGTC ATTAATAAAA AATGCACGTT TGACTTTATG TATTACC
CBE644_138  TACGAAATTT ATAACAATTT TTACATATGC CAGTTCCTTT TTAGGTTTAT ATATTTGGTC ATTAATAAAA AATGCACGTT TGACTTTATG TATTACT
CBE643_138  TACGAAATTT ATAACAATTT TTACATATGC CAGTTCCTTT TTAGGTTTAT TTATTTGGTC ATTAATAAAA AATGCACGTT TGACTTTATG TATTACT
CBE582_138  TACGAAATTT ATAACAATTT TTACATATGC CAGTTTTTGA
CBE621_138  TACGAAATTT ATAACAATTT TTACATATGC CAGTTCCTTT TTAGGTTTAT ATATTTGGTC ATTAATAAAA AATGCACGT T TGACTTTTA TTGTAAT
```

Samples from 2016, gene *pfmdr1* D1246Y

	3701			3738						3800
CBE695_138	AGATGATATT	AATATATGTG	ATTATAACTT	AAGAGATCTT	AGAAACTTAT	TTTCAATAGT	TAGTCAAGAA	CCCATGTTAT	TTAATATGTC	CATATATGAA
CBE682_138	AGATGATATT	AATATATGTG	ATTATAACTT	AAGAGATCTT	AGAAACTTAT	TTTCAATAGT	TAGTCAAGAA	CCCATGTTAT	TTAATATGTC	CATATATGAA
CBE686_138	AGATGATATT	AATATATGTG	ATTATAACTT	AAGAGATCTT	AGAAACTTAT	TTTCAATAGT	TAGTCAAGAA	CCCATGTTAT	TTAATATGTC	CATATATGAA
CBE688_138	AGATGATATT	AATATATGTG	ATTATAACTT	AAGAGATCTT	AGAAACTTAT	TTTCAATAGT	TAGTCAAGAA	CCCATGTTAT	TTAATATGTC	CATATATGAA
CBE689_138	AGATGATATT	AATATATGTG	ATTATAACTT	AAGAGATCTT	AGAAACTTAT	TTTCAATAGT	TAGTCAAGAA	CCCATGTTAT	TTAATATGTC	CATATATGAA
**3D7**	**AGATGATATT**	**AATATATGTG**	**ATTATAACTT**	**AAGAGATCTT**	**AGAAACTTAT**	**TTTCAATAGT**	**TAGTCAAGAA**	**CCCATGTTAT**	**TTAATATGTC**	**CATATATGAA**
CBE690_138	AGATGATATT	AATATATGTG	ATTATAACTT	AAGAGATCTT	AGAAACTTAT	TTTCAATAGT	TAGTCAAGAA	CCCATGTTAT	TTAATATGTC	CATATATGAA
CBE694_138	AGATGATATT	AATATATGTG	ATTATAACTT	AAGAGATCTT	AGAAACTTAT	TTTCAATAGT	TAGTCAAGAA	CCCATGTTAT	TTAATATGTC	CATATATGAA
CBE701_138	AGATGATATT	AATATATGTG	ATTATAACTT	AAGAGATCTT	AGAAACTTAT	TTTCAATAGT	TAGTCAAGAA	CCCATGTTAT	TTAATATGTC	CATATATGAA
CBE681_138	AGATGATATT	AATATATGTG	ATTATAACTT	AAGAGATCTT	AGAAACTTAT	TTTCAATAGT	TAGTCAAGAA	CCCATGTTAT	TTAATATGTC	CATATATGAA
CBE698_138	AGATGATATT	AATATATGTG	ATTATAACTT	AAGAGATCTT	AGAAACTTAT	TTTCAATAGT	TAGTCAAGAA	CCCATGTTAT	TTAATATGTC	CATATATGAA
CBE684_138	AGATGATATT	AATATATGTG	ATTATAACTT	AAGAGATCTT	AGAAACTTAT	TTTCAATAGT	TAGTCAAGAA	CCCATGTTAT	TTAATATGTC	CATATATGAA
CBE702_138	AGATGATATT	AATATATGTG	ATTATAACTT	AAGAGATCTT	AGAAACTTAT	TTTCAATAGT	TAGTCAAGAA	CCCATGTTAT	TTAATATGTC	CATATATGAA
CBE697_138	AGATGATATT	AATATATGTG	ATTATAACTT	AAGAGATCTT	AGAAACTTAT	TTTCAATAGT	TAGTCAAGAA	CCCATGTTAT	TTAATATGTC	CATATATGAA
CBE699_138	AGATGATATT	AATATATGTG	ATTATAACTT	AAGAGATCTT	AGAAACTTAT	TTTCAATAGT	TAGTCAAGAA	CCCATGTTAT	TTAATATGTC	CATATATGAA
CBE704_138	AGATGATATT	AATATATGTG	ATTATAACTT	AAGAGATCTT	AGAAACTTAT	TTTCAATAGT	TAGTCAAGAA	CCCATGTTAT	TTAATATGTC	CATATATGAA
CBE687_138	AGATGATATT	AATATATGTG	ATTATAACTT	AAGAGATCTT	AGAAACTTAT	TTTCAATAGT	TAGTCAAGAA	CCCATGTTAT	TTAATATGTC	CATATATGAA
CBE705_138	AGATGATATT	AATATATGTG	ATTATAACTT	AAGAGATCTT	AGAAACTTAT	TTTCAATAGT	TAGTCAAGAA	CCCATGTTAT	TTAATATGTC	CATATATGAA
CBE685_138	AGATGATATT	AATATATGTG	ATTATAACTT	AAGAGATCTT	AGAAACTTAT	TTTCAATAGT	TAGTCAAGAA	CCCATGTTAT	TTAATATGTC	CATATATGAA
CBE706_138	AGATGATATT	AATATATGTG	ATTATAACTT	AAGAGATCTT	AGAAACTTAT	TTTCAATAGT	TAGTCAAGAA	CCCATGTTAT	TTAATATGTC	CATATATGAA
CBE693_138	GATATT	AATATATGTG	ATTATAACTT	AAGAGATCTT	AGAAACTTAT	TTTCAATAGT	TAGTCAAGAA	CCCATGTTAT	TTAATATGTC	CATATATGAA
CBE707_138		ATGTG	ATTATAACTT	AAGAGATCTT	AGAAACTTAT	TTTCAATAGT	TAGTCAAGAA	CCCATGTTAT	TTAATATGTC	CATATATGAA
CBE691_138		TGTG	ATTATAACTT	AAGAGATCTT	AGAAACTTAT	TTTCAATAGT	TAGTCAAGAA	CCCATGTTAT	TTAATATGTC	CATATATGAA
CBE683_138		TGTG	ATTATAACTT	AAGAGATCTT	AGAAACTTAT	TTTCAATAGT	TAGTCAAGAA	CCCATGTTAT	TTAATATGTC	CATATATGAA

	3801									3900
CBE695_138	AATATCAAAT	TTGGAAGAGA	AGATGCAACA	TTGGAAGATG	TTAAACGTGT	TAGTAAGTTT	TGCTGCTATA	GATGAATTTA	TCGAATCATT	ACCAAATAAA
CBE682_138	AATATCAAAT	TTGGAAGAGA	AGATGCAACA	TTGGAAGATG	TTAAACGTGT	TAGTAAGTTT	-GCTGCTATA	GATGAATTTA	TCGAATCATT	ACCAAATAAA
CBE686_138	AATATCAAAT	TTGGAAGAGA	AGATGCAACA	TTGGAAGATG	TTAAACGTGT	TAGTAAGTTT	-GCTGCTATA	GATGAATTTA	TCGAATCATT	ACCAAATAAA
CBE688_138	AATATCAAAT	TTGGAAGAGA	AGATGCAACA	TTGGAAGATG	TTAAACGTGT	TAGTAAGTTT	-GCTGCTATA	GATGAATTTA	TCGAATCATT	ACCAAATAAA
CBE689_138	AATATCAAAT	TTGGAAGAGA	AGATGCAACA	TTGGAAGATG	TTAAACGTGT	TAGTAAGTTT	-GCTGCTATA	GATGAATTTA	TCGAATCATT	ACCAAATAAA
3D7	AATATCAAAT	TTGGAAGAGA	AGATGCAACA	TTGGAAGATG	TTAAACGTGT	TAGTAAGTTT	-GCTGCTATA	GATGAATTTA	TCGAATCATT	ACCAAATAAA
CBE690_138	AATATCAAAT	TTGGAAGAGA	AGATGCAACA	TTGGAAGATG	TTAAACGTGT	TAGTAAGTTT	-GCTGCTATA	GATGAATTTA	TCGAATCATT	ACCAAATAAA
CBE694_138	AATATCAAAT	TTGGAAGAGA	AGATGCAACA	TTGGAAGATG	TTAAACGTGT	TAGTAAGTTT	-GCTGCTATA	GATGAATTTA	TCGAATCATT	ACCAAATAAA
CBE701_138	AATATCAAAT	TTGGAAGAGA	AGATGCAACA	TTGGAAGATG	TTAAACGTGT	TAGTAAGTTT	-GCTGCTATA	GATGAATTTA	TCGAATCATT	ACCAAATAAA
CBE681_138	AATATCAAAT	TTGGAAGAGA	AGATGCAACA	TTGGAAGATG	TTAAACGTGT	TAGTAAGTTT	-GCTGCTATA	GATGAATTTA	TCGAATCATT	ACCAAATAAA
CBE698_138	AATATCAAAT	TTGGAAGAGA	AGATGCAACA	TTGGAAGATG	TTAAACGTGT	TAGTAAGTTT	-GCTGCTATA	GATGAATTTA	TCGAATCATT	ACCAAATAAA
CBE684_138	AATATCAAAT	TTGGAAGAGA	AGATGCAACA	TTGGAAGATG	TTAAACGTGT	TAGTAAGTTT	-GCTGCTATA	GATGAATTTA	TCGAATCATT	ACCAAATAAA
CBE702_138	AATATCAAAT	TTGGAAGAGA	AGATGCAACA	TTGGAAGATG	TTAAACGTGT	TAGTAAGTTT	-GCTGCTATA	GATGAATTTA	TCGAATCATT	ACCAAATAAA
CBE697_138	AATATCAAAT	TTGGAAGAGA	AGATGCAACA	TTGGAAGATG	TTAAACGTGT	TAGTAAGTTT	-GCTGCTATA	GATGAATTTA	TCGAATCATT	ACCAAATAAA
CBE699_138	AATATCAAAT	TTGGAAGAGA	AGATGCAACA	TTGGAAGATG	TTAAACGTGT	TAGTAAGTTT	-GCTGCTATA	GATGAATTTA	TCGAATCATT	ACCAAATAAA
CBE704_138	AATATCAAAT	TTGGAAGAGA	AGATGCAACA	TTGGAAGATG	TTAAACGTGT	TAGTAAGTTT	-GCTGCTATA	GATGAATTTA	TCGAATCATT	ACCAAATAAA

```
CBE687_138  AATATCAAAT TTGGAAGAGA AGATGCAACA TTGGAAGATG TTAAACGTGT TAGTAAGTTT -GCTGCTATA GATGAATTTA TCGAATCATT ACCAAATAAA
CBE705_138  AATATCAAAT TTGGAAGAGA AGATGCAACA TTGGAAGATG TTAAACGTGT TAGTAAGTTT -GCTGCTATA GATGAATTTA TCGAATCATT ACCAAATAAA
CBE685_138  AATATCAAAT TTGGAAGAGA AGATGCAACA TTGGAAGATG TTAAACGTGT TAGTAAGTTT -GCTGCTATA GATGAATTTA TCGAATCATT ACCAAATAAA
CBE706_138  AATATCAAAT TTGGAAGAGA AGATGCAACA TTGGAAGATG TTAAACGTGT TAGTAAGTTT -GCTGCTATA GATGAATTTA TCGAATCATT ACCAAATAAA
CBE693_138  AATATCAAAT TTGGAAGAGA AGATGCAACA TTGGAAGATG TTAAACGTGT TAGTAAGTTT -GCTGCTATA GATGAATTTA TCGAATCATT ACCAAATAAA
CBE707_138  AATATCAAAT TTGGAAGAGA AGATGCAACA TTGGAAGATG TTAAACGTGT TAGTAAGTTT -GCTGCTATA GATGAATTTA TCGAATCATT ACCAAATAAA
CBE691_138  AATATCAAAT TTGGAAGAGA AGATGCAACA TTGGAAGATG TTAAACGTGT TAGTAAGTTT -GCTGCTATA GATGAATTTA TCGAATCATT ACCAAATAAA
CBE683_138  AATATCAAAT TTGGAAGAGA AGATGCAACA TTGGAAGATG TTAAACGTGT TAGTAAGTTT -GCTGCTATA GATGAATTTA TCGAATCATT ACCAAATAAA
```

Samples from 2017, gene *pfmdr1* N86Y and Y184F

```
            201                                                        256                                               300
CBE541_138  AGGAGGAACA TTACCTTTTT TTATATCTGT GTTTGGTGTA ATATTAAAGA ACATGTATTT AGGTGATGAT ATTAATCCTA TAATATTATC ATTAGTATCT
CBE565_138  AGGAGGAACA TTACCTTTTT TTATATCTGT GTTTGGTGTA ATATTAAAGA ACATGTATTT AGGTGATGAT ATTAATCCTA TAATATTATC ATTAGTATCT
CBE559_138  AGGAGGAACA TTACCTTTTT TTATATCTGT GTTTGGTGTA ATATTAAAGA ACATGAATTT AGGTGATGAT ATTAATCCTA TAATATTGTC ATTAGTATCT
CBE558_138  AGGAGGAACA TTACCTTTTT TTATATCTGT GTTTGGTGTA ATATTAAAGA ACATGAATTT AGGTGATGAT ATTAATCCTA TAATATTATC ATTAGTATCT
CBE555_138  AGGAGGAACA TTACCTTTTT TTATATCTGT GTTTGGTGTA ATATTAAAGA ACATGAATTT AGGTGATGAT ATTAATCCTA TAATATTATC ATTAGTATCT
CBE550_138  AGGAGGAACA TTACCTTTTT TTATATCTGT GTTTGGTGTA ATATTAAAGA ACATGAATTT AGGTGATGAT ATTAATCCTA TAATATTATC ATTAGTATCT
CBE551_138  AGGAGGAACA TTACCTTTTT TTATATCTGT GTTTGGTGTA ATATTAAAGA ACATGTATTT AGGTGATGAT ATTAATCCTA TAATATTATC ATTAGTATCT
CBE546_138  AGGAGGAACA TTACCTTTTT TTATATCTGT GTTTGGTGTA ATATTAAAGA ACATGAATTT AGGTGATGAT ATTAATCCTA TAATATTATC ATTAGTATCT
CBE564_138  AGGAGGAACA TTACCTTTTT TTATATCTGT GTTTGGTGTA ATATTAAAGA ACATGTAATTT AGGTGATGAT ATTAATCCTA TAATATTATC ATTAGTATCT
CBE549_138  AGGAGGAACA TTACCTTTTT TTATATCTGT GTTTGGTGTA ATATTAAAGA ACATGAATTT AGGTGATGAT ATTAATCCTA TAATATTATC ATTAGTATCT
CBE653_138  AGGAGGAACA TTACCTTTTT TTATATCTGT GTTTGGTGTA ATATTAAAGA ACATGAATTT AGGTGATGAT ATTAATCCTA TAATATTATC ATTAGTATCT
CBE542_138  AGGAGGAACA TTACCTTTTT TTATATCTGT GTTTGGTGTA ATATTAAAGA ACATGTATTT AGGTGATGAT ATTAATCCTA TAATATTATC ATTAGTATCT
CBE556_138  AGGAGGAACA TTACCTTTTT TTATATCTGT GTTTGGTGTA ATATTAAAGA ACATGAATTT AGGTGATGAT ATTAATCCTA TAATATTATC ATTAGTATCT
       3D7  AGGAGGAACA TTACCTTTTT TTATATCTGT GTTTGGTGTA ATATTAAAGA ACATGAATTT AGGTGATGAT ATTAATCCTA TAATATTATC ATTAGTATCT
CBE540_138  AGGAGGAACA TTACCTTTTT TTATATCTGT GTTTGGTGTA ATATTAAAGA ACATGAATTT AGGTGATGAT ATTAATCCTA TAATATTATC ATTAGTATCT
CBE543_138  AGGAGGAACA TTACCTTTTT TTATATCTGT GTTTGGTGTA ATATTAAAGA ACATGAATTT AGGTGATGAT ATTAATCCTA TAATATTATC ATTAGTATCT
CBE554_138  AGGAGGAACA TTACCTTTTT TTATATCTGT GTTTGGTGTA ATATTAAAGA ACATGAATTT AGGTGATGAT ATTAATCCTA TAATATTATC ATTAGTATCT
CBE563_138  AGGAGGAACA TTACCTTTTT TTATATCTGT GTTTGGTGTA ATATTAAAGA ACATGAATTT AGGTGATGAT ATTAATCCTA TAATATTATC ATTAGTATCT
CBE547_138  AGGAGGAACA TTACCTTTTT TTATATCTGT GTTTGGTGTA ATATTAAAGA ACATGAATTT AGGTGATGAT ATTAATCCTA TAATATTATC ATTAGTATCT
CBE567_138  AGGAGGAACA TTACCTTTTT TTATATCTGT GTTTGGTGTA ATATTAAAGA ACATGAATTT AGGTGATGAT ATTAATCCTA TAATATTATC ATTAGTATCT
CBE557_138  AGGAGGAACA TTACCTTTTT TTATATCTGT GTTTGGTGTA ATATTAAAGA ACATGAATTT AGGTGATGAT ATTAATCCTA TAATATTATC ATTAGTATCT
CBE560_138                                 T GTTTGGTGTA ATATTAAAGA ACATGAATTT AGGTGATGAT ATTAATCCTA TAATATTATC ATTAGTATCT
CBE552_138                                       GGTGTA ATATTAAAGA ACATGAATTT AGGTGATGAT ATTAATCCTA TAATATTATC ATTAGTATCT
CBE562_138                                       GGTGTA ATATTAAAGA ACATGAATTT AGGTGATGAT ATTAATCCTA TAATATTATC ATTAGTATCT
CBE553_138                                            A ATATTAAAGA ACATGAATTT AGGTGATGAT ATTAATCCTA TAATATTATC ATTAGTATCT
CBE545_138                                          GTA ATATTAAAGA ACATGAATTT AGGTGATGAT ATTAATCCTA TAATATTATC ATTAGTATCT

            301                                                                                                       400
CBE541_138  ATAGGTTTAG TACAATTTAT ATTATCAATG ATATCAAGTT ATTGTATGGA TGTAATTACA TCAAAAATAT TAAAAACTTT AAAGCTTGAA TATTTAAGAA
CBE565_138  ATAGGTTTAG TACAATTTAT ATTATCAATG ATATCAAGTT ATTGTATGGA TGTAATTACA TCAAAAATAT TAAAAACTTT AAAGCTTGAA TATTTAAGAA
CBE559_138  ATAGGTTTAG TACAATTTAT ATTATCAATG ATATCAAGTT ATTGTATGGA TGTAATTACA TCAAAAATAT TAAAAACTTT AAAGCTTGAA TATTTAAGAA
CBE558_138  ATAGGTTTAG TACAATTTAT ATTATCAATG ATATCAAGTT ATTGTATGGA TGTAATTACA TCAAAAATAT TAAAAACTTT AAAGCTTGAA TATTTAAGAA
```

```
CBE555_138  ATAGGTTTAG TACAATTTAT ATTATCAATG ATATCAAGTT ATTGTATGGA TGTAATTACA TCAAAAATAT TAAAAACTTT AAAGCTTGAA TATTTAAGAA
CBE550_138  ATAGGTTTAG TACAATTTAT ATTATCAATG ATATCAAGTT ATTGTATGGA TGTAATTACA TCAAAAATAT TAAAAACTTT AAAGCTTGAA TATTTAAGAA
CBE551_138  ATAGGTTTAG TACAATTTAT ATTATCAATG ATATCAAGTT ATTGTATGGA TGTAATTACA TCAAAAATAT TAAAAACTTT AAAGCTTGAA TATTTAAGAA
CBE546_138  ATAGGTTTAG TACAATTTAT ATTATCAATG ATATCAAGTT ATTGTATGGA TGTAATTACA TCAAAAATAT TAAAAACTTT AAAGCTTGAA TATTTAAGAA
CBE564_138  ATAGGTTTAG TACAATTTAT ATTATCAATG ATATCAAGTT ATTGTATGGA TGTAATTACA TCAAAAATAT TAAAAACTTT AAAGCTTGAA TATTTAAGAA
CBE548_138  ATAGGCTTAG TACAATTTAT ATTATCAATG ATATCAAGTT ATTGTATGGA TGTAATTACA TCAAAAATAT TAAAAACTTT AAAGCTTGAA TATTTAAGAA
CBE549_138  ATAGGTTTAG TACAATTTAT ATTATCAATG ATATCAAGTT ATTGTATGGA TGTAATTACA TCAAAAATAT TAAAAACTTT AAAGCTTGAA TATTTAAGAA
CBE653_138  ATAGGTTTAG TACAATTTAT ATTATCAATG ATATCAAGTT ATTGTATGGA TGTAATTACA TCAAAAATAT TAAAAACTTT AAAGCTTGAA TATTTAAGAA
CBE542_138  ATAGGTTTAG TACAATTTAT ATTATCTATG ATATCAAGTT ATTGTATGGA TGTAATTACA TCGAAGATAT TAAAAACTTT AAAGCTTGAA TATTTAAGAA
CBE556_138  ATAGGTTTAG TACAATTTAT ATTATCAATG ATATCAAGTT ATTGTATGGA TGTAATTACA TCAAAAATAT TAAAAACTTT AAAGCTTGAA TATTTAAGAA
       3D7  ATAGGTTTAG TACAATTTAT ATTATCAATG ATATCAAGTT ATTGTATGGA TGTAATTACA TCAAAAATAT TAAAAACTTT AAAGCTTGAA TATTTAAGAA
CBE540_138  ATAGGTTTAG TACAATTTAT ATTATCAATG ATATCAAGTT ATTGTATGGA TGTAATTACA TCAAAAATAT TAAAAACTTT AAAGCTTGAA TATTTAAGAA
CBE543_138  ATAGGTTTAG TACAATTTAT ATTATCAATG ATATCAAGTT ATTGTATGGA TGTAATTACA TCGAAAATAT TAAAAACTTT AAAGCTTGAA TATTTAAGAA
CBE554_138  ATAGGTTTAG TACAATTTAT ATTATCGATG ATATCGAGTT ATTGTATGGA TGTGATTACA TCAAAAATAT TAAAAACTTT AAAGCTTGAA TATTTAAGAA
CBE563_138  ATAGGTTTAG TACGATTTAT ATTATCGATG ATATCAAGTT ATTGTATGGG TGTAATTACA TCGAAGATAT TAAAAACTTT AAAGCTTGAA TATTTAAGAA
CBE547_138  ATAGGTTTAG TACAATTTAT ATTATCAATG ATATCAAGTT ATTGTATGGA TGTAATTACA TCAAAAATAT TAAAAACTTT AAAGCTTGAA TATTTAAGAA
CBE567_138  ATAGGTTTAG TACAATTTAT ATTATCAATG ATATCAAGTT ATTGTATGGA TGTAATTACA TCAAAAATAT TAAAAACTTT AAAGCTTGAA TATTTAAGAA
CBE557_138  ATAGGTTTAG TACAATTTAT ATTATCAATG ATATCAAGTT ATTGTATGGA TGTAATTACA TCAAAAATAT TAAAAACTTT AAAGCTTGAA TATTTAAGAA
CBE560_138  ATAGGTTTAG TACAATTTAT ATTATCAATG ATATCAAGTT ATTGTATGGA TGTAATTACA TCAAAAATAT TAAAAACTTT AAAGCTTGAA TATTTAAGAA
CBE552_138  ATAGGTTTAG TACAATTTAT ATTATCAATG ATATCAAGTT ATTGTATGGA TGTAATTACA TCAAAAATAT TAAAAACTTT AAAGCTTGAA TATTTAAGAA
CBE562_138  ATAGGTTTAG TACAATTTAT ATTATCAATG ATATCAAGTT ATTGTATGGA TGTAATTACA TCAAAAATAT TAAAAACTTT AAAGCTTGAA TATTTAAGAA
CBE553_138  ATAGGTTTAG TACAATTTAT ATTATCAATG ATATCAAGTT ATTGTATGGA TGTAATTACA TCAAAAATAT TAAAAACTTT AAAGCTTGAA TATTTAAGAA
CBE545_138  ATAGGTTTAG TACAATTTAT ATTATCAATG ATATCAAGTT ATTGTATGGA TGTAATTACA TCAAAAATAT TAAAAACTTT AAAGCTTGAA TATTTAAGAA

            401                                                                                                      500
CBE541_138  GTGTTTTTTA TCAAGATGGA CAATTTCATG ATAATAATCC TGGATCTAAA TTAAGATCTG ATTTAGATTT TTATTTAGAA CAAGTGAGTT CAGGAATTGG
CBE565_138  GTGTTTTTTA TCAAGATGGA CAATTTCATG ATAATAATCC TGGATCTAAA TTAAGATCTG ATTTAGATTT TTATTTAGAA CAGTGAGTTT CAGGAATTGG
CBE559_138  GTGTTTTTTA TCAAGATGGA CAATTTCATG ATAATAATCC TGGATCTAAA TTAAGATCTG ATTTAGATTT TTATTTAGAA CAAGTGAGTT CAGGAATTGG
CBE558_138  GTGTTTTTTA TCAAGATGGA CAATTTCATG ATAATAATCC TGGATCTAAA TTAAGATCTG ATTTAGATTT TTATTTAGAA CAAGTGAGTT CAGGAATTGG
CBE555_138  GTGTTTTTTA TCAAGATGGA CAATTTCATG ATAATAATCC TGGATCTAAA TTAAGATCTG ATTTAGATTT TTATTTAGAA CAAGTGAGTT CAGGAATTGG
CBE550_138  GTGTTTTTTA TCAAGATGGA CAATTTCATG ATAATAATCC TGGATCTAAA TTAAGATCTG ATTTAGATTT TTATTTAGAA CAAGTGAGTT CAGGAATTGG
CBE551_138  GTGTTTTTTA TCAAGATGGA CAATTTCATG ATAATAATCC TGGATCTAAA TTAAGATCTG ATTTAGATTT TTATTTAGAA CAAGTGAGTT CAGGAATTGG
CBE546_138  GTGTTTTTTA TCAAGATGGA CAATTTCATG ATAATAATCC TGGATCTAAA TTAAGATCTG ATTTAGATTT TTATTTAGAA CAAGTGAGTT CAGGAATTGG
CBE564_138  GTGTTTTTTA TCAAGATGGA CAATTTCATG ATAATAATCC TGGATCTAAA TTAAGATCTG ATTTAGATTT TTATTTAGAA CAAGTGAGTT CAGGAATTGG
CBE548_138  GTGTTTTTTA TCAAGATGGA CAATTTCATG ATAATAATCC TGGATCTAAA TTAAGATCTG ATTTAGATTT TTATTTAGAA CAAGTGAGTT CAGGAATTGG
CBE549_138  GTGTTTTTTA TCAAGATGGA CAATTTCATG ATAATAATCC TGGATCTAAA TTAAGATCTG ATTTAGATTT TTATTTAGAA CAAGTGAGTT CAGGAATTGG
CBE653_138  GTGTTTTTTA TCAAGATGGA CAATTTCATG ATAATAATCC TGGATCTAAA TTAAGATCTG ATTTAGATTT TTATTTAGAA CAAGTGAGTT CAGGAATTGG
CBE542_138  GTGTTTTTTA TCAAGATGGA CAATTTCATG ATAATAATCC TGGATCTAAA TTAAGATCTG ATTTAGATTT TTATTTAGAA CAAGTGAGTT CAGGAATTGG
CBE556_138  GTGTTTTTTA TCAAGATGGA CAATTTCATG ATAATAATCC TGGATCTAAA TTAAGATCTG ATTTAGATTT TTATTTAGAA CAAGTGAGTT CAGGAATTGG
       3D7  GTGTTTTTTA TCAAGATGGA CAATTTCATG ATAATAATCC TGGATCTAAA TTAAGATCTG ATTTAGATTT TTATTTAGAA CAAGTGAGTT CAGGAATTGG
CBE540_138  GTGTTTTTTA TCAAGATGGA CAATTTCATG ATAATAATCC TGGATCTAAA TTAAGATCTG ATTTAGATTT TTATTTAGAA CAAGTGAGTT CAGGAATTGG
CBE543_138  GTGTTTTTTA TCAAGATGGA CAATTTCATG ATAATAATCC TGGATCTAAA TTAAGATCTG ATTTAGATTT TTATTTAGAA CAAGTGAGTT CAGGAATTGG
CBE554_138  GTGTTTTTTA TCAAGATGGA CAATTTCATG ATAATAATCC TGGATCTAAA TTAAGGGGTG ATTTAGATTT TTATTTAGAA CAAGTGAGTT CAGGAATTGG
CBE563_138  GTGTTTTTTA TCAAGATGGA CAATTTCATG ATAATAATCC TGGATCTAAA TTAAGATCTG ATTTAGATTT TTATTTAGAA CAAGTGAGTT CAGGAATTGG
CBE547_138  GTGTTTTTTA TCAAGATGGA CAATTTCATG ATAATAATCC TGGATCTAAA TTAAGATCTG ATTTAGATTT TTATTTAGAA CAAGTGAGTT CAGGAATTGG
CBE567_138  GTGTTTTTTA TCAAGATGGA CAATTTCATG ATAATAATCC TGGATCTAAA TTAAGATCTG ATTTAGATTT TTATTTAGAA CAAGTGAGTT CAGGAATTGG
```

```
CBE557_138   GTGTTTTTTA TCGAGATGGA CAATTTCATG ATAATAATCC TGGATCTAAA TTAAGATCTG ATTTAGATTT TTATTTAGAA CAAGTGAGTT CAGGAATTGG
CBE560_138   GTGTTTTTTA TCAAGATGGA CAATTTCATG ATAATAATCC TGGATCTAAA TTAAGATCTG ATTTAGATTT TTATTTAGAA CAAGTGAGTT CAGGAATTGG
CBE552_138   GTGTTTTTTA TCAAGATGGA CAATTTCATG ATAATAATCC TGGATCTAAA TTAAGATCTG ATTTAGATTT TTATTTAGAA CAAGTGAGTT CAGGAATTGG
CBE562_138   GTGTTTTTTA TCAAGATGGA CAATTTCATG ATAATAATCC TGGATCTAAA TTAAGATCTG ATTTAGATTT TTATTTAGAA CAAGTGAGTT CAGGAATTGG
CBE553_138   GTGTTTTTTA TCAAGATGGA CAATTTCATG ATAATAATCC TGGATCTAAA TTAAGATCTG ATTTAGATTT TTATTTAGAA CAAGTGAGTT CAGGAATTGG
CBE545_138   GTGTTTTTTA TCAAGATGGA CAATTTCATG ATAATAATCC TGGATCTAAA TTAAGATCTG ATTTAGATTT TTATTTAGAA CAAGTGAGTT CAGGAATTGG
```

```
             501                                                     551                                                600
CBE541_138   TACGAAATTT ATAACAATTT TTACATATGC CAGTTCCTTT TTAGGTTTAT TTATTTGGTC ATTAATGAAT
CBE565_138   TACGAAATTT ATAACAATTT TTACATATGC CAGTTCCTTT TTAGGTTTAT TTATTTGGTC ATTAATAAA
CBE559_138   TACGAAATTT ATAACAATTT TTACATATGC CAGTTCCTTT TTAGGTTTAT ATATTTGGTC ATTAAATAAA A
CBE558_138   TACGAAATTT ATAACAATTT TTACATATGC CAGTTCCTTT TTAGGTTTAT TTATTTGGTC ATTAATAGT
CBE555_138   TACGAAATTT ATAACAATTT TTACATATGC CAGTTCCTTT TTAGGTTTAT TTATTTGGTC ATTAATAAAA AATGCACGTT TGATACTGTT ATGTTATT
CBE550_138   TACGAAATTT ATAACAATTT TTACATATGC CAGTTCCTTT TTAGGTTTAT TTATTTGGTC ATTAATAAAA AATGCACGTT TGA--CTGTT AGGTT
CBE551_138   TACGAAATTT ATAACAATTT TTACATATGC CAGTTCCTTT TTAGGTTTAT TTATTTGGTC ATTAATAAAA AATGCACGTT TG---CT-TT ATG
CBE546_138   TACGAAATTT ATAACAATTT TTACATATGC CAGTTCCTTT TTAGGTTTAT ATATTTGGTC ATTAATAAAA AATGCACGTT TGACTTTATG TATTAACT
CBE564_138   TACGAAATTT ATAACAATTT TTACATATGC CAGTTCCTTT TTAGGTTTAT ATATTTGGTC ATTAATAAAA AATGCACGTT TGACTTTATG TATTA-CT
CBE548_138   TACGAAATTT ATAACAATTT TTACATATGC CAGTTCCTTT TTAGGTTTAT ATATTTGGTC ATTAATAAAA AATGCACGTT TGACTTTATG TATTACTT
CBE549_138   TACGAAATTT ATAACAATTT TTACATATGC CAGTTCCTTT TTAGGTTTAT ATATTTGGTC ATTAATAAAA AATGCACGTT TGACTTTATG TATTACTT
CBE653_138   TACGAAATTT ATAACAATTT TTACATATGC CAGTTCCTTT TTAGGTTTAT TTATTTGGTC ATTAATAAAA AATGCACGTT TGACTTTATG TATTACTT
CBE542_138   TACGAAATTT ATAACAATTT TTACATATGC CAGTTCCTTT TTAGGTTTAT ATATTTGGTC ATTAATAAAA AATGCACGTT TGACTTTATG TATTACTT
CBE556_138   TACGAAATTT ATAACAATTT TTACATATGC CAGTTCCTTT TTAGGTTTAT ATATTTGGTC ATTAATAAAA AATGCACGTT TGACTTTATG TATTACTT
       3D7   TACGAAATTT ATAACAATTT TTACATATGC CAGTTCCTTT TTAGGTTTAT ATATTTGGTC ATTAATAAAA AATGCACGTT TGACTTTATG TATTACTT
CBE540_138   TACGAAATTT ATAACAATTT TTACATATGC CAGTTCCTTT TTAGGTTTAT ATATTTGGTC ATTAATAAAA AATGCACGTT TGACTTTATG TATTACTT
CBE543_138   TACGAAATTT ATAACAATTT TTACATATGC CAGTTCCTTT TTAGGTTTAT ATATTTGGTC ATTAATAAAA AATGCACGTT TGACTTTATG TATTACTT
CBE554_138   TACGAAATTT ATAACAATTT TTACATATGC CAGTTCCTTT TTAGGTTTAT ATATTTGGTC ATTAATAAAA AATGCACGTT TGACTTTATG TATTACTT
CBE563_138   TACGAAATTT ATAACAATTT TTACATATGC CAGTTCCTTT TTAGGTTTAT ATATTTGGTC ATTAATAAAA AATGCACGTT TGACTTTATG TATTACTT
CBE547_138   TACGAAATTT ATAACAATTT TTACATATGC CAGTTCCTTT TTAGGTTTAT ATATTTGGTC ATTTTTAATA GA
CBE567_138   TACGAAATTT ATAACAATTG TTACATATGC CGCGTGA
CBE557_138   TACGAAATTT ATAACAGATT TTT
CBE560_138   TACGAAATTT ATAACAATTT TTACATATGC CAGTTCCTTT TTAGGTTTAT ATATTTGGTC ATTAATAAAA AATGCTCG TTTGACATTG TAT-GTAATT
CBE552_138   TACGAAATTT ATAACAATTT TTACATATGC CAGTTCCTTT TTAGGTTTAT TTATTTGGTC ATTAATAAAA AATGCACG TTTGACTTTA TGT-TTAACT
CBE562_138   TACGAAATTT ATAACAATTT TTACATATGC CAGTTCCTTT TTAGGTTTAT TTATTTGGTC ATTAATGAGA
CBE553_138   TACGAAATTT ATAACAATTT TTACATATGC CAGTTCCTTT TTAGGTTTAT ATATTTGGTC ATTTGTGAG
CBE545_138   TACGAAATTT ATAACAATTT TTACATATGC CAGTTCCTTT TTAGGTTTAT ATATTTGGTC ATTTATA
```

Samples from 2017, gene *pfmdr1* D1246Y

```
             3601                                                                                                 3700
CBE709_138   ATAATAATTC ATTGGTTTTA AAAAATGTAA ATGAATTTTC AAACCAATCT GGATCTGCAG AAGATTATAC TGTATTTAAT AATAATGGAG AAATATTATT
CBE731_138   ATAATAATTC ATTGGTTTTA AAAAATGTAA ATGAATTTTC AAACCAATCT GGATCTGCAG AAGATTATAC TGTATTTAAT AATAATGGAG AAATATTATT
```

```
CBE733_138  ATAATAATTC ATTGGTTTTA AAAAATGTAA ATGAATTTTC AAACCAATCT GGATCTGCAG AAGATTATAC TGTATTTAAT AATAATGGAG AAATATTATT
CBE715_138  ATAATAATTC ATTGGTTTTA AAAAATGTAA ATGAATTTTC AAACCAATCT GGATCTGCAG AAGATTATAC TGTATTTAAT AATAATGGAG AAATATTATT
CBE727_138  ATAATAATTC ATTGGTTTTA AAAAATGTAA ATGAATTTTC AAACCAATCT GGATCTGCAG AAGATTATAC TGTATTTAAT AATAATGGAG AAATATTATT
CBE736_138  ATAATAATTC ATTGGTTTTA AAAAATGTAA ATGAATTTTC AAACCAATCT GGATCTGCAG AAGATTATAC TGTATTTAAT AATAATGGAG AAATATTATT
CBE718_138  ATAATAATTC ATTGGTTTTA AAAAATGTAA ATGAATTTTC AAACCAATCT GGATCTGCAG AAGATTATAC TGTATTTAAT AATAATGGAG AAATATTATT
CBE724_138  ATAATAATTC ATTGGTTTTA AAAAATGTAA ATGAATTTTC AAACCAATCT GGATCTGCAG AAGATTATAC TGTATTTAAT AATAATGGAG AAATATTATT
CBE732_138  ATAATAATTC ATTGGTTTTA AAAAATGTAA ATGAATTTTC AAACCAATCT GGATCTGCAG AAGATTATAC TGTATTTAAT AATAATGGAG AAATATTATT
CBE735_138  ATAATAATTC ATTGGTTTTA AAAAATGTAA ATGAATTTTC AAACCAATCT GGATCTGCAG AAGATTATAC TGTATTTAAT AATAATGGAG AAATATTATT
       3D7  ATAATAATTC ATTGGTTTTA AAAAATGTAA ATGAATTTTC AAACCAATCT GGATCTGCAG AAGATTATAC TGTATTTAAT AATAATGGAG AAATATTATT
CBE730_138  ATAATAATTC ATTGGTTTTA AAAAATGTAA ATGAATTTTC AAACCAATCT GGATCTGCAG AAGATTATAC TGTATTTAAT AATAATGGAG AAATATTATT
CBE721_138  ATAATAATTC ATTGGTTTTA AAAAATGTAA ATGAATTTTC AAACCAATCT GGATCTGCAG AAGATTATAC TGTATTTAAT AATAATGGAG AAATATTATT
CBE726_138  ATAATAATTC ATTGGTTTTA AAAAATGTAA ATGAATTTTC AAACCAATCT GGATCTGCAG AAGATTATAC TGTATTTAAT AATAATGGAG AAATATTATT
CBE723_138  ATAATAATTC ATTGGTTTTA AAAAATGTAA ATGAATTTTC AAACCAATCT GGATCTGCAG AAGATTATAC TGTATTTAAT AATAATGGAG AAATATTATT
CBE722_138  ATAATAATTC ATTGGTTTTA AAAAATGTAA ATGAATTTTC AAACCAATCT GGATCTGCAG AAGATTATAC TGTATTTAAT AATAATGGAG AAATATTATT
CBE725_138  ATAATAATTC ATTGGTTTTA AAAAATGTAA ATGAATTTTC AAACCAATCT GGATCTGCAG AAGATTATAC TGTATTTAAT AATAATGGAG AAATATTATT
CBE729_138  ATAATAATTC ATTGGTTTTA AAAAATGTAA ATGAATTTTC AAACCAATCT GGATCTGCAG AAGATTATAC TGTATTTAAT AATAATGGAG AAATATTATT
CBE728_138  ATAATAATTC ATTGGTTTTA AAAAATGTAA ATGAATTTTC AAACCAATCT GGATCTGCAG AAGATTATAC TGTATTTAAT AATAATGGAG AAATATTATT
CBE720_138  ATAATAATTC ATTGGTTTTA AAAAATGTAA ATGAATTTTC AAACCAATCT GGATCTGCAG AAGATTATAC TGTATTTAAT AATAATGGAG AAATATTATT
CBE716_138  ATAATAATTC ATTGGTTTTA AAAAATGTAA ATGAATTTTC AAACCAATCT GGATCTGCAG AAGATTATAC TGTATTTAAT AATAATGGAG AAATATTATT
CBE712_138  ATAATAATTC ATTGGTTTTA AAAAATGTAA ATGAATTTTC AAACCAATCT GGATCTGCAG AAGATTATAC TGTATTTAAT AATAATGGAG AAATATTATT
CBE717_138  ATAATAATTC ATTGGTTTTA AAAAATGTAA ATGAATTTTC AAACCAATCT GGATCTGCAG AAGATTATAC TGTATTTAAT AATAATGGAG AAATATTATT
CBE711_138  ATAATAATTC ATTGGTTTTA AAAAATGTAA ATGAATTTTC AAACCAATCT GGATCTGCAG AAGATTATAC TGTATTTAAT AATAATGGAG AAATATTATT
CBE734_138                              AAATGTAA ATGAATTTTC AAACCAATCT GGATCTGCAG AAGATTATAC TGTATTTAAT AATAATGGAG AAATATTATT
CBE710_138                                                                                 TGTATTTAAT AATAATGGAG AAATATTATT

            3701                              3738                                                                   3800
CBE709_138  AGATGATATT AATATATGTG ATTATAACTT AAGAGATCTT AGAAACTTAT TTTCAATAGT TAGTCAAGAA CCCATGTTAT TTAATATGTC CATATATGAA
CBE731_138  AGATGATATT AATATATGTG ATTATAACTT AAGAGATCTT AGAAACTTAT TTTCAATAGT TAGTCAAGAA CCCATGTTAT TTAATATGTC CATATATGAA
CBE733_138  AGATGATATT AATATATGTG ATTATAACTT AAGAGATCTT AGAAACTTAT TTTCAATAGT TAGTCAAGAA CCCATGTTAT TTAATATGTC CATATATGAA
CBE715_138  AGATGATATT AATATATGTG ATTATAACTT AAGAGATCTT AGAAACTTAT TTTCAATAGT TAGTCAAGAA CCCATGTTAT TTAATATGTC CATATATGAA
CBE727_138  AGATGATATT AATATATGTG ATTATAACTT AAGAGATCTT AGAAACTTAT TTTCAATAGT TAGTCAAGAA CCCATGTTAT TTAATATGTC CATATATGAA
CBE736_138  AGATGATATT AATATATGTG ATTATAACTT AAGAGATCTT AGAAACTTAT TTTCAATAGT TAGTCAAGAA CCCATGTTAT TTAATATGTC CATATATGAA
CBE718_138  AGATGATATT AATATATGTG ATTATAACTT AAGAGATCTT AGAAACTTAT TTTCAATAGT TAGTCAAGAA CCCATGTTAT TTAATATGTC CATATATGAA
CBE724_138  AGATGATATT AATATATGTG ATTATAACTT AAGAGATCTT AGAAACTTAT TTTCAATAGT TAGTCAAGAA CCCATGTTAT TTAATATGTC CATATATGAA
CBE732_138  AGATGATATT AATATATGTG ATTATAACTT AAGAGATCTT AGAAACTTAT TTTCAATAGT TAGTCAAGAA CCCATGTTAT TTAATATGTC CATATATGAA
CBE735_138  AGATGATATT AATATATGTG ATTATAACTT AAGAGATCTT AGAAACTTAT TTTCAATAGT TAGTCAAGAA CCCATGTTAT TTAATATGTC CATATATGAA
       3D7  AGATGATATT AATATATGTG ATTATAACTT AAGAGATCTT AGAAACTTAT TTTCAATAGT TAGTCAAGAA CCCATGTTAT TTAATATGTC CATATATGAA
CBE730_138  AGATGATATT AATATATGTG ATTATAACTT AAGAGATCTT AGAAACTTAT TTTCAATAGT TAGTCAAGAA CCCATGTTAT TTAATATGTC CATATATGAA
CBE721_138  AGATGATATT AATATATGTG ATTATAACTT AAGAGATCTT AGAAACTTAT TTTCAATAGT TAGTCAAGAA CCCATGTTAT TTAATATGTC CATATATGAA
CBE726_138  AGATGATATT AATATATGTG ATTATAACTT AAGAGATCTT AGAAACTTAT TTTCAATAGT TAGTCAAGAA CCCATGTTAT TTAATATGTC CATATATGAA
CBE723_138  AGATGATATT AATATATGTG ATTATAACTT AAGAGATCTT AGAAACTTAT TTTCAATAGT TAGTCAAGAA CCCATGTTAT TTAATATGTC CATATATGAA
CBE722_138  AGATGATATT AATATATGTG ATTATAACTT AAGAGATCTT AGAAACTTAT TTTCAATAGT TAGTCAAGAA CCCATGTTAT TTAATATGTC CATATATGAA
CBE725_138  AGATGATATT AATATATGTG ATTATAACTT AAGAGATCTT AGAAACTTAT TTTCAATAGT TAGTCAAGAA CCCATGTTAT TTAATATGTC CATATATGAA
CBE729_138  AGATGATATT AATATATGTG ATTATAACTT AAGAGATCTT AGAAACTTAT TTTCAATAGT TAGTCAAGAA CCCATGTTAT TTAATATGTC CATATATGAA
CBE728_138  AGATGATATT AATATATGTG ATTATAACTT AAGAGATCTT AGAAACTTAT TTTCAATAGT TAGTCAAGAA CCCATGTTAT TTAATATGTC CATATATGAA
CBE720_138  AGATGATATT AATATATGTG ATTATAACTT AAGAGATCTT AGAAACTTAT TTTCAATAGT TAGTCAAGAA CCCATGTTAT TTAATATGTC CATATATGAA
```

```
CBE716_138  AGATGATATT AATATATGTG ATTATAACTT AAGAGATCTT AGAAACTTAT TTTCAATAGT TAGTCAAGAA CCCATGTTAT TTAATATGTC CATATATGAA
CBE712_138  AGATGATATT AATATATGTG ATTATAACTT AAGATATCTT AGAAACTTAT TTTCAATAGT TAGTCAAGAA CCCATGTTAT TTAATATGTC CATATATGAA
CBE717_138  AGATGATATT AATATATGTG ATTATAACTT AAGAGATCTT AGAAACTTAT TTTCAATAGT TAGTCAAGAA CCCATGTTAT TTAATATGTC CATATATGAA
CBE711_138  AGATGATATT AATATATGTG ATTATAACTT AAGATATCTT AGAAACTTAT TTTCAATAGT TAGTCAAGAA CCCATGTTAT TTAATATGTC CATATATGAA
CBE734_138  AGATGATATT AATATATGTG ATTATAACTT AAGAGATCTT AGAAACTTAT TTTCAATAGT TAGTCAAGAA CCCATGTTAT TTAATATGTC CATATATGAA
CBE710_138  AGATGATATT AATATATGTG ATTATAACTT AAGAGATCTT AGAAACTTAT TTTCAATAGT TAGTCAAGAA CCCATGTTAT TTAATATGTC CATATATGAA

            3801                                                                                                   3900
CBE709_138  AATATCAAAT TTGGAAGAGA AGATGCAACA TTGGAAGATG TTAAACGTGT TAGTAAGTTT GCTGCTATAG ATGAATTTAT CGAATCATTA CCAAATAAAT
CBE731_138  AATATCAAAT TTGGAAGAGA AGATGCAACA TTGGAAGATG TTAAACGTGT TAGTAAGTTT GCTGCTATAG ATGAATTTAT CGAATCATTA CCAAATAAAT
CBE733_138  AATATCAAAT TTGGAAGAGA AGATGCAACA TTGGAAGATG TTAAACGTGT TAGTAAGTTT GCTGCTATAG ATGAATTTAT CGAATCATTA CCAAATAAAT
CBE715_138  AATATCAAAT TTGGAAGAGA AGATGCAACA TTGGAAGATG TTAAACGTGT TAGTAAGTTT GCTGCTATAG ATGAATTTAT CGAATCATTA CCAAATAAAT
CBE727_138  AATATCAAAT TTGGAAGAGA AGATGCAACA TTGGAAGATG TTAAACGTGT TAGTAAGTTT GCTGCTATAG ATGAATTTAT CGAATCATTA CCAAATAAAT
CBE736_138  AATATCAAAT TTGGAAGAGA AGATGCAACA TTGGAAGATG TTAAACGTGT TAGTAAGTTT GCTGCTATAG ATGAATTTAT CGAATCATTA CCAAATAAAT
CBE718_138  AATATCAAAT TTGGAAGAGA AGATGCAACA TTGGAAGATG TTAAACGTGT TAGTAAGTTT GCTGCTATAG ATGAATTTAT CGAATCATTA CCAAATAAAT
CBE724_138  AATATCAAAT TTGGAAGAGA AGATGCAACA TTGGAAGATG TTAAACGTGT TAGTAAGTTT GCTGCTATAG ATGAATTTAT CGAATCATTA CCAAATAAAT
CBE732_138  AATATCAAAT TTGGAAGAGA AGATGCAACA TTGGAAGATG TTAAACGTGT TAGTAAGTTT GCTGCTATAG ATGAATTTAT CGAATCATTA CCAAATAAAT
CBE735_138  AATATCAAAT TTGGAAGAGA AGATGCAACA TTGGAAGATG TTAAACGTGT TAGTAAGTTT GCTGCTATAG ATGAATTTAT CGAATCATTA CCAAATAAAT
       3D7  AATATCAAAT TTGGAAGAGA AGATGCAACA TTGGAAGATG TTAAACGTGT TAGTAAGTTT GCTGCTATAG ATGAATTTAT CGAATCATTA CCAAATAAAT
CBE730_138  AATATCAAAT TTGGAAGAGA AGATGCAACA TTGGAAGATG TTAAACGTGT TAGTAAGTTT GCTGCTATAG ATGAATTTAT CGAATCATTA CCAAATAAAT
CBE721_138  AATATCAAAT TTGGAAGAGA AGATGCAACA TTGGAAGATG TTAAACGTGT TAGTAAGTTT GCTGCTATAG ATGAATTTAT CGAATCATTA CCAAATAAAT
CBE726_138  AATATCAAAT TTGGAAGAGA AGATGCAACA TTGGAAGATG TTAAACGTGT TAGTAAGTTT GCTGCTATAG ATGAATTTAT CGAATCATTA CCAAATAAAT
CBE723_138  AATATCAAAT TTGGAAGAGA AGATGCAACA TTGGAAGATG TTAAACGTGT TAGTAAGTTT GCTGCTATAG ATGAATTTAT CGAATCATTA CCAAATAAAT
CBE722_138  AATATCAAAT TTGGAAGAGA AGATGCAACA TTGGAAGATG TTAAACGTGT TAGTAAGTTT GCTGCTATAG ATGAATTTAT CGAATCATTA CCAAATAAAT
CBE725_138  AATATCAAAT TTGGAAGAGA AGATGCAACA TTGGAAGATG TTAAACGTGT TAGTAAGTTT GCTGCTATAG ATGAATTTAT CGAATCATTA CCAAATAAAT
CBE729_138  AATATCAAAT TTGGAAGAGA AGATGCAACA TTGGAAGATG TTAAACGTGT TAGTAAGTTT GCTGCTATAG ATGAATTTAT CGAATCATTA CCAAATAAAT
CBE728_138  AATATCAAAT TTGGAAGAGA AGATGCAACA TTGGAAGATG TTAAACGTGT TAGTAAGTTT GCTGCTATAG ATGAATTTAT CGAATCATTA CCAAATAAAT
CBE720_138  AATATCAAAT TTGGAAGAGA AGATGCAACA TTGGAAGATG TTAAACGTGT TAGTAAGTTT GCTGCTATAG ATGAATTTAT CGAATCATTA CCAAATAAAT
CBE716_138  AATATCAAAT TTGGAAGAGA AGATGCAACA TTGGAAGATG TTAAACGTGT TAGTAAGTTT GCTGCTATAG ATGAATTTAT CGAATCATTA CCAAATAAAT
CBE712_138  AATATCAAAT TTGGAAGAGA AGATGCAACA TTGGAAGATG TTAAACGTGT TAGTAAGTTT GCTGCTATAG ATGAATTTAT CGAATCATTA CCAAATAAAT
CBE717_138  AATATCAAAT TTGGAAGAGA AGATGCAACA TTGGAAGATG TTAAACGTGT TAGTAAGTTT GCTGCTATAG ATGAATTTAT CGAATCATTA CCAAATAAAT
CBE711_138  AATATCAAAT TTGGAAGAGA AGATGCAACA TTGGAAGATG TTAAACGTGT TAGTAAGTTT GCTGCTATAG ATGAATTTAT CGAATCATTA CCAAATAAAT
CBE734_138  AATATCAAAT TTGGAAGAGA AGATGCAACA TTGGAAGATG TTAAACGTGT TAGTAAGTTT GCTGCTATAG ATGAATTTAT CGAATCATTA CCAAATAAAT
CBE710_138  AATATCAAAT TTGGAAGAGA AGATGCAACA TTGGAAGATG TTAAACGTGT TAGTAAGTTT GCTGCTATAG ATGAATTTAT CGAATCATTA CCAAATAAAT
```
