# Supplementary material for: Prevalence of pfk13 and pfmdr1 polymorphisms in Bounkiling, Southern Senegal
Source: PLoS One. 2021 Mar 26;16(3):e0249357. doi: 10.1371/journal.pone.0249357 (PMC7996989; doi:10.1371/journal.pone.0249357)
Supplement: S3 File — (PDF) [file pone.0249357.s003.pdf]

Senegal Sequences *pfK13*
2015

```
             1401                                                                                                  1500
         3D7 TCAACAATGC TGGCGTATGT GTACACCTAT GTCTACCAAA AAAGCTTATT TTGGAAGTGC TGTATTGAAT AATTTCTTAT ACGTTTTTGG TGGTAATAAC
     CBE466_ TCAACAATGC TGGCGTATGT GTACACCTAT GTCTACCAAA AAAGCTTATT TTGGAAGTGC TGTATTGAAT AATTTCTTAT ACGTTTTTGG TGGTAATAAC
  BWF511_125 TCAACAATGC TGGCGTATGT GTACACCTAT GTCTACCAAA AAAGCTTATT TTGGAAGTGC TGTATTGAAT AATTTCTTAT ACGTTTTTGG TGGTAATAAC
  BWF513_125 TCAACAATGC TGGCGTATGT GTACACCTAT GTCTACCAAA AAAGCTTATT TTGGAAGTGC TGTATTGAAT AATTTCTTAT ACGTTTTTGG TGGTAATAAC
  BWF499_125 TCAACAATGC TGGCGTATGT GTACACCTAT GTCTACCAAA AAAGCTTATT TTGGAAGTGC TGTATTGAAT AATTTCTTAT ACGTTTTTGG TGGTAATAAC
  BWF502_125 TCAACAATGC TGGCGTATGT GTACACCTAT GTCTACCAAA AAAGCTTATT TTGGAAGTGC TGTATTGAAT AATTTCTTAT ACGTTTTTGG TGGTAATAAC
  BWF524_125 TCAACAATGC TGGCGTATGT GTACACCTAT GTCTACCAAA AAAGCTTATT TTGGAAGTGC TGTATTGAAT AATTTCTTAT ACGTTTTTGG TGGTAATAAC
  BWF518_125 TCAACAATGC TGGCGTATGT GTACACCTAT GTCTACCAAA AAAGCTTATT TTGGAAGTGC TGTATTGAAT AATTTCTTAT ACGTTTTTGG TGGTAATAAC
  BWF520_125 TCAACAATGC TGGCGTATGT GTACACCTAT GTCTACCAAA AAAGCTTATT TTGGAAGTGC TGTATTGAAT AATTTCTTAT ACGTTTTTGG TGGTAATAAC
  BWF506_125 TCAACAATGC TGGCGTATGT GTACACCTAT GTCTACCAAA AAAGCTTATT TTGGAAGTGC TGTATTGAAT AATTTCTTAT ACGTTTTTGG TGGTAATAAC
  BWF504_125 TCAACAATGC TGGCGTATGT GTACACCTAT GTCTACCAAA AAAGCTTATT TTGGAAGTGC TGTATTGAAT AATTTCTTAT ACGTTTTTGG TGGTAATAAC
  BWF516_125 TCAACAATGC TGGCGTATGT GTACACCTAT GTCTACCAAA AAAGCTTATT TTGGAAGTGC TGTATTGAAT AATTTCTTAT ACGTTTTTGG TGGTAATAAC
  BWF503_125 TCAACAATGC TGGCGTATGT GTACACCTAT GTCTACCAAA AAAGCTTATT TTGGAAGTGC TGTATTGAAT AATTTCTTAT ACGTTTTTGG TGGTAATAAC
  BWF495_125 TCAACAATGC TGGCGTATGT GTACACCTAT GTCTACCAAA AAAGCTTATT TTGGAAGTGC TGTATTGAAT AATTTCTTAT ACGTTTTTGG TGGTAATAAC
  BWF515_125 TCAACAATGC TGGCGTATGT GTACACCTAT GTCTACCAAA AAAGCTTATT TTGGAAGTGC TGTATTGAAT AATTTCTTAT ACGTTTTTGG TGGTAATAAC
  BWF505_125 TCAACAATGC TGGCGTATGT GTACACCTAT GTCTACCAAA AAAGCTTATT TTGGAAGTGC TGTATTGAAT AATTTCTTAT ACGTTTTTGG TGGTAATAAC
  BWF509_125 TCAACAATGC TGGCGTATGT GTACACCTAT GTCTACCAAA AAAGCTTATT TTGGAAGTGC TGTATTGAAT AATTTCTTAT ACGTTTTTGG TGGTAATAAC
  BWF507_125 TCAACAATGC TGGCGTATGT GTACACCTAT GTCTACCAAA AAAGCTTATT TTGGAAGTGC TGTATTGAAT AATTTCTTAT ACGTTTTTGG TGGTAATAAC
  BWF497_125 TCAACAATGC TGGCGTATGT GTACACCTAT GTCTACCAAA AAAGCTTATT TTGGAAGTGC TGTATTGAAT AATTTCTTAT ACGTTTTTGG TGGTAATAAC
  BWF512_125 TCAACAATGC TGGCGTATGT GTACACCTAT GTCTACCAAA AAAGCTTATT TTGGAAGTGC TGTATTGAAT AATTTCTTAT ACGTTTTTGG TGGTAATAAC
  BWF510_125 TCAACAATGC TGGCGTATGT GTACACCTAT GTCTACCAAA AAAGCTTATT TTGGAAGTGC TGTATTGAAT AATTTCTTAT ACGTTTTTGG TGGTAATAAC
  BWF519_125 TCAACAATGC TGGCGTATGT GTACACCTAT GTCTACCAAA AAAGCTTATT TTGGAAGTGC TGTATTGAAT AATTTCTTAT ACGTTTTTGG TGGTAATAAC
  BWF498_125 TCAACAATGC TGGCGTATGT GTACACCTAT GTCTACCAAA AAAGCTTATT TTGGAAGTGC TGTATTGAAT AATTTCTTAT ACGTTTTTGG TGGTAATAAC
  BWF508_125 TCAACAATGC TGGCGTATGT GTACACCTAT GTCTACCAAA AAAGCTTATT TTGGAAGTGC TGTATTGAAT AATTTCTTAT ACGTTTTTGG TGGTAATAAC
  BWF493_125 TCAACAATGC TGGCGTATGT GTACACCTAT GTCTACCAAA AAAGCTTATT TTGGAAGTGC TGTATTGAAT AATTTCTTAT ACGTTTTTGG TGGTAATAAC
  BWF496_125 TCAACAATGC TGGCGTATGT GTACACCTAT GTCTACCAAA AAAGCTTATT TTGGAAGTGC TGTATTGAAT AATTTCTTAT ACGTTTTTGG TGGTAATAAC
  BWF514_125 TCAACAATGC TGGCGTATGT GTACACCTAT GTCTACCAAA AAAGCTTATT TTGGAAGTGC TGTATTGAAT AATTTCTTAT ACGTTTTTGG TGGTAATAAC
      CBE463 TCAACAATGC TGGCGTATGT GTACACCTAT GTCTACCAAA AAAGCTTATT TTGGAAGTGC TGTATTGAAT AATTTCTTAT ACGTTTTTGG TGGTAATAAC
      CBE464 TCAACAATGC TGGCGTATGT GTACACCTAT GTCTACCAAA AAAGCTTATT TTGGAAGTGC TGTATTGAAT AATTTCTTAT ACGTTTTTGG TGGTAATAAC
      CBE467 TCAACAATGC TGGCGTATGT GTACACCTAT GTCTACCAAA AAAGCTTATT TTGGAAGTGC TGTATTGAAT AATTTCTTAT ACGTTTTTGG TGGTAATAAC
      CBE468 TCAACAATGC TGGCGTATGT GTACACCTAT GTCTACCAAA AAAGCTTATT TTGGAAGTGC TGTATTGAAT AATTTCTTAT ACGTTTTTGG TGGTAATAAC
  BWF500_125 TCAACAATGC TGGCGTATGT GTACACCTAT GTCTACCAAA AAAGCTTATT TTGGAAGTGC TGTATTGAAT AATTTCTTAT ACGTTTTTGG TGGTAATAAC
  BWF522_125 TCAACAATGC TGGCGTATGT GTACACCTAT GTCTACCAAA AAAGCTTATT TTGGAAGTGC TGTATTGAAT AATTTCTTAT ACGTTTTTGG TGGTAATAAC
   CBE465_17 TCAACAATGC TGGCGTATGT GTACACCTAT GTCTACCAAA AAAGCTTATT TTGGAAGTGC TGTATTGAAT AATTTCTTAT ACGTTTTTGG TGGTAATAAC

             1501                                                                                                  1600
         3D7 TATGATTATA AGGCTTTATT TGAAACTGAG GTGTATGATC GTTTAAGAGA TGTATGGTAT GTTTCAAGTA ATTTAAATAT ACCTAGAAGA AATAATTGTG
```

```
   CBE466_  TATGATTATA AGGCTTTATT TGAAACTGAG GTGTATGATC GTTTAAGAGA TGTATGGTAT GTTTCAAGTA ATTTAAATAT ACCTAGAAGA AATAATTGTG
BWF511_125  TATGATTATA AGGCTTTATT TGAAACTGAG GTGTATGATC GTTTAAGAGA TGTATGGTAT GTTTCAAGTA ATTTAAATAT ACCTAGAAGA AATAATTGTG
BWF513_125  TATGATTATA AGGCTTTATT TGAAACTGAG GTGTATGATC GTTTAAGAGA TGTATGGTAT GTTTCAAGTA ATTTAAATAT ACCTAGAAGA AATAATTGTG
BWF499_125  TATGATTATA AGGCTTTATT TGAAACTGAG GTGTATGATC GTTTAAGAGA TGTATGGTAT GTTTCAAGTA ATTTAAATAT ACCTAGAAGA AATAATTGTG
BWF502_125  TATGATTATA AGGCTTTATT TGAAACTGAG GTGTATGATC GTTTAAGAGA TGTATGGTAT GTTTCAAGTA ATTTAAATAT ACCTAGAAGA AATAATTGTG
BWF524_125  TATGATTATA AGGCTTTATT TGAAACTGAG GTGTATGATC GTTTAAGAGA TGTATGGTAT GTTTCAAGTA ATTTAAATAT ACCTAGAAGA AATAATTGTG
BWF518_125  TATGATTATA AGGCTTTATT TGAAACTGAG GTGTATGATC GTTTAAGAGA TGTATGGTAT GTTTCAAGTA ATTTAAATAT ACCTAGAAGA AATAATTGTG
BWF520_125  TATGATTATA AGGCTTTATT TGAAACTGAG GTGTATGATC GTTTAAGAGA TGTATGGTAT GTTTCAAGTA ATTTAAATAT ACCTAGAAGA AATAATTGTG
BWF506_125  TATGATTATA AGGCTTTATT TGAAACTGAG GTGTATGATC GTTTAAGAGA TGTATGGTAT GTTTCAAGTA ATTTAAATAT ACCTAGAAGA AATAATTGTG
BWF504_125  TATGATTATA AGGCTTTATT TGAAACTGAG GTGTATGATC GTTTAAGAGA TGTATGGTAT GTTTCAAGTA ATTTAAATAT ACCTAGAAGA AATAATTGTG
BWF516_125  TATGATTATA AGGCTTTATT TGAAACTGAG GTGTATGATC GTTTAAGAGA TGTATGGTAT GTTTCAAGTA ATTTAAATAT ACCTAGAAGA AATAATTGTG
BWF503_125  TATGATTATA AGGCTTTATT TGAAACTGAG GTGTATGATC GTTTAAGAGA TGTATGGTAT GTTTCAAGTA ATTTAAATAT ACCTAGAAGA AATAATTGTG
BWF495_125  TATGATTATA AGGCTTTATT TGAAACTGAG GTGTATGATC GTTTAAGAGA TGTATGGTAT GTTTCAAGTA ATTTAAATAT ACCTAGAAGA AATAATTGTG
BWF515_125  TATGATTATA AGGCTTTATT TGAAACTGAG GTGTATGATC GTTTAAGAGA TGTATGGTAT GTTTCAAGTA ATTTAAATAT ACCTAGAAGA AATAATTGTG
BWF505_125  TATGATTATA AGGCTTTATT TGAAACTGAG GTGTATGATC GTTTAAGAGA TGTATGGTAT GTTTCAAGTA ATTTAAATAT ACCTAGAAGA AATAATTGTG
BWF509_125  TATGATTATA AGGCTTTATT TGAAACTGAG GTGTATGATC GTTTAAGAGA TGTATGGTAT GTTTCAAGTA ATTTAAATAT ACCTAGAAGA AATAATTGTG
BWF507_125  TATGATTATA AGGCTTTATT TGAAACTGAG GTGTATGATC GTTTAAGAGA TGTATGGTAT GTTTCAAGTA ATTTAAATAT ACCTAGAAGA AATAATTGTG
BWF497_125  TATGATTATA AGGCTTTATT TGAAACTGAG GTGTATGATC GTTTAAGAGA TGTATGGTAT GTTTCAAGTA ATTTAAATAT ACCTAGAAGA AATAATTGTG
BWF512_125  TATGATTATA AGGCTTTATT TGAAACTGAG GTGTATGATC GTTTAAGAGA TGTATGGTAT GTTTCAAGTA ATTTAAATAT ACCTAGAAGA AATAATTGTG
BWF510_125  TATGATTATA AGGCTTTATT TGAAACTGAG GTGTATGATC GTTTAAGAGA TGTATGGTAT GTTTCAAGTA ATTTAAATAT ACCTAGAAGA AATAATTGTG
BWF519_125  TATGATTATA AGGCTTTATT TGAAACTGAG GTGTATGATC GTTTAAGAGA TGTATGGTAT GTTTCAAGTA ATTTAAATAT ACCTAGAAGA AATAATTGTG
BWF498_125  TATGATTATA AGGCTTTATT TGAAACTGAG GTGTATGATC GTTTAAGAGA TGTATGGTAT GTTTCAAGTA ATTTAAATAT ACCTAGAAGA AATAATTGTG
BWF508_125  TATGATTATA AGGCTTTATT TGAAACTGAG GTGTATGATC GTTTAAGAGA TGTATGGTAT GTTTCAAGTA ATTTAAATAT ACCTAGAAGA AATAATTGTG
BWF493_125  TATGATTATA AGGCTTTATT TGAAACTGAG GTGTATGATC GTTTAAGAGA TGTATGGTAT GTTTCAAGTA ATTTAAATAT ACCTAGAAGA AATAATTGTG
BWF496_125  TATGATTATA AGGCTTTATT TGAAACTGAG GTGTATGATC GTTTAAGAGA TGTATGGTAT GTTTCAAGTA ATTTAAATAT ACCTAGAAGA AATAATTGTG
BWF514_125  TATGATTATA AGGCTTTATT TGAAACTGAG GTGTATGATC GTTTAAGAGA TGTATGGTAT GTTTCAAGTA ATTTAAATAT ACCTAGAAGA AATAATTGTG
    CBE463  TATGATTATA AGGCTTTATT TGAAACTGAG GTGTATGATC GTTTAAGAGA TGTATGGTAT GTTTCAAGTA ATTTAAATAT ACCTAGAAGA AATAATTGTG
    CBE464  TATGATTATA AGGCTTTATT TGAAACTGAG GTGTATGATC GTTTAAGAGA TGTATGGTAT GTTTCAAGTA ATTTAAATAT ACCTAGAAGA AATAATTGTG
    CBE467  TATGATTATA AGGCTTTATT TGAAACTGAG GTGTATGATC GTTTAAGAGA TGTATGGTAT GTTTCAAGTA ATTTAAATAT ACCTAGAAGA AATAATTGTG
    CBE468  TATGATTATA AGGCTTTATT TGAAACTGAG GTGTATGATC GTTTAAGAGA TGTATGGTAT GTTTCAAGTA ATTTAAATAT ACCTAGAAGA AATAATTGTG
BWF500_125  TATGATTATA AGGCTTTATT TGAAACTGAG GTGTATGATC GTTTAAGAGA TGTATGGTAT GTTTCAAGTA ATTTAAATAT ACCTAGAAGA AATAATTGTG
BWF522_125  TATGATTATA AGGCTTTATT TGAAACTGAG GTGTATGATC GTTTAAGAGA TGTATGGTAT GTTTCAAGTA ATTTAAATAT ACCTAGAAGA AATAATTGTG
 CBE465_17  TATGATTATA AGGCTTTATT TGAAACTGAG GTGTATGATC GTTTAAGAGA TGTATGGTAT GTTTCAAGTA ATTTAAATAT ACCTAGAAGA AATAATTGTG

            1601                                                                                                         1700
       3D7  GTGTTACGTC AAATGGTAGA ATTTATTGTA TTGGGGGATA TGATGGCTCT TCTATTATAC CCGAATGTAG AAGCATATGA TCATCGTATG AAAGCATGGG
   CBE466_  GTGTTACGTC AAATGGTAGA ATTTATTGTA TTGGGGGATA TGATGGCTCT TCTATTATAC CCGAATGTAG AAGCATATGA TCATCGTATG AAAGCATGGG
BWF511_125  GTGTTACGTC AAATGGTAGA ATTTATTGTA TTGGGGGATA TGATGGCTCT TCTATTATAC CCGAATGTAG AAGCATATGA TCATCGTATG AAAGCATGGG
BWF513_125  GTGTTACGTC AAATGGTAGA ATTTATTGTA TTGGGGGATA TGATGGCTCT TCTATTATAC CCGAATGTAG AAGCATATGA TCATCGTATG AAAGCATGGG
BWF499_125  GTGTTACGTC AAATGGTAGA ATTTATTGTA TTGGGGGATA TGATGGCTCT TCTATTATAC CCGAATGTAG AAGCATATGA TCATCGTATG AAAGCATGGG
BWF502_125  GTGTTACGTC AAATGGTAGA ATTTATTGTA TTGGGGGATA TGATGGCTCT TCTATTATAC CCGAATGTAG AAGCATATGA TCATCGTATG AAAGCATGGG
```

```
BWF524_125  GTGTTACGTC AAATGGTAGA ATTTATTGTA TTGGGGGATA TGATGGCTCT TCTATTATAC CCGAATGTAG AAGCATATGA TCATCGTATG AAAGCATGGG
BWF518_125  GTGTTACGTC AAATGGTAGA ATTTATTGTA TTGGGGGATA TGATGGCTCT TCTATTATAC CCGAATGTAG AAGCATATGA TCATCGTATG AAAGCATGGG
BWF520_125  GTGTTACGTC AAATGGTAGA ATTTATTGTA TTGGGGGATA TGATGGCTCT TCTATTATAC CCGAATGTAG AAGCATATGA TCATCGTATG AAAGCATGGG
BWF506_125  GTGTTACGTC AAATGGTAGA ATTTATTGTA TTGGGGGATA TGATGGCTCT TCTATTATAC CCGAATGTAG AAGCATATGA TCATCGTATG AAAGCATGGG
BWF504_125  GTGTTACGTC AAATGGTAGA ATTTATTGTA TTGGGGGATA TGATGGCTCT TCTATTATAC CCGAATGTAG AAGCATATGA TCATCGTATG AAAGCATGGG
BWF516_125  GTGTTACGTC AAATGGTAGA ATTTATTGTA TTGGGGGATA TGATGGCTCT TCTATTATAC CCGAATGTAG AAGCATATGA TCATCGTATG AAAGCATGGG
BWF503_125  GTGTTACGTC AAATGGTAGA ATTTATTGTA TTGGGGGATA TGATGGCTCT TCTATTATAC CCGAATGTAG AAGCATATGA TCATCGTATG AAAGCATGGG
BWF495_125  GTGTTACGTC AAATGGTAGA ATTTATTGTA TTGGGGGATA TGATGGCTCT TCTATTATAC CCGAATGTAG AAGCATATGA TCATCGTATG AAAGCATGGG
BWF515_125  GTGTTACGTC AAATGGTAGA ATTTATTGTA TTGGGGGATA TGATGGCTCT TCTATTATAC CCGAATGTAG AAGCATATGA TCATCGTATG AAAGCATGGG
BWF505_125  GTGTTACGTC AAATGGTAGA ATTTATTGTA TTGGGGGATA TGATGGCTCT TCTATTATAC CCGAATGTAG AAGCATATGA TCATCGTATG AAAGCATGGG
BWF509_125  GTGTTACGTC AAATGGTAGA ATTTATTGTA TTGGGGGATA TGATGGCTCT TCTATTATAC CCGAATGTAG AAGCATATGA TCATCGTATG AAAGCATGGG
BWF507_125  GTGTTACGTC AAATGGTAGA ATTTATTGTA TTGGGGGATA TGATGGCTCT TCTATTATAC CCGAATGTAG AAGCATATGA TCATCGTATG AAAGCATGGG
BWF497_125  GTGTTACGTC AAATGGTAGA ATTTATTGTA TTGGGGGATA TGATGGCTCT TCTATTATAC CCGAATGTAG AAGCATATGA TCATCGTATG AAAGCATGGG
BWF512_125  GTGTTACGTC AAATGGTAGA ATTTATTGTA TTGGGGGATA TGATGGCTCT TCTATTATAC CCGAATGTAG AAGCATATGA TCATCGTATG AAAGCATGGG
BWF510_125  GTGTTACGTC AAATGGTAGA ATTTATTGTA TTGGGGGATA TGATGGCTCT TCTATTATAC CCGAATGTAG AAGCATATGA TCATCGTATG AAAGCATGGG
BWF519_125  GTGTTACGTC AAATGGTAGA ATTTATTGTA TTGGGGGATA TGATGGCTCT TCTATTATAC CCGAATGTAG AAGCATATGA TCATCGTATG AAAGCATGGG
BWF498_125  GTGTTACGTC AAATGGTAGA ATTTATTGTA TTGGGGGATA TGATGGCTCT TCTATTATAC CCGAATGTAG AAGCATATGA TCATCGTATG AAAGCATGGG
BWF508_125  GTGTTACGTC AAATGGTAGA ATTTATTGTA TTGGGGGATA TGATGGCTCT TCTATTATAC CCGAATGTAG AAGCATATGA TCATCGTATG AAAGCATGGG
BWF493_125  GTGTTACGTC AAATGGTAGA ATTTATTGTA TTGGGGGATA TGATGGCTCT TCTATTATAC CCGAATGTAG AAGCATATGA TCATCGTATG AAAGCATGGG
BWF496_125  GTGTTACGTC AAATGGTAGA ATTTATTGTA TTGGGGGATA TGATGGCTCT TTTATTATAC CCGAATGTAG AAGCATATGA TCATCGTATG AAAGCATGGG
BWF514_125  GTGTTACGTC AAATGGTAGA ATTTATTGTA TTGGGGGATA TGATGGCTCT TCTATTATAC CCGAATGTAG AAGCATATGA TCATCGTATG AAAGCATGGG
    CBE463  GTGTTACGTC AAATGGTAGA ATTTATTGTA TTGGGGGATA TGATGGCTCT TCTATTATAC CCGAATGTAG AAGCATATGA TCATCGTATG AAAGCATGGG
    CBE464  GTGTTACGTC AAATGGTAGA ATTTATTGTA TTGGGGGATA TGATGGCTCT TCTATTATAC CCGAATGTAG AAGCATATGA TCATCGTATG AAAGCATGGG
    CBE467  GTGTTACGTC AAATGGTAGA ATTTATTGTA TTGGGGGATA TGATGGCTCT TCTATTATAC CCGAATGTAG AAGCATATGA TCATCGTATG AAAGCATGGG
    CBE468  GTGTTACGTC AAATGGTAGA ATTTATTGTA TTGGGGGATA TGATGGCTCT TCTATTATAC CCGAATGTAG AAGCATATGA TCATCGTATG AAAGCATGGG
BWF500_125  GTGTTACGTC AAATGGTAGA ATTTATTGTA TTGGGGGATA TGATGGCTCT TCTATTATAC CCGAATGTAG AAGCATATGA TCATCGTATG AAAGCATGGG
BWF522_125  GTGTTACGTC AAATGGTAGA ATTTATTGTA TTGGGGGATA TGATGGCTCT TCTATTATAC CCGAATGTAG AAGCATATGA TCATCGTATG AAAGCATGGG
 CBE465_17  GTGTTACGTC AAATGGTAGA ATTTATTGTA TTGGGGGATA TGATGGCTCT TCTATTATAC CCGAATGTAG AAGCATATGA TCATCGTATG AAAGCATGGG

            1701                                                                                               1800
       3D7  TAGAGGTGGC ACCTTTGAAT ACCCCTAGAT CATCAGCTAT GTGTGTTGCT TTTGATAATA AAATTTATGT CATTGGTGGA ACTAATGGTG AGAGATTAAA
   CBE466_  TAGAGGTGGC ACCTTTGAAT ACCCCTAGAT CATCAGCTAT GTGTGTTGCT TTTGATAATA AAATTTATGT CATTGGTGGA ACTAATGGTG AGAGATTAAA
BWF511_125  TAGAGGTGGC ACCTTTGAAT ACCCCTAGAT CATCAGCTAT GTGTGTTGCT TTTGATAATA AAATTTATGT CATTGGTGGA ACTAATGGTG AGAGATTAAA
BWF513_125  TAGAGGTGGC ACCTTTGAAT ACCCCTAGAT CATCAGCTAT GTGTGTTGCT TTTGATAATA AAATTTATGT CATTGGTGGA ACTAATGGTG AGAGATTAAA
BWF499_125  TAGAGGTGGC ACCTTTGAAT ACCCCTAGAT CATCAGCTAT GTGTGTTGCT TTTGATAATA AAATTTATGT CATTGGTGGA ACTAATGGTG AGAGATTAAA
BWF502_125  TAGAGGTGGC ACCTTTGAAT ACCCCTAGAT CATCAGCTAT GTGTGTTGCT TTTGATAATA AAATTTATGT CATTGGTGGA ACTAATGGTG AGAGATTAAA
BWF524_125  TAGAGGTGGC ACCTTTGAAT ACCCCTAGAT CATCAGCTAT GTGTGTTGCT TTTGATAATA AAATTTATGT CATTGGTGGA ACTAATGGTG AGAGATTAAA
BWF518_125  TAGAGGTGGC ACCTTTGAAT ACCCCTAGAT CATCAGCTAT GTGTGTTGCT TTTGATAATA AAATTTATGT CATTGGTGGA ACTAATGGTG AGAGATTAAA
BWF520_125  TAGAGGTGGC ACCTTTGAAT ACCCCTAGAT CATCAGCTAT GTGTGTTGCT TTTGATAATA AAATTTATGT CATTGGTGGA ACTAATGGTG AGAGATTAAA
BWF506_125  TAGAGGTGGC ACCTTTGAAT ACCCCTAGAT CATCAGCTAT GTGTGTTGCT TTCGATAATA AAATTTATGT CATTGGTGGA ACTAATGGTG AGAGATTAAA
BWF504_125  TAGAGGTGGC ACCTTTGAAT ACCCCTAGAT CATCAGCTAT GTGTGTTGCT TTTGATAATA AAATTTATGT CATTGGTGGA ACTAATGGTG AGAGATTAAA
```

BWF516_125	TAGAGGTGGC	ACCTTTGAAT	ACCCCTAGAT	CATCAGCTAT	GTGTGTTGCT	TTTGATAATA	AAATTTATGT	CATTGGTGGA	ACTAATGGTG	AGAGATTAAA
BWF503_125	TAGAGGTGGC	ACCTTTGAAT	ACCCCTAGAT	CATCAGCTAT	GTGTGTTGCT	TTTGATAATA	AAATTTATGT	CATTGGTGGA	ACTAATGGTG	AGAGATTAAA
BWF495_125	TAGAGGTGGC	ACCTTTGAAT	ACCCCTAGAT	CATCAGCTAT	GTGTGTTGCT	TTTGATAATA	AAATTTATGT	CATTGGTGGA	ACTAATGGTG	AGAGATTAAA
BWF515_125	TAGAGGTGGC	ACCTTTGAAT	ACCCCTAGAT	CATCAGCTAT	GTGTGTTGCT	TTTGATAATA	AAATTTATGT	CATTGGTGGA	ACTAATGGTG	AGAGATTAAA
BWF505_125	TAGAGGTGGC	ACCTTTGAAT	ACCCCTAGAT	CATCAGCTAT	GTGTGTTGCT	TTCGATAATA	AAATTTATGT	CATTGGTGGA	ACTAATGGTG	AGAGATTAAA
BWF509_125	TAGAGGTGGC	ACCTTTGAAT	ACCCCTAGAT	CATCAGCTAT	GTGTGTTGCT	TTTGATAATA	AAATTTATGT	CATTGGTGGA	ACTAATGGTG	AGAGATTAAA
BWF507_125	TAGAGGTGGC	ACCTTTGAAT	ACCCCTAGAT	CATCAGCTAT	GTGTGTTGCT	TTTGATAATA	AAATTTATGT	CATTGGTGGA	ACTAATGGTG	AGAGATTAAA
BWF497_125	TAGAGGTGGC	ACCTTTGAAT	ACCCCTAGAT	CATCAGCTAT	GTGTGTTGCT	TTTGATAATA	AAATTTATGT	CATTGGTGGA	ACTAATGGTG	AGAGATTAAA
BWF512_125	TAGAGGTGGC	ACCTTTGAAT	ACCCCTAGAT	CATCAGCTAT	GTGTGTTGCT	TTTGATAATA	AAATTTATGT	CATTGGTGGA	ACTAATGGTG	AGAGATTAAA
BWF510_125	TAGAGGTGGC	ACCTTTGAAT	ACCCCTAGAT	CATCAGCTAT	GTGTGTTGCT	TTTGATAATA	AAATTTATGT	CATTGGTGGA	ACTAATGGTG	AGAGATTAAA
BWF519_125	TAGAGGTGGC	ACCTTTGAAT	ACCCCTAGAT	CATCAGCTAT	GTGTGTTGCT	TTTGATAATA	AAATTTATGT	CATTGGTGGA	ACTAATGGTG	AGAGATTAAA
BWF498_125	TAGAGGTGGC	ACCTTTGAAT	ACCCCTAGAT	CATCAGCTAT	GTGTGTTGCT	TTTGATAATA	AAATTTATGT	CATTGGTGGA	ACTAATGGTG	AGAGATTAAA
BWF508_125	TAGAGGTGGC	ACCTTTGAAT	ACCCCTAGAT	CATCAGCTAT	GTGTGTTGCT	TTTGATAATA	AAATTTATGT	CATTGGTGGA	ACTAATGGTG	AGAGATTAAA
BWF493_125	TAGAGGTGGC	ACCTTTGAAT	ACCCCTAGAT	CATCAGCTAT	GTGTGTTGCT	TTTGATAATA	AAATTTATGT	CATTGGTGGA	ACTAATGGTG	AGAGATTAAA
BWF496_125	TAGAGGTGGC	ACCTTTGAAT	ACCCCTAGAT	CATCAGCTAT	GTGTGTTGCT	TTTGATAATA	AAATTTATGT	CATTGGTGGA	ACTAATGGTG	AGAGATTAAA
BWF514_125	TAGAGGTGGC	ACCTTTGAAT	ACCCCTAGAT	CATCAGCTAT	GTGTGTTGCT	TTTGATAATA	AAATTTATGT	CATTGGTGGA	ACTAATGGTG	AGAGATTAAA
CBE463	TAGAGGTGGC	ACCTTTGAAT	ACCCCTAGAT	CATCAGCTAT	GTGTGTTGCT	TTTGATAATA	AAATTTATGT	CATTGGTGGA	ACTAATGGTG	AGAGATTAAA
CBE464	TAGAGGTGGC	ACCTTTGAAT	ACCCCTAGAT	CATCAGCTAT	GTGTGTTGCT	TTTGATAATA	AAATTTATGT	CATTGGTGGA	ACTAATGGTG	AGAGATTAAA
CBE467	TAGAGGTGGC	ACCTTTGAAT	ACCCCTAGAT	CATCAGCTAT	GTGTGTTGCT	TTTGATAATA	AAATTTATGT	CATTGGTGGA	ACTAATGGTG	AGAGATTAAA
CBE468	TAGAGGTGGC	ACCTTTGAAT	ACCCCTAGAT	CATCAGCTAT	GTGTGTTGCT	TTTGATAATA	AAATTTATGT	CATTGGTGGA	ACTAATGGTG	AGAGATTAAA
BWF500_125	TAGAGGTGGC	ACCTTTGAAT	ACCCCTAGAT	CATCAGCTAT	GTGTGTTGCT	TTTGATAATA	AAATTTATGT	CATTGGTGGA	ACTAATGGTG	AGAGATTAAA
BWF522_125	TAGAGGTGGC	ACCTTTGAAT	ACCCCTAGAT	CATCAGCTAT	GTGTGTTGCT	TTTGATAATA	AAATTTATGT	CATTGGTGGA	ACTAATGGTG	AGAGATTAAA
CBE465_17	TAGAGGTGGC	ACCTTTGAAT	ACCCCTAGAT	CATCAGCTAT	GTGTGTTGCT	TTCGATAATA	AAATTTATGT	CATTGGTGGA	ACTAATGGTG	AGAGATTAAC

	1801									1900
3D7	TTCTATTGAA	GTATATGAAG	AAAAAATGAA	TAAATGGGAA	CAATTTCCAT	ATGCCTTATT	AGAAGCTAGA	AGTTCAGGAG	CAGCTTTTAA	TTACCTTAAT
CBE466_	TTCTATTGAA	GTATATGAAG	AAAAAATGAA	TAAATGGGAA	CAATTTCCAT	ATGCCTTATT	AGAAGCTAGA	AGTTCAGGAG	CAGCTTTTAA	TTACCTTAAT
BWF511_125	TTCTATTGAA	GTATATGAAG	AAAAAATGAA	TAAATGGGAA	CAATTTCCAT	ATGCCTTATT	AGAAGCTAGA	AGTTCAGGAG	CAGCTTTTAA	TTACCTTAAT
BWF513_125	TTCTATTGAA	GTATATGAAG	AAAAAATGAA	TAAATGGGAA	CAATTTCCAT	ATGCCTTATT	AGAAGCTAGA	AGTTCAGGAG	CAGCTTTTAA	TTACCTTAAT
BWF499_125	TTCTATTGAA	GTATATGAAG	AAAAAATGAA	TAAATGGGAA	CAATTTCCAT	ATGCCTTATT	AGAAGCTAGA	AGTTCAGGAG	CAGCTTTTAA	TTACCTTAAT
BWF502_125	TTCTATTGAA	GTATATGAAG	AAAAAATGAA	TAAATGGGAA	CAATTTCCAT	ATGCCTTATT	AGAAGCTAGA	AGTTCAGGAG	CAGCTTTTAA	TTACCTTAAT
BWF524_125	TTCTATTGAA	GTATATGAAG	AAAAAATGAA	TAAATGGGAA	CAATTTCCAT	ATGCCTTATT	AGAAGCTAGA	AGTTCAGGAG	CAGCTTTTAA	TTACCTTAAT
BWF518_125	TTCTATTGAA	GTATATGAAG	AAAAAATGAA	TAAATGGGAA	CAATTTCCAT	ATGCCTTATT	AGAAGCTAGA	AGTTCAGGAG	CAGCTTTTAA	TTACCTTAAT
BWF520_125	TTCTATTGAA	GTATATGAAG	AAAAAATGAA	TAAATGGGAA	CAATTTCCAT	ATGCCTTATT	AGAAGCTAGA	AGTTCAGGAG	CAGCTTTTAA	TTACCTTAAT
BWF506_125	TTCTATTGAA	GTATATGAAG	AAAAAATGAA	TAAATGGGAA	CAATTTCCAT	ATGCCTTATT	AGAAGCTAGA	AGTTCAGGAG	CAGCTTTTAA	TTACCTTAAT
BWF504_125	TTCTATTGAA	GTATATGAAG	AAAAAATGAA	TAAATGGGAA	CAATTTCCAT	ATGCCTTATT	AGAAGCTAGA	AGTTCAGGAG	CAGCTTTTAA	TTACCTTAAT
BWF516_125	TTCTATTGAA	GTATATGAAG	AAAAAATGAA	TAAATGGGAA	CAATTTCCAT	ATGCCTTATT	AGAAGCTAGA	AGTTCAGGAG	CAGCTTTTAA	TTACCTTAAT
BWF503_125	TTCTATTGAA	GTATATGAAG	AAAAAATGAA	TAAATGGGAA	CAATTTCCAT	ATGCCTTATT	AGAAGCTAGA	AGTTCAGGAG	CAGCTTTTAA	TTACCTTAAT
BWF495_125	TTCTATTGAA	GTATATGAAG	AAAAAATGAA	TAAATGGGAA	CAATTTCCAT	ATGCCTTATT	AGAAGCTAGA	AGTTCAGGAG	CAGCTTTTAA	TTACCTTAAT
BWF515_125	TTCTATTGAA	GTATATGAAG	AAAAAATGAA	TAAATGGGAA	CAATTTCCAT	ATGCCTTATT	AGAAGCTAGA	AGTTCAGGAG	CAGCTTTTAA	TTACCTTAAT
BWF505_125	TTCTATTGAA	GTATATGAAG	AAAAAATGAA	TAAATGGGAA	CAATTTCCAT	ATGCCTTATT	AGAAGCTAGA	AGTTCAGGAG	CAGCTTTTAA	TTACCTTAAT

```
BWF509_125  TTCTATTGAA GTATATGAAG AAAAAATGAA TAAATGGGAA CAATTTCCAT ATGCCTTATT AGAAGCTAGA AGTTCAGGAG CAGCTTTTAA TTACCTTAAT
BWF507_125  TTCTATTGAA GTATATGAAG AAAAAATGAA TAAATGGGAA CAATTTCCAT ATGCCTTATT AGAAGCTAGA AGTTCAGGAG CAGCTTTTAA TTACCTTAAT
BWF497_125  TTCTATTGAA GTATATGAAG AAAAAATGAA TAAATGGGAA CAATTTCCAT ATGCCTTATT AGAAGCTAGA AGTTCAGGAG CAGCTTTTAA TTACCTTAAT
BWF512_125  TTCTATTGAA GTATATGAAG AAAAAATGAA TAAATGGGAA CAATTTCCAT ATGCCTTATT AGAAGCTAGA AGTTCAGGAG CAGCTTTTAA TTACCTTAAT
BWF510_125  TTCTATTGAA GTATATGAAG AAAAAATGAA TAAATGGGAA CAATTTCCAT ATGCCTTATT AGAAGCTAGA AGTTCAGGAG CAGCTTTTAA TTACCTTAAT
BWF519_125  TTCTATTGAA GTATATGAAG AAAAAATGAA TAAATGGGAA CAATTTCCAT ATGCCTTATT AGAAGCTAGA AGTTCAGGAG CAGCTTTTAA TTACCTTAAT
BWF498_125  TTCTATTGAA GTATATGAAG AAAAAATGAA TAAATGGGAA CAATTTCCAT ATGCCTTATT AGAAGCTAGA AGTTCAGGAG CAGCTTTTAA TTACCTTAAT
BWF508_125  TTCTATTGAA GTATATGAAG AAAAAATGAA TAAATGGGAA CAATTTCCAT ATGCCTTATT AGAAGCTAGA AGTTCAGGAG CAGCTTTTAA TTACCTTAAT
BWF493_125  TTCTATTGAA GTATATGAAG AAAAAATGAA TAAATGGGAA CAATTTCCAT ATGCCTTATT AGAAGCTAGA AGTTCAGGAG CAGCTTTTAA TTACCTTAAT
BWF496_125  TTCTATTGAA GTATATGAAG AAAAAATGAA TAAATGGGAA CAATTTCCAT ATGCCTTATT AGAAGCTAGA AGTTCAGGAG CAGCTTTTAA TTACCTTAAT
BWF514_125  TTCTATTGAA GTATATGAAG AAAAAATGAA TAAATGGGAA CAATTTCCAT ATGCCTTATT AGAAGCTAGA AGTTCAGGAG CAGCTTTTAA TTACCTTAAT
    CBE463  TTCTATTGAA GTATATGAAG AAAAAATGAA TAAATGGGAA CAATTTCCAT ATGCCTTATT AGAAGCTAGA AGTTCAGGAG CAGCTTTTAA TTACCTTAAT
    CBE464  TTCTATTGAA GTATATGAAG AAAAAATGAA TAAATGGGAA CAATTTCCAT ATTCCTTATT AGAAGCTAGA AGTTCAGGAG CAGCTTTTAA TTACCTTAAT
    CBE467  TTCTATTGAA GTATATGAAG AAAAAATGAA TAAATGGGAA CAATTTCCAT ATGCCTTATT AGAAGCTAGA AGTTCAGGAG CAGCTTTTAA TTACCTTAAT
    CBE468  TTCTATTGAA GTATATGAAG AAAAAATGAA TAAATGGGAA CAATTTCCAT ATGCCTTATT AGAAGCTAGA AGTTCAGGAG CAGCTTTTAA TTACCTTAAT
BWF500_125  TTCTATTGAA GTATATGAAG AAAAAATGAA TAAATGGGAA CAATTTCCAT ATGCCTTATT AGAAGCTAGA AGTTCAGGAG CAGCTTTTAA TTACCTTAAT
BWF522_125  TTCTATTGAA GTATATGAAG AAAAAATGAA TAAATGGGAA CAATTTCCAT ATGCCTTATT AGAAGCTAGA AGTTCAGGAG CAGCTTTTAA TTACCTTAAT
 CBE465_17  TTCTATCG

            1901                                                                                                  2000
       3D7  CAAATATATG TTGTTGGAGG TATTGATAAT GAACATAACA TATTAGATTC CGTTGAACAA TATCAACCAT TTAATAAAAG ATGGCAATTT CTAAATGGTG
   CBE466_  CAAATATATG TTGTTGGAGG TATTGATAAT GAACATAACA TATTAGATTC CGTTGAACAA TATCAACCAT TTAATAAAAG ATGGCAATTT CTAAATGGTG
BWF511_125  CAAATATATG TTGTTGGAGG TATTGATAAT GAACATAACA TATTAGATTC CGTTGAACAA TATCAACCAT TTAATAAAAG ATGGCAATTT CTAAATGGTG
BWF513_125  CAAATATATG TTGTTGGAGG TATTGATAAT GAACATAACA TATTAGATTC CGTTGAACAA TATCAACCAT TTAATAAAAG ATGGCAATTT CTAAATGGTG
BWF499_125  CAAATATATG TTGTTGGAGG TATTGATAAT GAACATAACA TATTAGATTC CGTTGAACAA TATCAACCAT TTAATAAAAG ATGGCAATTT CTAAATGGTG
BWF502_125  CAAATATATG TTGTTGGAGG TATTGATAAT GAACATAACA TATTAGATTC CGTTGAACAA TATCAACCAT TTAATAAAAG ATGGCAATTT CTAAATGGTG
BWF524_125  CAAATATATG TTGTTGGAGG TATTGATAAT GAACATAACA TATTAGATTC CGTTGAACAA TATCAACCAT TTAATAAAAG ATGGCAATTT CTAAATGGTG
BWF518_125  CAAATATATG TTGTTGGAGG TATTGATAAT GAACATAACA TATTAGATTC CGTTGAACAA TATCAACCAT TTAATAAAAG ATGGCAATTT CTAAATGGTG
BWF520_125  CAAATATATG TTGTTGGAGG TATTGATAAT GAACATAACA TATTAGATTC CGTTGAACAA TATCAACCAT TTAATAAAAG ATGGCAATTT CTAAATGGTG
BWF506_125  CAAATATATG TTGTTGGAGG TATTGATAAT GAACATAACA TATTAGATTC CGTTGAACAA TATCAACCAT TTAATAAAAG ATGGCAATTT CTAAATGGTG
BWF504_125  CAAATATATG TTGTTGGAGG TATTGATAAT GAACATAACA TATTAGATTC CGTTGAACAA TATCAACCAT TTAATAAAAG ATGGCAATTT CTAAATGGTG
BWF516_125  CAAATATATG TTGTTGGAGG TATTGATAAT GAACATAACA TATTAGATTC CGTTGAACAA TATCAACCAT TTAATAAAAG ATGGCAATTT CTAAATGGTG
BWF503_125  CAAATATATG TTGTTGGAGG TATTGATAAT GAACATAACA TATTAGATTC CGTTGAACAA TATCAACCAT TTAATAAAAG ATGGCAATTT CTAAATGGTG
BWF495_125  CAAATATATG TTGTTGGAGG TATTGATAAT GAACATAACA TATTAGATTC CGTTGAACAA TATCAACCAT TTAATAAAAG ATGGCAATTT CTAAATGGTG
BWF515_125  CAAATATATG TTGTTGGAGG TATTGATAAT GAACATAACA TATTAGATTC CGTTGAACAA TATCAACCAT TTAATAAAAG ATGGCAATTT CTAAATGGTG
BWF505_125  CAAATATATG TTGTTGGAGG TATTGATAAT GAACATAACA TATTAGATTC CGTTGAACAA TATCAACCAT TTAATAAAAG ATGGCAATTT CTAAATGGTG
BWF509_125  CAAATATATG TTGTTGGAGG TATTGATAAT GAACATAACA TATTAGATTC CGTTGAACAA TATCAACCAT TTAATAAAAG ATGGCAATTT CTAAATGGTG
BWF507_125  CAAATATATG TTGTTGGAGG TATTGATAAT GAACATAACA TATTAGATTC CGTTGAACAA TATCAACCAT TTAATAAAAG ATGGCAATTT CTAAATGGTG
BWF497_125  CAAATATATG TTGTTGGAGG TATTGATAAT GAACATAACA TATTAGATTC CGTTGAACAA TATCAACCAT TTAATAAAAG ATGGCAATTT CTAAATGGTG
BWF512_125  CAAATATATG TTGTTGGAGG TATTGATAAT GAACATAACA TATTAGATTC CGTTGAACAA TATCAACCAT TTAATAAAAG ATGGCAATTT CTAAATGGTG
BWF510_125  CAAATATATG TTGTTGGAGG TATTGATAAT GAACATAACA TATTAGATTC CGTTGAACAA TATCAACCAT TTAATAAAAG ATGGCAATTT CTAAATGGTG
```

```
BWF519_125  CAAATATATG TTGTTGGAGG TATTGATAAT GAACATAACA TATTAGATTC CGTTGAACAA TATCAACCAT TTAATAAAAG ATGGCAATTT CTAAATGGTG
BWF498_125  CAAATATATG TTGTTGGAGG TATTGATAAT GAACATAACA TATTAGATTC CGTTGAACAA TATCAACCAT TTAATAAAAG ATGGCGATTT CTAAATGGTG
BWF508_125  CAAATATATG TTGTTGGAGG TATTGATAAT GAACATAACA TATTAGATTC CGTTGAACAA TATCAACCAT TTAATAAAAG ATGGCAATTT CTAAATGGTG
BWF493_125  CAAATATATG TTGTTGGAGG TATTGATAAT GAACATAACA TATTAGATTC CGTTGAACAA TATCAACCAT TTAATAAAAG ATGGCAATTT CTAAATGGTG
BWF496_125  CAAATATATG TTGTTGGAGG TATTGATAAT GAACATAACA TATTAGATTC CGTTGAACAA TATCAACCAT TTAATAAAAG ATGGCAATTT CTAAATGGTG
BWF514_125  CAAATATATG TTGTTGGAGG TATTGATAAT GAACATAACA TATTAGATTC CGTTGAACAA TATCAACCAT TTAATAAAAG ATGGCAATTT CTAAATGGTG
    CBE463  CAAATATATG TTGTTGGAGG TATTGATAAT GAACATAACA TATTAGATTC CGTTGAACAA TATCAACCAT TTAATAAAAG ATGGCAATTT CTAAATGGTG
    CBE464  CAAATATATG TTGTTGGAGG TATTGATAAT GAACATAACA TATTAGATTC CGTTGAACAA TATCAACCAT TTAATAATAG ATGGCAATTT CTAAATGGTG
    CBE467  CAAATATATG TTGTTGGAGG TATTGATAAT GAACATAACA TATTAGATTC CGTTGAACAA TATCAACCAT TTAATAAAAG ATGGCAATTT CTAAATGGTG
    CBE468  CAAATATATG TTGTTGGAGG TATTGATAAT GAACATAACA TATTAGATTC CGTTGAACAA TATCAACCAT TTAATAAAAG ATGGCAATTT CTAAATGGTG
BWF500_125  CAAATATATG TTGTTGGAGG TATTGATAAT GAACATAACA TATTAGATTC CGTTGAACAA TATCAACCAT TTAATAAAAG ATGGCAATTT CTAAATGGTG
BWF522_125  CTAATATATG CTGTAG
 CBE465_17
```

Senegal Sequences K13
2016

```
            1401                                                                                                       1500
       3D7  ACAATGCTGG CGTATGTGTA CACCTATGTC TACCAAAAAA GCTTATTTTG GAAGTGCTGT ATTGAATAAT TTCTTATACG TTTTTGGTGG TAATAACTAT
BWF535_125  ACAATGCTGG CGTATGTGTA CACCTATGTC TACCAAAAAA GCTTATTTTG GAAGTGCTGT ATTGAATAAT TTTTTATACG TTTTTGGTGG TAATAACTAT

            1501                                                                                                       1600
       3D7  GATTATAAGG CTTTATTTGA AACTGAGGTG TATGATCGTT TAAGAGATGT ATGGTATGTT TCAAGTAATT TAAATATACC TAGAAGAAAT AATTGTGGTG
BWF535_125  GATTATAAGG CTTTATTTGA AACTGAGGTG TATGATCGTT TAAGAGATGT ATGGTATGTT TCAAGTAATT TAAATATACC TAGAAGAAAT AATTGTGGTG

            1601                                                                                                       1700
       3D7  TTACGTCAAA TGGTAGAATT TATTGTATTG GGGGATATGA TGGCTCTTCT ATTATACCGA ATGTAGAAGC ATATGATCAT CGTATGAAAG CATGGGTAGA
BWF535_125  TTACGTCAAA TGGTAGAATT TATTGTATTG GGGGATATGA TGGCTCTTCT ATTATACCGA ATGTAGAAGC ATATGATCAT CGTATGAAAG CATGGGTAGA

            1701                                                                                                       1800
       3D7  GGTGGCACCT TTGAATACCC CTAGATCATC AGCTATGTGT GTTGCTTTTG ATAATAAAAT TTATGTCATT GGTGGAACTA ATGGTGAGAG ATTAAATTCT
BWF535_125  GGTGGCACCT TTGAATACCC CTAGATCATC AGCTATGTGT GTTGCTTTTG ATAATAAAAT TTATGTCATT GGTGGAACTA ATGGTGAGAG ATTAAATTCT

            1801                                                                                                       1900
       3D7  ATTGAAGTAT ATGAAGAAAA AATGAATAAA TGGGAACAAT TTCCATATGC CTTATTAGAA GCTAGAAGTT CAGGAGCAGC TTTTAATTAC CTTAATCAAA
BWF535_125  ATTGAAGTAT ATGAAGAAAA AATGAATAAA TGGGAACAAT TTCCATATGC CTTATTAGAA GCTAGAAGTT CAGGAGCAGC TTTTAATTAC CTTAATCAAA

            1901                                                                                                       2000
       3D7  TATATGTTGT TGGAGGTATT GATAATGAAC ATAACATATT AGATTCCGTT GAACAATATC AACCATTTAA TAAAAGATGG CAATTTCTAA ATGGTGTACC
BWF535_125  TATATGTTGT TGGAGGTATT GATAATGAAC ATAACATATT AGATTCCGTT GAACAATATC AACCATTTAA TAAAAGATGG CAATTTCTAA ATGGTGTACC
```

```
            1401                                                                                                     1500
       3D7  TCAACAATGC TGGCGTATGT GTACACCTAT GTCTACCAAA AAAGCTTATT TTGGAAGTGC TGTATTGAAT AATTTCTTAT ACGTTTTTGG TGGTAATAAC
   CBE467_  TCAACAATGC TGGCGTATGT GTACACCTAT GTCTACCAAA AAAGCTTATT TTGGAAGTGC TGTATTGAAT AATTTCTTAT ACGTTTTTGG TGGTAATAAC
BWF530_125  TCAACAATGC TGGCGTATGT GTACACCTAT GTCTACCAAA AAAGCTTATT TTGGAAGTGC TGTATTGAAT AATTTCTTAT ACGTTTTTGG TGGTAATAAC
BWF531_125  TCAACAATGC TGGCGTATGT GTACACCTAT GTCTACCAAA AAAGCTTATT TTGGAAGTGC TGTATTGAAT AATTTCTTAT ACGTTTTTGG TGGTAATAAC
BWF532_125  TCAACAATGC TGGCGTATGT GTACACCTAT GTCTACCAAA AAAGCTTATT TTGGAAGTGC TGTATTGAAT AATTTCTTAT ACGTTTTTGG TGGTAATAAC
BWF533_125  TCAACAATGC TGGCGTATGT GTACACCTAT GTCTACCAAA AAAGCTTATT TTGGAAGTGC TGTATTGAAT AATTTCTTAT ACGTTTTTGG TGGTAATAAC
BWF534_125  TCAACAATGC TGGCGTATGT GTACACCTAT GTCTACCAAA AAAGCTTATT TTGGAAGTGC TGTATTGAAT AATTTCTTAT ACGTTTTTGG TGGTAATAAC
BWF536_125  TCAACAATGC TGGCGTATGT GTACACCTAT GTCTACCAAA AAAGCTTATT TTGGAAGTGC TGTATTGAAT AATTTCTTAT ACGTTTTTGG TGGTAATAAC
BWF537_125  TCAACAATGC TGGCGTATGT GTACACCTAT GTCTACCAAA AAAGCTTATT TTGGAAGTGC TGTATTGAAT AATTTCTTAT ACGTTTTTGG TGGTAATAAC
BWF538_125  TCAACAATGC TGGCGTATGT GTACACCTAT GTCTACCAAA AAAGCTTATT TTGGAAGTGC TGTATTGAAT AATTTCTTAT ACGTTTTTGG TGGTAATAAC
BWF539_125  TCAACAATGC TGGCGTATGT GTACACCTAT GTCTACCAAA AAAGCTTATT TTGGAAGTGC TGTATTGAAT AATTTCTTAT ACGTTTTTGG TGGTAATAAC
BWF540_125  TCAACAATGC TGGCGTATGT GTACACCTAT GTCTACCAAA AAAGCTTATT TTGGAAGTGC TGTATTGAAT AATTTCTTAT ACGTTTTTGG TGGTAATAAC
BWF541_125  TCAACAATGC TGGCGTATGT GTACACCTAT GTCTACCAAA AAAGCTTATT TTGGAAGTGC TGTATTGAAT AATTTCTTAT ACGTTTTTGG TGGTAATAAC
BWF442_125  TCAACAATGC TGGCGTATGT GTACACCTAT GTCTACCAAA AAAGCTTATT TTGGAAGTGC TGTATTGAAT AATTTCTTAT ACGTTTTTGG TGGTAATAAC
BWF543_125  TCAACAATGC TGGCGTATGT GTACACCTAT GTCTACCAAA AAAGCTTATT TTGGAAGTGC TGTATTGAAT AATTTCTTAT ACGTTTTTGG TGGTAATAAC
BWF544_125  TCAACAATGC TGGCGTATGT GTACACCTAT GTCTACCAAA AAAGCTTATT TTGGAAGTGC TGTATTGAAT AATTTCTTAT ACGTTTTTGG TGGTAATAAC
BWF545_125  TCAACAATGC TGGCGTATGT GTACACCTAT GTCTACCAAA AAAGCTTATT TTGGAAGTGC TGTATTGAAT AATTTCTTAT ACGTTTTTGG TGGTAATAAC
BWF546_125  TCAACAATGC TGGCGTATGT GTACACCTAT GTCTACCAAA AAAGCTTATT TTGGAAGTGC TGTATTGAAT AATTTCTTAT ACGTTTTTGG TGGTAATAAC
BWF547_125  TCAACAATGC TGGCGTATGT GTACACCTAT GTCTACCAAA AAAGCTTATT TTGGAAGTGC TGTATTGAAT AATTTCTTAT ACGTTTTTGG TGGTAATAAC
BWF548_125  TCAACAATGC TGGCGTATGT GTACACCTAT GTCTACCAAA AAAGCTTATT TTGGAAGTGC TGTATTGAAT AATTTCTTAT ACGTTTTTGG TGGTAATAAC
BWF549_125  TCAACAATGC TGGCGTATGT GTACACCTAT GTCTACCAAA AAAGCTTATT TTGGAAGTGC TGTATTGAAT AATTTCTTAT ACGTTTTTGG TGGTAATAAC
BWF550_125  TCAACAATGC TGGCGTATGT GTACACCTAT GTCTACCAAA AAAGCTTATT TTGGAAGTGC TGTATTGAAT AATTTCTTAT ACGTTTTTGG TGGTAATAAC
BWF451_125  TCAACAATGC TGGCGTATGT GTACACCTAT GTCTACCAAA AAAGCTTATT TTGGAAGTGC TGTATTGAAT AATTTCTTAT ACGTTTTTGG TGGTAATAAC
BWF552_125  TCAACAATGC TGGCGTATGT GTACACCTAT GTCTACCAAA AAAGCTTATT TTGGAAGTGC TGTATTGAAT AATTTCTTAT ACGTTTTTGG TGGTAATAAC
BWF553_125  TCAACAATGC TGGCGTATGT GTACACCTAT GTCTACCAAA AAAGCTTATT TTGGAAGTGC TGTATTGAAT AATTTCTTAT ACGTTTTTGG TGGTAATAAC
BWF554_125  TCAACAATGC TGGCGTATGT GTACACCTAT GTCTACCAAA AAAGCTTATT TTGGAAGTGC TGTATTGAAT AATTTCTTAT ACGTTTTTGG TGGTAATAAC
BWF555_125  TCAACAATGC TGGCGTATGT GTACACCTAT GTCTACCAAA AAAGCTTATT TTGGAAGTGC TGTATTGAAT AATTTCTTAT ACGTTTTTGG TGGTAATAAC
BWF556_125  TCAACAATGC TGGCGTATGT GTACACCTAT GTCTACCAAA AAAGCTTATT TTGGAAGTGC TGTATTGAAT AATTTCTTAT ACGTTTTTGG TGGTAATAAC
BWF557_125  TCAACAATGC TGGCGTATGT GTACACCTAT GTCTACCAAA AAAGCTTATT TTGGAAGTGC TGTATTGAAT AATTTCTTAT ACGTTTTTGG TGGTAATAAC
BWF558_125  TCAACAATGC TGGCGTATGT GTACACCTAT GTCTACCAAA AAAGCTTATT TTGGAAGTGC TGTATTGAAT AATTTCTTAT ACGTTTTTGG TGGTAATAAC
BWF559_125  TCAACAATGC TGGCGTATGT GTACACCTAT GTCTACCAAA AAAGCTTATT TTGGAAGTGC TGTATTGAAT AATTTCTTAT ACGTTTTTGG TGGTAATAAC
BWF560_125  TCAACAATGC TGGCGTATGT GTACACCTAT GTCTACCAAA AAAGCTTATT TTGGAAGTGC TGTATTGAAT AATTTCTTAT ACGTTTTTGG TGGTAATAAC
BWF561_125  TCAACAATGC TGGCGTATGT GTACACCTAT GTCTACCAAA AAAGCTTATT TTGGAAGTGC TGTATTGAAT AATTTCTTAT ACGTTTTTGG TGGTAATAAC

            1501                                                                                                     1600
       3D7  TATGATTATA AGGCTTTATT TGAAACTGAG GTGTATGATC GTTTAAGAGA TGTATGGTAT GTTTCAAGTA ATTTAAATAT ACCTAGAAGA AATAATTGTG
   CBE467_  TATGATTATA AGGCTTTATT TGAAACTGAG GTGTATGATC GTTTAAGAGA TGTATGGTAT GTTTCAAGTA ATTTAAATAT ACCTAGAAGA AATAATTGTG
BWF530_125  TATGATTATA AGGCTTTATT TGAAACTGAG GTGTATGATC GTTTAAGAGA TGTATGGTAT GTTTCAAGTA ATTTAAATAT ACCTAGAAGA AATAATTGTG
```

```
BWF531_125  TATGATTATA AGGCTTTATT TGAAACTGAG GTGTATGATC GTTTAAGAGA TGTATGGTAT GTTTCAAGTA ATTTAAATAT ACCTAGAAGA AATAATTGTG
BWF532_125  TATGATTATA AGGCTTTATT TGAAACTGAG GTGTATGATC GTTTAAGAGA TGTATGGTAT GTTTCAAGTA ATTTAAATAT ACCTAGAAGA AATAATTGTG
BWF533_125  TATGATTATA AGGCTTTATT TGAAACTGAG GTGTATGATC GTTTAAGAGA TGTATGGTAT GTTTCAAGTA ATTTAAATAT ACCTAGAAGA AATAATTGTG
BWF534_125  TATGATTATA AGGCTTTATT TGAAACTGAG GTGTATGATC GTTTAAGAGA TGTATGGTAT GTTTCAAGTA ATTTAAATAT ACCTAGAAGA AATAATTGTG
BWF536_125  TATGATTATA AGGCTTTATT TGAAACTGAG GTGTATGATC GTTTAAGAGA TGTATGGTAT GTTTCAAGTA ATTTAAATAT ACCTAGAAGA AATAATTGTG
BWF537_125  TATGATTATA AGGCTTTATT TGAAACTGAG GTGTATGATC GTTTAAGAGA TGTATGGTAT GTTTCAAGTA ATTTAAATAT ACCTAGAAGA AATAATTGTG
BWF538_125  TATGATTATA AGGCTTTATT TGAAACTGAG GTGTATGATC GTTTAAGAGA TGTATGGTAT GTTTCAAGTA ATTTAAATAT ACCTAGAAGA AATAATTGTG
BWF539_125  TATGATTATA AGGCTTTATT TGAAACTGAG GTGTATGATC GTTTAAGAGA TGTATGGTAT GTTTCAAGTA ATTTAAATAT ACCTAGAAGA AATAATTGTG
BWF540_125  TATGATTATA AGGCTTTATT TGAAACTGAG GTGTATGATC GTTTAAGAGA TGTATGGTAT GTTTCAAGTA ATTTAAATAT ACCTAGAAGA AATAATTGTG
BWF541_125  TATGATTATA AGGCTTTATT TGAAACTGAG GTGTATGATC GTTTAAGAGA TGTATGGTAT GTTTCAAGTA ATTTAAATAT ACCTAGAAGA AATAATTGTG
BWF442_125  TATGATTATA AGGCTTTATT TGAAACTGAG GTGTATGATC GTTTAAGAGA TGTATGGTAT GTTTCAAGTA ATTTAAATAT ACCTAGAAGA AATAATTGTG
BWF543_125  TATGATTATA AGGCTTTATT TGAAACTGAG GTGTATGATC GTTTAAGAGA TGTATGGTAT GTTTCAAGTA ATTTAAATAT ACCTAGAAGA AATAATTGTG
BWF544_125  TATGATTATA AGGCTTTATT TGAAACTGAG GTGTATGATC GTTTAAGAGA TGTATGGTAT GTTTCAAGTA ATTTAAATAT ACCTAGAAGA AATAATTGTG
BWF545_125  TATGATTATA AGGCTTTATT TGAAACTGAG GTGTATGATC GTTTAAGAGA TGTATGGTAT GTTTCAAGTA ATTTAAATAT ACCTAGAAGA AATAATTGTG
BWF546_125  TATGATTATA AGGCTTTATT TGAAACTGAG GTGTATGATC GTTTAAGAGA TGTATGGTAT GTTTCAAGTA ATTTAAATAT ACCTAGAAGA AATAATTGTG
BWF447_125  TATGATTATA AGGCTTTATT TGAAACTGAG GTGTATGATC GTTTAAGAGA TGTATGGTAT GTTTCAAGTA ATTTAAATAT ACCTAGAAGA AATAATTGTG
BWF548_125  TATGATTATA AGGCTTTATT TGAAACTGAG GTGTATGATC GTTTAAGAGA TGTATGGTAT GTTTCAAGTA ATTTAAATAT ACCTAGAAGA AATAATTGTG
BWF549_125  TATGATTATA AGGCTTTATT TGAAACTGAG GTGTATGATC GTTTAAGAGA TGTATGGTAT GTTTCAAGTA ATTTAAATAT ACCTAGAAGA AATAATTGTG
BWF550_125  TATGATTATA AGGCTTTATT TGAAACTGAG GTGTATGATC GTTTAAGAGA TGTATGGTAT GTTTCAAGTA ATTTAAATAT ACCTAGAAGA AATAATTGTG
BWF551_125  TATGATTATA AGGCTTTATT TGAAACTGAG GTGTATGATC GTTTAAGAGA TGTATGGTAT GTTTCAAGTA ATTTAAATAT ACCTAGAAGA AATAATTGTG
BWF552_125  TATGATTATA AGGCTTTATT TGAAACTGAG GTGTATGATC GTTTAAGAGA TGTATGGTAT GTTTCAAGTA ATTTAAATAT ACCTAGAAGA AATAATTGTG
BWF553_125  TATGATTATA AGGCTTTATT TGAAACTGAG GTGTATGATC GTTTAAGAGA TGTATGGTAT GTTTCAAGTA ATTTAAATAT ACCTAGAAGA AATAATTGTG
BWF554_125  TATGATTATA AGGCTTTATT TGAAACTGAG GTGTATGATC GTTTAAGAGA TGTATGGTAT GTTTCAAGTA ATTTAAATAT ACCTAGAAGA AATAATTGTG
BWF555_125  TATGATTATA AGGCTTTATT TGAAACTGAG GTGTATGATC GTTTAAGAGA TGTATGGTAT GTTTCAAGTA ATTTAAATAT ACCTAGAAGA AATAATTGTG
BWF556_125  TATGATTATA AGGCTTTATT TGAAACTGAG GTGTATGATC GTTTAAGAGA TGTATGGTAT GTTTCAAGTA ATTTAAATAT ACCTAGAAGA AATAATTGTG
BWF557_125  TATGATTATA AGGCTTTATT TGAAACTGAG GTGTATGATC GTTTAAGAGA TGTATGGTAT GTTTCAAGTA ATTTAAATAT ACCTAGAAGA AATAATTGTG
BWF558_125  TATGATTATA AGGCTTTATT TGAAACTGAG GTGTATGATC GTTTAAGAGA TGTATGGTAT GTTTCAAGTA ATTTAAATAT ACCTAGAAGA AATAATTGTG
BWF559_125  TATGATTATA AGGCTTTATT TGAAACTGAG GTGTATGATC GTTTAAGAGA TGTATGGTAT GTTTCAAGTA ATTTAAATAT ACCTAGAAGA AATAATTGTG
BWF560_125  TATGATTATA AGGCTTTATT TGAAACTGAG GTGTATGATC GTTTAAGAGA TGTATGGTAT GTTTCAAGTA ATTTAAATAT ACCTAGAAGA AATAATTGTG
BWF561_125  TATGATTATA AGGCTTTATT TGAAACTGAG GTGTATGATC GTTTAAGAGA TGTATGGTAT GTTTCAAGTA ATTTAAATAT ACCTAGAAGA AATAATTGTG

            1601                                                                                                      1700
       3D7  GTGTTACGTC AAATGGTAGA ATTTATTGTA TTGGGGGATA TGATGGCTCT TCTATTATAC CCGAATGTAG AAGCATATGA TCATCGTATG AAAGCATGGG
   CBE467_  GTGTTACGTC AAATGGTAGA ATTTATTGTA TTGGGGGATA TGATGGCTCT TCTATTATAC CCGAATGTAG AAGCATATGA TCATCGTATG AAAGCATGGG
BWF530_125  GTGTTACGTC AAATGGTAGA ATTTATTGTA TTGGGGGATA TGATGGCTCT TCTATTATAC CCGAATGTAG AAGCATATGA TCATCGTATG AAAGCATGGG
BWF531_125  GTGTTACGTC AAATGGTAGA ATTTATTGTA TTGGGGGATA TGATGGCTCT TCTATTATAC CCGAATGTAG AAGCATATGA TCATCGTATG AAAGCATGGG
BWF432_125  GTGTTACGTC AAATGGTAGA ATTTATTGTA TTGGGGGATA TGATGGCTCT TCTATTATAC CCGAATGTAG AAGCATATGA TCATCGTATG AAAGCATGGG
BWF533_125  GTGTTACGTC AAATGGTAGA ATTTATTGTA TTGGGGGATA TGATGGCTCT TCTATTATAC CCGAATGTAG AAGCATATGA TCATCGTATG AAAGCATGGG
BWF534_125  GTGTTACGTC AAATGGTAGA ATTTATTGTA TTGGGGGATA TGATGGCTCT TCTATTATAC CCGAATGTAG AAGCATATGA TCATCGTATG AAAGCATGGG
BWF536_125  GTGTTACGTC AAATGGTAGA ATTTATTGTA TTGGGGGATA TGATGGCTCT TCTATTATAC CCGAATGTAG AAGCATATGA TCATCGTATG AAAGCATGGG
BWF537_125  GTGTTACGTC AAATGGTAGA ATTTATTGTA TTGGGGGATA TGATGGCTCT TCTATTATAC CCGAATGTAG AAGCATATGA TCATCGTATG AAAGCATGGG
```

```
BWF538_125  GTGTTACGTC AAATGGTAGA ATTTATTGTA TTGGGGGATA TGATGGCTCT TCTATTATAC CCGAATGTAG AAGCATATGA TCATCGTATG AAAGCATGGG
BWF539_125  GTGTTACGTC AAATGGTAGA ATTTATTGTA TTGGGGGATA TGATGGCTCT TCTATTATAC CCGAATGTAG AAGCATATGA TCATCGTATG AAAGCATGGG
BWF540_125  GTGTTACGTC AAATGGTAGA ATTTATTGTA TTGGGGGATA TGATGGCTCT TCTATTATAC CCGAATGTAG AAGCATATGA TCATCGTATG AAAGCATGGG
BWF541_125  GTGTTACGTC AAATGGTAGA ATTTATTGTA TTGGGGGATA TGATGGCTCT TCTATTATAC CCGAATGTAG AAGCATATGA TCATCGTATG AAAGCATGGG
BWF442_125  GTGTTACGTC AAATGGTAGA ATTTATTGTA TTGGGGGATA TGATGGCTCT TCTATTATAC CCGAATGTAG AAGCATATGA TCATCGTATG AAAGCATGGG
BWF543_125  GTGTTACGTC AAATGGTAGA ATTTATTGTA TTGGGGGATA TGATGGCTCT TCTATTATAC CCGAATGTAG AAGCATATGA TCATCGTATG AAAGCATGGG
BWF544_125  GTGTTACGTC AAATGGTAGA ATTTATTGTA TTGGGGGATA TGATGGCTCT TCTATTATAC CCGAATGTAG AAGCATATGA TCATCGTATG AAAGCATGGG
BWF545_125  GTGTTACGTC AAATGGTAGA ATTTATTGTA TTGGGGGATA TGATGGCTCT TCTATTATAC CCGAATGTAG AAGCATATGA TCATCGTATG AAAGCATGGG
BWF546_125  GTGTTACGTC AAATGGTAGA ATTTATTGTA TTGGGGGATA TGATGGCTCT TCTATTATAC CCGAATGTAG AAGCATATGA TCATCGTATG AAAGCATGGG
BWF447_125  GTGTTACGTC AAATGGTAGA ATTTATTGTA TTGGGGGATA TGATGGCTCT TCTATTATAC CCGAATGTAG AAGCATATGA TCATCGTATG AAAGCATGGG
BWF548_125  GTGTTACGTC AAATGGTAGA ATTTATTGTA TTGGGGGATA TGATGGCTCT TCTATTATAC CCGAATGTAG AAGCATATGA TCATCGTATG AAAGCATGGG
BWF549_125  GTGTTACGTC AAATGGTAGA ATTTATTGTA TTGGGGGATA TGATGGCTCT TCTATTATAC CCGAATGTAG AAGCATATGA TCATCGTATG AAAGCATGGG
BWF550_125  GTGTTACGTC AAATGGTAGA ATTTATTGTA TTGGGGGATA TGATGGCTCT TCTATTATAC CCGAATGTAG AAGCATATGA TCATCGTATG AAAGCATGGG
BWF551_125  GTGTTACGTC AAATGGTAGA ATTTATTGTA TTGGGGGATA TGATGGCTCT TCTATTATAC CCGAATGTAG AAGCATATGA TCATCGTATG AAAGCATGGG
BWF552_125  GTGTTACGTC AAATGGTAGA ATTTATTGTA TTGGGGGATA TGATGGCTCT TCTATTATAC CCGAATGTAG AAGCATATGA TCATCGTATG AAAGCATGGG
BWF453_125  GTGTTACGTC AAATGGTAGA ATTTATTGTA TTGGGGGATA TGATGGCTCT TCTATTATAC CCGAATGTAG AAGCATATGA TCATCGTATG AAAGCATGGG
BWF454_125  GTGTTACGTC AAATGGTAGA ATTTATTGTA TTGGGGGATA TGATGGCTCT TTTATTATAC CCGAATGTAG AAGCATATGA TCATCGTATG AAAGCATGGG
BWF555_125  GTGTTACGTC AAATGGTAGA ATTTATTGTA TTGGGGGATA TGATGGCTCT TCTATTATAC CCGAATGTAG AAGCATATGA TCATCGTATG AAAGCATGGG
BWF556_125  GTGTTACGTC AAATGGTAGA ATTTATTGTA TTGGGGGATA TGATGGCTCT TCTATTATAC CCGAATGTAG AAGCATATGA TCATCGTATG AAAGCATGGG
BWF557_125  GTGTTACGTC AAATGGTAGA ATTTATTGTA TTGGGGGATA TGATGGCTCT TCTATTATAC CCGAATGTAG AAGCATATGA TCATCGTATG AAAGCATGGG
BWF558_125  GTGTTACGTC AAATGGTAGA ATTTATTGTA TTGGGGGATA TGATGGCTCT TCTATTATAC CCGAATGTAG AAGCATATGA TCATCGTATG AAAGCATGGG
BWF559_125  GTGTTACGTC AAATGGTAGA ATTTATTGTA TTGGGGGATA TGATGGCTCT TCTATTATAC CCGAATGTAG AAGCATATGA TCATCGTATG AAAGCATGGG
BWF560_125  GTGTTACGTC AAATGGTAGA ATTTATTGTA TTGGGGGATA TGATGGCTCT TCTATTATAC CCGAATGTAG AAGCATATGA TCATCGTATG AAAGCATGGG
BWF561_125  GTGTTACGTC AAATGGTAGA ATTTATTGTA TTGGGGGATA TGATGGCTCT TCTATTATAC CCGAATGTAG AAGCATATGA TCATCGTATG AAAGCATGGG

            1701                                                                                                      1800
       3D7  TAGAGGTGGC ACCTTTGAAT ACCCCTAGAT CATCAGCTAT GTGTGTTGCT TTTGATAATA AAATTTATGT CATTGGTGGA ACTAATGGTG AGAGATTAAA
   CBE467_  GTGTTACGTC AAATGGTAGA ATTTATTGTA TTGGGGGATA TGATGGCTCT TCTATTATAC CCGAATGTAG AAGCATATGA TCATCGTATG AAAGCATGGG
BWF530_125  TAGAGGTGGC ACCTTTGAAT ACCCCTAGAT CATCAGCTAT GTGTGTTGCT TTTGATAATA AAATTTATGT CATTGGTGGA ACTAATGGTG AGAGATTAAA
BWF531_125  TAGAGGTGGC ACCTTTGAAT ACCCCTAGAT CATCAGCTAT GTGTGTTGCT TTTGATAATA AAATTTATGT CATTGGTGGA ACTAATGGTG AGAGATTAAA
BWF532_125  TAGAGGTGGC ACCTTTGAAT ACCCCTAGAT CATCAGCTAT GTGTGTTGCT TTTGATAATA AAATTTATGT CATTGGTGGA ACTAATGGTG AGAGATTAAA
BWF533_125  TAGAGGTGGC ACCTTTGAAT ACCCCTAGAT CATCAGCTAT GTGTGTTGCT TTTGATAATA AAATTTATGT CATTGGTGGA ACTAATGGTG AGAGATTAAA
BWF534_125  TAGAGGTGGC ACCTTTGAAT ACCCCTAGAT CATCAGCTAT GTGTGTTGCT TTTGATAATA AAATTTATGT CATTGGTGGA ACTAATGGTG AGAGATTAAA
BWF536_125  TAGAGGTGGC ACCTTTGAAT ACCCCTAGAT CATCAGCTAT GTGTGTTGCT TTTGATAATA AAATTTATGT CATTGGTGGA ACTAATGGTG AGAGATTAAA
BWF537_125  TAGAGGTGGC ACCTTTGAAT ACCCCTAGAT CATCAGCTAT GTGTGTTGCT TTTGATAATA AAATTTATGT CATTGGTGGA ACTAATGGTG AGAGATTAAA
BWF538_125  TAGAGGTGGC ACCTTTGAAT ACCCCTAGAT CATCAGCTAT GTGTGTTGCT TTTGATAATA AAATTTATGT CATTGGTGGA ACTAATGGTG AGAGATTAAA
BWF539_125  TAGAGGTGGC ACCTTTGAAT ACCCCTAGAT CATCAGCTAT GTGTGTTGCT TTTGATAATA AAATTTATGT CATTGGTGGA ACTAATGGTG AGAGATTAAA
BWF540_125  TAGAGGTGGC ACCTTTGAAT ACCCCTAGAT CATCAGCTAT GTGTGTTGCT TTTGATAATA AAATTTATGT CATTGGTGGA ACTAATGGTG AGAGATTAAA
BWF541_125  TAGAGGTGGC ACCTTTGAAT ACCCCTAGAT CATCAGCTAT GTGTGTTGCT TTTGATAATA AAATTTATGT CATTGGTGGA ACTAATGGTG AGAGATTAAA
BWF542_125  TAGAGGTGGC ACCTTTGAAT ACCCCTAGAT CATCAGCTAT GTGTGTTGCT TTTGATAATA AAATTTATGT CATTGGTGGA ACTAATGGTG AGAGATTAAA
BWF543_125  TAGAGGTGGC ACCTTTGAAT ACCCCTAGAT CATCAGCTAT GTGTGTTGCT TTTGATAATA AAATTTATGT CATTGGTGGA ACTAATGGTG AGAGATTAAA
```

```
BWF544_125  TAGAGGTGGC ACCTTTGAAT ACCCCTAGAT CATCAGCTAT GTGTGTTGCT TTTGATAATA AAATTTATGT CATTGGTGGA ACTAATGGTG AGAGATTAAA
BWF545_125  TAGAGGTGGC ACCTTTGAAT ACCCCTAGAT CATCAGCTAT GTGTGTTGCT TTTGATAATA AAATTTATGT CATTGGTGGA ACTAATGGTG AGAGATTAAA
BWF546_125  TAGAGGTGGC ACCTTTGAAT ACCCCTAGAT CATCAGCTAT GTGTGTTGCT TTTGATAATA AAATTTATGT CATTGGTGGA ACTAATGGTG AGAGATTAAA
BWF547_125  TAGAGGTGGC ACCTTTGAAT ACCCCTAGAT CATCAGCTAT GTGTGTTGCT TTTGATAATA AAATTTATGT CATTGGTGGA ACTAATGGTG AGAGATTAAA
BWF548_125  TAGAGGTGGC ACCTTTGAAT ACCCCTAGAT CATCAGCTAT GTGTGTTGCT TTTGATAATA AAATTTATGT CATTGGTGGA ACTAATGGTG AGAGATTAAA
BWF549_125  TAGAGGTGGC ACCTTTGAAT ACCCCTAGAT CATCAGCTAT GTGTGTTGCT TTTGATAATA AAATTTATGT CATTGGTGGA ACTAATGGTG AGAGATTAAA
BWF550_125  TAGAGGTGGC ACCTTTGAAT ACCCCTAGAT CATCAGCTAT GTGTGTTGCT TTTGATAATA AAATTTATGT CATTGGTGGA ACTAATGGTG AGAGATTAAA
BWF551_125  TAGAGGTGGC ACCTTTGAAT ACCCCTAGAT CATCAGCTAT GTGTGTTGCT TTTGATAATA AAATTTATGT CATTGGTGGA ACTAATGGTG AGAGATTAAA
BWF552_125  TAGAGGTGGC ACCTTTGAAT ACCCCTAGAT CATCAGCTAT GTGTGTTGCT TTTGATAATA AAATTTATGT CATTGGTGGA ACTAATGGTG AGAGATTAAA
BWF553_125  TAGAGGTGGC ACCTTTGAAT ACCCCTAGAT CATCAGCTAT GTGTGTTGCT TTTGATAATA AAATTTATGT CATTGGTGGA ACTAATGGTG AGAGATTAAA
BWF554_125  TAGAGGTGGC ACCTTTGAAT ACCCCTAGAT CATCAGCTAT GTGTGTTGCT TTTGATAATA AAATTTATGT CATTGGTGGA ACTAATGGTG AGAGATTAAA
BWF555_125  TAGAGGTGGC ACCTTTGAAT ACCCCTAGAT CATCAGCTAT GTGTGTTGCT TTTGATAATA AAATTTATGT CATTGGTGGA ACTAATGGTG AGAGATTAAA
BWF556_125  TAGAGGTGGC ACCTTTGAAT ACCCCTAGAT CATCAGCTAT GTGTGTTGCT TTTGATAATA AAATTTATGT CATTGGTGGA ACTAATGGTG AGAGATTAAA
BWF557_125  TAGAGGTGGC ACCTTTGAAT ACCCCTAGAT CATCAGCTAT GTGTGTTGCT TTTGATAATA AAATTTATGT CATTGGTGGA ACTAATGGTG AGAGATTAAA
BWF558_125  TAGAGGTGGC ACCTTTGAAT ACCCCTAGAT CATCAGCTAT GTGTGTTGCT TTTGATAATA AAATTTATGT CATTGGTGGA ACTAATGGTG AGAGATTAAA
BWF559_125  TAGAGGTGGC ACCTTTGAAT ACCCCTAGAT CATCAGCTAT GTGTGTTGCT TTTGATAATA AAATTTATGT CATTGGTGGA ACTAATGGTG AGAGATTAAA
BWF560_125  TAGAGGTGGC ACCTTTGAAT ACCCCTAGAT CATCAGCTAT GTGTGTTGCT TTTGATAATA AAATTTATGT CATTGGTGGA ACTAATGGTG AGAGATTAAA
BWF561_125  TAGAGGTGGC ACCTTTGAAT ACCCCTAGAT CATCAGCTAT GTGTGTTGCT TTTGATAATA AAATTTATGT CATTGGTGGA ACTAATGGTG AGAGATTAAA

            1801                                                                                                      1900
       3D7  TTCTATTGAA GTATATGAAG AAAAAATGAA TAAATGGGAA CAATTTCCAT ATGCCTTATT AGAAGCTAGA AGTTCAGGAG CAGCTTTTAA TTACCTTAAT
   CBE467_  TTCTATTGAA GTATATGAAG AAAAAATGAA TAAATGGGAA CAATTTCCAT ATGCCTTATT AGAAGCTAGA AGTTCAGGAG CAGCTTTTAA TTACCTTAAT
BWF530_125  TTCTATTGAA GTATATGAAG AAAAAATGAA TAAATGGGAA CAATTTCCAT ATGCCTTATT AGAAGCTAGA AGTTCAGGAG CAGCTTTTAA TTACCTTAAT
BWF531_125  TTCTATTGAA GTATATGAAG AAAAAATGAA TAAATGGGAA CAATTTCCAT ATGCCTTATT AGAAGCTAGA AGTTCAGGAG CAGCTTTTAA TTACCTTAAT
BWF532_125  TTCTATTGAA GTATATGAAG AAAAAATGAA TAAATGGGAA CAATTTCCAT ATGCCTTATT AGAAGCTAGA AGTTCAGGAG CAGCTTTTAA TTACCTTAAT
BWF533_125  TTCTATTGAA GTATATGAAG AAAAAATGAA TAAATGGGAA CAATTTCCAT ATGCCTTATT AGAAGCTAGA AGTTCAGGAG CAGCTTTTAA TTACCTTAAT
BWF534_125  TTCTATTGAA GTATATGAAG AAAAAATGAA TAAATGGGAA CAATTTCCAT ATGCCTTATT AGAAGCTAGA AGTTCAGGAG CAGCTTTTAA TTACCTTAAT
BWF536_125  TTCTATTGAA GTATATGAAG AAAAAATGAA TAAATGGGAA CAATTTCCAT ATGCCTTATT AGAAGCTAGA AGTTCAGGAG CAGCTTTTAA TTACCTTAAT
BWF537_125  TTCTATTGAA GTATATGAAG AAAAAATGAA TAAATGGGAA CAATTTCCAT ATGCCTTATT AGAAGCTAGA AGTTCAGGAG CAGCTTTTAA TTACCTTAAT
BWF538_125  TTCTATTGAA GTATATGAAG AAAAAATGAA TAAATGGGAA CAATTTCCAT ATGCCTTATT AGAAGCTAGA AGTTCAGGAG CAGCTTTTAA TTACCTTAAT
BWF539_125  TTCTATTGAA GTATATGAAG AAAAAATGAA TAAATGGGAA CAATTTCCAT ATGCCTTATT AGAAGCTAGA AGTTCAGGAG CAGCTTTTAA TTACCTTAAT
BWF540_125  TTCTATTGAA GTATATGAAG AAAAAATGAA TAAATGGGAA CAATTTCCAT ATGCCTTATT AGAAGCTAGA AGTTCAGGAG CAGCTTTTAA TTACCTTAAT
BWF541_125  TTCTATTGAA GTATATGAAG AAAAAATGAA TAAATGGGAA CAATTTCCAT ATGCCTTATT AGAAGCTAGA AGTTCAGGAG CAGCTTTTAA TTACCTTAAT
BWF442_125  TTCTATTGAA GTATATGAAG AAAAAATGAA TAAATGGGAA CAATTTCCAT ATGCCTTATT AGAAGCTAGA AGTTCAGGAG CAGCTTTTAA TTACCTTAAT
BWF543_125  TTCTATTGAA GTATATGAAG AAAAAATGAA TAAATGGGAA CAATTTCCAT ATGCCTTATT AGAAGCTAGA AGTTCAGGAG CAGCTTTTAA TTACCTTAAT
BWF544_125  TTCTATTGAA GTATATGAAG AAAAAATGAA TAAATGGGAA CAATTTCCAT ATGCCTTATT AGAAGCTAGA AGTTCAGGAG CAGCTTTTAA TTACCTTAAT
BWF545_125  TTCTATTGAA GTATATGAAG AAAAAATGAA TAAATGGGAA CAATTTCCAT ATGCCTTATT AGAAGCTAGA AGTTCAGGAG CAGCTTTTAA TTACCTTAAT
BWF546_125  TTCTATTGAA GTATATGAAG AAAAAATGAA TAAATGGGAA CAATTTCCAT ATGCCTTATT AGAAGCTAGA AGTTCAGGAG CAGCTTTTAA TTACCTTAAT
BWF447_125  TTCTATTGAA GTATATGAAG AAAAAATGAA TAAATGGGAA CAATTTCCAT ATGCCTTATT AGAAGCTAGA AGTTCAGGAG CAGCTTTTAA TTACCTTAAT
BWF548_125  TTCTATTGAA GTATATGAAG AAAAAATGAA TAAATGGGAA CAATTTCCAT ATGCCTTATT AGAAGCTAGA AGTTCAGGAG CAGCTTTTAA TTACCTTAAT
BWF549_125  TTCTATTGAA GTATATGAAG AAAAAATGAA TAAATGGGAA CAATTTCCAT ATGCCTTATT AGAAGCTAGA AGTTCAGGAG CAGCTTTTAA TTACCTTAAT
```

```
BWF550_125  TTCTATTGAA GTATATGAAG AAAAAATGAA TAAATGGGAA CAATTTCCAT ATGCCTTATT AGAAGCTAGA AGTTCAGGAG CAGCTTTTAA TTACCTTAAT
BWF451_125  TTCTATTGAA GTATATGAAG AAAAAATGAA TAAATGGGAA CAATTTCCAT ATGCCTTATT AGAAGCTAGA AGTTCAGGAG CAGCTTTTAA TTACCTTAAT
BWF552_125  TTCTATTGAA GTATATGAAG AAAAAATGAA TAAATGGGAA CAATTTCCAT ATGCCTTATT AGAAGCTAGA AGTTCAGGAG CAGCTTTTAA TTACCTTAAT
BWF553_125  TTCTATTGAA GTATATGAAG AAAAAATGAA TAAATGGGAA CAATTTCCAT ATGCCTTATT AGAAGCTAGA AGTTCAGGAG CAGCTTTTAA TTACCTTAAT
BWF554_125  TTCTATTGAA GTATATGAAG AAAAAATGAA TAAATGGGAA CAATTTCCAT ATGCCTTATT AGAAGCTAGA AGTTCAGGAG CAGCTTTTAA TTACCTTAAT
BWF555_125  TTCTATTGAA GTATATGAAG AAAAAATGAA TAAATGGGAA CAATTTCCAT ATGCCTTATT AGAAGCTAGA AGTTCAGGAG CAGCTTTTAA TTACCTTAAT
BWF556_125  TTCTATTGAA GTATATGAAG AAAAAATGAA TAAATGGGAA CAATTTCCAT ATGCCTTATT AGAAGCTAGA AGTTCAGGAG CAGCTTTTAA TTACCTTAAT
BWF557_125  TTCTATTGAA GTATATGAAG AAAAAATGAA TAAATGGGAA CAATTTCCAT ATTCCTTATT AGAAGCTAGA AGTTCAGGAG CAGCTTTTAA TTACCTTAAT
BWF558_125  TTCTATTGAA GTATATGAAG AAAAAATGAA TAAATGGGAA CAATTTCCAT ATGCCTTATT AGAAGCTAGA AGTTCAGGAG CAGCTTTTAA TTACCTTAAT
BWF559_125  TTCTATTGAA GTATATGAAG AAAAAATGAA TAAATGGGAA CAATTTCCAT ATGCCTTATT AGAAGCTAGA AGTTCAGGAG CAGCTTTTAA TTACCTTAAT
BWF560_125  TTCTATTGAA GTATATGAAG AAAAAATGAA TAAATGGGAA CAATTTCCAT ATGCCTTATT AGAAGCTAGA AGTTCAGGAG CAGCTTTTAA TTACCTTAAT
BWF561_125  TTCTATTGAA GTATATGAAG AAAAAATGAA TAAATGGGAA CAATTTCCAT ATGCCTTATT AGAAGCTAGA AGTTCAGGAG CAGCTTTTAA TTACCTTAAT

            1901                                                                                                    2000
       3D7  CAAATATATG TTGTTGGAGG TATTGATAAT GAACATAACA TATTAGATTC CGTTGAACAA TATCAACCAT TTAATAAAAG ATGGCAATTT CTAAATGGTG
   CBE467_  CAAATATATG TTGTTGGAGG TATTGATAAT GAACATAACA TATTAGATTC CGTTGAACAA TATCAACCAT TTAATAAAAG ATGGCAATTT CTAAATGGTG
BWF530_125  CAAATATATG TTGTTGGAGG TATTGATAAT GAACATAACA TATTAGATTC CGTTGAACAA TATCAACCAT TTAATAAAAG ATGGCAATTT CTAAATGGTG
BWF531_125  CAAATATATG TTGTTGGAGG TATTGATAAT GAACATAACA TATTAGATTC CGTTGAACAA TATCAACCAT TTAATAAAAG ATGGCAATTT CTAAATGGTG
BWF532_125  CAAATATATG TTGTTGGAGG TATTGATAAT GAACATAACA TATTAGATTC CGTTGAACAA TATCAACCAT TTAATAAAAG ATGGCAATTT CTAAATGGTG
BWF533_125  CAAATATATG TTGTTGGAGG TATTGATAAT GAACATAACA TATTAGATTC CGTTGAACAA TATCAACCAT TTAATAAAAG ATGGCAATTT CTAAATGGTG
BWF534_125  CAAATATATG TTGTTGGAGG TATTGATAAT GAACATAACA TATTAGATTC CGTTGAACAA TATCAACCAT TTAATAAAAG ATGGCAATTT CTAAATGGTG
BWF536_125  CAAATATATG TTGTTGGAGG TATTGATAAT GAACATAACA TATTAGATTC CGTTGAACAA TATCAACCAT TTAATAAAAG ATGGCAATTT CTAAATGGTG
BWF537_125  CAAATATATG TTGTTGGAGG TATTGATAAT GAACATAACA TATTAGATTC CGTTGAACAA TATCAACCAT TTAATAAAAG ATGGCAATTT CTAAATGGTG
BWF538_125  CAAATATATG TTGTTGGAGG TATTGATAAT GAACATAACA TATTAGATTC CGTTGAACAA TATCAACCAT TTAATAAAAG ATGGCAATTT CTAAATGGTG
BWF539_125  CAAATATATG TTGTTGGAGG TATTGATAAT GAACATAACA TATTAGATTC CGTTGAACAA TATCAACCAT TTAATAAAAG ATGGCAATTT CTAAATGGTG
BWF540_125  CAAATATATG TTGTTGGAGG TATTGATAAT GAACATAACA TATTAGATTC CGTTGAACAA TATCAACCAT TTAATAAAAG ATGGCAATTT CTAAATGGTG
BWF541_125  CAAATATATG TTGTTGGAGG TATTGATAAT GAACATAACA TATTAGATTC CGTTGAACAA TATCAACCAT TTAATAAAAG ATGGCAATTT CTAAATGGTG
BWF542_125  CAAATATATG TTGTTGGAGG TATTGATAAT GAACATAACA TATTAGATTC CGTTGAACAA TATCAACCAT TTAATAAAAG ATGGCAATTT CTAAATGGTG
BWF543_125  CAAATATATG TTGTTGGAGG TATTGATAAT GAACATAACA TATTAGATTC CGTTGAACAA TATCAACCAT TTAATAAAAG ATGGCAATTT CTAAATGGTG
BWF544_125  CAAATATATG TTGTTGGAGG TATTGATAAT GAACATAACA TATTAGATTC CGTTGAACAA TATCAACCAT TTAATAAAAG ATGGCAATTT CTAAATGGTG
BWF545_125  CAAATATATG TTGTTGGAGG TATTGATAAT GAACATAACA TATTAGATTC CGTTGAACAA TATCAACCAT TTAATAAAAG ATGGCAATTT CTAAATGGTG
BWF546_125  CAAATATATG TTGTTGGAGG TATTGATAAT GAACATAACA TATTAGATTC CGTTGAACAA TATCAACCAT TTAATAAAAG ATGGCAATTT CTAAATGGTG
BWF547_125  CAAATATATG TTGTTGGAGG TATTGATAAT GAACATAACA TATTAGATTC CGTTGAACAA TATCAACCAT TTAATAAAAG ATGGCAATTT CTAAATGGTG
BWF548_125  CAAATATATG TTGTTGGAGG TATTGATAAT GAACATAACA TATTAGATTC CGTTGAACAA TATCAACCAT TTAATAAAAG ATGGCAATTT CTAAATGGTG
BWF549_125  CAAATATATG TTGTTGGAGG TATTGATAAT GAACATAACA TATTAGATTC CGTTGAACAA TATCAACCAT TTAATAAAAG ATGGCAATTT CTAAATGGTG
BWF550_125  CAAATATATG TTGTTGGAGG TATTGATAAT GAACATAACA TATTAGATTC CGTTGAACAA TATCAACCAT TTAATAAAAG ATGGCAATTT CTAAATGGTG
BWF551_125  CAAATATATG TTGTTGGAGG TATTGATAAT GAACATAACA TATTAGATTC CGTTGAACAA TATCAACCAT TTAATAAAAG ATGGCGATTT CTAAATGGTG
BWF552_125  CAAATATATG TTGTTGGAGG TATTGATAAT GAACATAACA TATTAGATTC CGTTGAACAA TATCAACCAT TTAATAAAAG ATGGCAATTT CTAAATGGTG
BWF553_125  CAAATATATG TTGTTGGAGG TATTGATAAT GAACATAACA TATTAGATTC CGTTGAACAA TATCAACCAT TTAATAAAAG ATGGCAATTT CTAAATGGTG
BWF554_125  CAAATATATG TTGTTGGAGG TATTGATAAT GAACATAACA TATTAGATTC CGTTGAACAA TATCAACCAT TTAATAAAAG ATGGCAATTT CTAAATGGTG
BWF555_125  CAAATATATG TTGTTGGAGG TATTGATAAT GAACATAACA TATTAGATTC CGTTGAACAA TATCAACCAT TTAATAAAAG ATGGCAATTT CTAAATGGTG
```

```
BWF556_125  CAAATATATG TTGTTGGAGG TATTGATAAT GAACATAACA TATTAGATTC CGTTGAACAA TATCAACCAT TTAATAAAAG ATGGCAATTT CTAAATGGTG
BWF557_125  CAAATATATG TTGTTGGAGG TATTGATAAT GAACATAACA TATTAGATTC CGTTGAACAA TATCAACCAT TTAATAATAG ATGGCAATTT CTAAATGGTG
BWF558_125  CAAATATATG TTGTTGGAGG TATTGATAAT GAACATAACA TATTAGATTC CGTTGAACAA TATCAACCAT TTAATAAAAG ATGGCAATTT CTAAATGGTG
BWF559_125  CAAATATATG TTGTTGGAGG TATTGATAAT GAACATAACA TATTAGATTC CGTTGAACAA TATCAACCAT TTAATAAAAG ATGGCAATTT CTAAATGGTG
BWF560_125  CAAATATATG TTGTTGGAGG TATTGATAAT GAACATAACA TATTAGATTC CGTTGAACAA TATCAACCAT TTAATAAAAG ATGGCAATTT CTAAATGGTG
BWF561_125  CTAATATATG CTGTAG

            1401                                                                                                      1500
       3D7  TCAACAATGC TGGCGTATGT GTACACCTAT GTCTACCAAA AAAGCTTATT TTGGAAGTGC TGTATTGAAT AATTTCTTAT ACGTTTTTGG TGGTAATAAC
CBE469_     TCAACAATGC TGGCGTATGT GTACACCTAT GTCTACCAAA AAAGCTTATT TTGGAAGTGC TGTATTGAAT AATTTCTTAT ACGTTTTTGG TGGTAATAAC
CBE470_     TCAACAATGC TGGCGTATGT GTACACCTAT GTCTACCAAA AAAGCTTATT TTGGAAGTGC TGTATTGAAT AATTTCTTAT ACGTTTTTGG TGGTAATAAC
CBE471_     TCAACAATGC TGGCGTATGT GTACACCTAT GTCTACCAAA AAAGCTTATT TTGGAAGTGC TGTATTGAAT AATTTCTTAT ACGTTTTTGG TGGTAATAAC
CBE472_     TCAACAATGC TGGCGTATGT GTACACCTAT GTCTACCAAA AAAGCTTATT TTGGAAGTGC TGTATTGAAT AATTTCTTAT ACGTTTTTGG TGGTAATAAC
CBE473_     TCAACAATGC TGGCGTATGT GTACACCTAT GTCTACCAAA AAAGCTTATT TTGGAAGTGC TGTATTGAAT AATTTCTTAT ACGTTTTTGG TGGTAATAAC
CBE474_     TCAACAATGC TGGCGTATGT GTACACCTAT GTCTACCAAA AAAGCTTATT TTGGAAGTGC TGTATTGAAT AATTTCTTAT ACGTTTTTGG TGGTAATAAC
CBE475_     TCAACAATGC TGGCGTATGT GTACACCTAT GTCTACCAAA AAAGCTTATT TTGGAAGTGC TGTATTGAAT AATTTCTTAT ACGTTTTTGG TGGTAATAAC
CBE476_     TCAACAATGC TGGCGTATGT GTACACCTAT GTCTACCAAA AAAGCTTATT TTGGAAGTGC TGTATTGAAT AATTTCTTAT ACGTTTTTGG TGGTAATAAC
CBE477_     TCAACAATGC TGGCGTATGT GTACACCTAT GTCTACCAAA AAAGCTTATT TTGGAAGTGC TGTATTGAAT AATTTCTTAT ACGTTTTTGG TGGTAATAAC
CBE478_     TCAACAATGC TGGCGTATGT GTACACCTAT GTCTACCAAA AAAGCTTATT TTGGAAGTGC TGTATTGAAT AATTTCTTAT ACGTTTTTGG TGGTAATAAC
CBE479_     TCAACAATGC TGGCGTATGT GTACACCTAT GTCTACCAAA AAAGCTTATT TTGGAAGTGC TGTATTGAAT AATTTCTTAT ACGTTTTTGG TGGTAATAAC
CBE480_     TCAACAATGC TGGCGTATGT GTACACCTAT GTCTACCAAA AAAGCTTATT TTGGAAGTGC TGTATTGAAT AATTTCTTAT ACGTTTTTGG TGGTAATAAC
CBE481_     TCAACAATGC TGGCGTATGT GTACACCTAT GTCTACCAAA AAAGCTTATT TTGGAAGTGC TGTATTGAAT AATTTCTTAT ACGTTTTTGG TGGTAATAAC
CBE482_     TCAACAATGC TGGCGTATGT GTACACCTAT GTCTACCAAA AAAGCTTATT TTGGAAGTGC TGTATTGAAT AATTTCTTAT ACGTTTTTGG TGGTAATAAC
CBE483_     TCAACAATGC TGGCGTATGT GTACACCTAT GTCTACCAAA AAAGCTTATT TTGGAAGTGC TGTATTGAAT AATTTCTTAT ACGTTTTTGG TGGTAATAAC
CBE484_     TCAACAATGC TGGCGTATGT GTACACCTAT GTCTACCAAA AAAGCTTATT TTGGAAGTGC TGTATTGAAT AATTTCTTAT ACGTTTTTGG TGGTAATAAC
CBE485_     TCAACAATGC TGGCGTATGT GTACACCTAT GTCTACCAAA AAAGCTTATT TTGGAAGTGC TGTATTGAAT AATTTCTTAT ACGTTTTTGG TGGTAATAAC
CBE486_     TCAACAATGC TGGCGTATGT GTACACCTAT GTCTACCAAA AAAGCTTATT TTGGAAGTGC TGTATTGAAT AATTTCTTAT ACGTTTTTGG TGGTAATAAC
CBE487_     TCAACAATGC TGGCGTATGT GTACACCTAT GTCTACCAAA AAAGCTTATT TTGGAAGTGC TGTATTGAAT AATTTCTTAT ACGTTTTTGG TGGTAATAAC
CBE488_     TCAACAATGC TGGCGTATGT GTACACCTAT GTCTACCAAA AAAGCTTATT TTGGAAGTGC TGTATTGAAT AATTTCTTAT ACGTTTTTGG TGGTAATAAC
CBE489_     TCAACAATGC TGGCGTATGT GTACACCTAT GTCTACCAAA AAAGCTTATT TTGGAAGTGC TGTATTGAAT AATTTCTTAT ACGTTTTTGG TGGTAATAAC
CBE490_     TCAACAATGC TGGCGTATGT GTACACCTAT GTCTACCAAA AAAGCTTATT TTGGAAGTGC TGTATTGAAT AATTTCTTAT ACGTTTTTGG TGGTAATAAC
CBE491_     TCAACAATGC TGGCGTATGT GTACACCTAT GTCTACCAAA AAAGCTTATT TTGGAAGTGC TGTATTGAAT AATTTCTTAT ACGTTTTTGG TGGTAATAAC
CBE492_     TCAACAATGC TGGCGTATGT GTACACCTAT GTCTACCAAA AAAGCTTATT TTGGAAGTGC TGTATTGAAT AATTTCTTAT ACGTTTTTGG TGGTAATAAC
CBE493_     TCAACAATGC TGGCGTATGT GTACACCTAT GTCTACCAAA AAAGCTTATT TTGGAAGTGC TGTATTGAAT AATTTCTTAT ACGTTTTTGG TGGTAATAAC
CBE494_     TCAACAATGC TGGCGTATGT GTACACCTAT GTCTACCAAA AAAGCTTATT TTGGAAGTGC TGTATTGAAT AATTTCTTAT ACGTTTTTGG TGGTAATAAC

            1501                                                                                                      1600
       3D7  TATGATTATA AGGCTTTATT TGAAACTGAG GTGTATGATC GTTTAAGAGA TGTATGGTAT GTTTCAAGTA ATTTAAATAT ACCTAGAAGA AATAATTGTG
CBE469_     TATGATTATA AGGCTTTATT TGAAACTGAG GTGTATGATC GTTTAAGAGA TGTATGGTAT GTTTCAAGTA ATTTAAATAT ACCTAGAAGA AATAATTGTG
CBE470_     TATGATTATA AGGCTTTATT TGAAACTGAG GTGTATGATC GTTTAAGAGA TGTATGGTAT GTTTCAAGTA ATTTAAATAT ACCTAGAAGA AATAATTGTG
```

```
CBE471_     TATGATTATA AGGCTTTATT TGAAACTGAG GTGTATGATC GTTTAAGAGA TGTATGGTAT GTTTCAAGTA ATTTAAATAT ACCTAGAAGA AATAATTGTG
CBE472_     TATGATTATA AGGCTTTATT TGAAACTGAG GTGTATGATC GTTTAAGAGA TGTATGGTAT GTTTCAAGTA ATTTAAATAT ACCTAGAAGA AATAATTGTG
CBE473_     TATGATTATA AGGCTTTATT TGAAACTGAG GTGTATGATC GTTTAAGAGA TGTATGGTAT GTTTCAAGTA ATTTAAATAT ACCTAGAAGA AATAATTGTG
CBE474_     TATGATTATA AGGCTTTATT TGAAACTGAG GTGTATGATC GTTTAAGAGA TGTATGGTAT GTTTCAAGTA ATTTAAATAT ACCTAGAAGA AATAATTGTG
CBE475_     TATGATTATA AGGCTTTATT TGAAACTGAG GTGTATGATC GTTTAAGAGA TGTATGGTAT GTTTCAAGTA ATTTAAATAT ACCTAGAAGA AATAATTGTG
CBE476_     TATGATTATA AGGCTTTATT TGAAACTGAG GTGTATGATC GTTTAAGAGA TGTATGGTAT GTTTCAAGTA ATTTAAATAT ACCTAGAAGA AATAATTGTG
CBE477_     TATGATTATA AGGCTTTATT TGAAACTGAG GTGTATGATC GTTTAAGAGA TGTATGGTAT GTTTCAAGTA ATTTAAATAT ACCTAGAAGA AATAATTGTG
CBE478_     TATGATTATA AGGCTTTATT TGAAACTGAG GTGTATGATC GTTTAAGAGA TGTATGGTAT GTTTCAAGTA ATTTAAATAT ACCTAGAAGA AATAATTGTG
CBE479_     TATGATTATA AGGCTTTATT TGAAACTGAG GTGTATGATC GTTTAAGAGA TGTATGGTAT GTTTCAAGTA ATTTAAATAT ACCTAGAAGA AATAATTGTG
CBE480_     TATGATTATA AGGCTTTATT TGAAACTGAG GTGTATGATC GTTTAAGAGA TGTATGGTAT GTTTCAAGTA ATTTAAATAT ACCTAGAAGA AATAATTGTG
CBE481_     TATGATTATA AGGCTTTATT TGAAACTGAG GTGTATGATC GTTTAAGAGA TGTATGGTAT GTTTCAAGTA ATTTAAATAT ACCTAGAAGA AATAATTGTG
CBE482_     TATGATTATA AGGCTTTATT TGAAACTGAG GTGTATGATC GTTTAAGAGA TGTATGGTAT GTTTCAAGTA ATTTAAATAT ACCTAGAAGA AATAATTGTG
CBE483_     TATGATTATA AGGCTTTATT TGAAACTGAG GTGTATGATC GTTTAAGAGA TGTATGGTAT GTTTCAAGTA ATTTAAATAT ACCTAGAAGA AATAATTGTG
CBE484_     TATGATTATA AGGCTTTATT TGAAACTGAG GTGTATGATC GTTTAAGAGA TGTATGGTAT GTTTCAAGTA ATTTAAATAT ACCTAGAAGA AATAATTGTG
CBE485_     TATGATTATA AGGCTTTATT TGAAACTGAG GTGTATGATC GTTTAAGAGA TGTATGGTAT GTTTCAAGTA ATTTAAATAT ACCTAGAAGA AATAATTGTG
CBE486_     TATGATTATA AGGCTTTATT TGAAACTGAG GTGTATGATC GTTTAAGAGA TGTATGGTAT GTTTCAAGTA ATTTAAATAT ACCTAGAAGA AATAATTGTG
CBE487_     TATGATTATA AGGCTTTATT TGAAACTGAG GTGTATGATC GTTTAAGAGA TGTATGGTAT GTTTCAAGTA ATTTAAATAT ACCTAGAAGA AATAATTGTG
CBE488_     TATGATTATA AGGCTTTATT TGAAACTGAG GTGTATGATC GTTTAAGAGA TGTATGGTAT GTTTCAAGTA ATTTAAATAT ACCTAGAAGA AATAATTGTG
CBE489_     TATGATTATA AGGCTTTATT TGAAACTGAG GTGTATGATC GTTTAAGAGA TGTATGGTAT GTTTCAAGTA ATTTAAATAT ACCTAGAAGA AATAATTGTG
CBE490_     TATGATTATA AGGCTTTATT TGAAACTGAG GTGTATGATC GTTTAAGAGA TGTATGGTAT GTTTCAAGTA ATTTAAATAT ACCTAGAAGA AATAATTGTG
CBE491_     TATGATTATA AGGCTTTATT TGAAACTGAG GTGTATGATC GTTTAAGAGA TGTATGGTAT GTTTCAAGTA ATTTAAATAT ACCTAGAAGA AATAATTGTG
CBE492_     TATGATTATA AGGCTTTATT TGAAACTGAG GTGTATGATC GTTTAAGAGA TGTATGGTAT GTTTCAAGTA ATTTAAATAT ACCTAGAAGA AATAATTGTG
CBE493_     TATGATTATA AGGCTTTATT TGAAACTGAG GTGTATGATC GTTTAAGAGA TGTATGGTAT GTTTCAAGTA ATTTAAATAT ACCTAGAAGA AATAATTGTG
CBE494_     TATGATTATA AGGCTTTATT TGAAACTGAG GTGTATGATC GTTTAAGAGA TGTATGGTAT GTTTCAAGTA ATTTAAATAT ACCTAGAAGA AATAATTGTG

            1601                                                                                                   1700
        3D7 GTGTTACGTC AAATGGTAGA ATTTATTGTA TTGGGGGATA TGATGGCTCT TCTATTATAC CCGAATGTAG AAGCATATGA TCATCGTATG AAAGCATGGG
CBE469_     GTGTTACGTC AAATGGTAGA ATTTATTGTA TTGGGGGATA TGATGGCTCT TCTATTATAC CCGAATGTAG AAGCATATGA TCATCGTATG AAAGCATGGG
CBE470_     GTGTTACGTC AAATGGTAGA ATTTATTGTA TTGGGGGATA TGATGGCTCT TCTATTATAC CCGAATGTAG AAGCATATGA TCATCGTATG AAAGCATGGG
CBE471_     GTGTTACGTC AAATGGTAGA ATTTATTGTA TTGGGGGATA TGATGGCTCT TCTATTATAC CCGAATGTAG AAGCATATGA TCATCGTATG AAAGCATGGG
CBE472_     GTGTTACGTC AAATGGTAGA ATTTATTGTA TTGGGGGATA TGATGGCTCT TCTATTATAC CCGAATGTAG AAGCATATGA TCATCGTATG AAAGCATGGG
CBE473_     GTGTTACGTC AAATGGTAGA ATTTATTGTA TTGGGGGATA TGATGGCTCT TCTATTATAC CCGAATGTAG AAGCATATGA TCATCGTATG AAAGCATGGG
CBE474_     GTGTTACGTC AAATGGTAGA ATTTATTGTA TTGGGGGATA TGATGGCTCT TCTATTATAC CCGAATGTAG AAGCATATGA TCATCGTATG AAAGCATGGG
CBE475_     GTGTTACGTC AAATGGTAGA ATTTATTGTA TTGGGGGATA TGATGGCTCT TCTATTATAC CCGAATGTAG AAGCATATGA TCATCGTATG AAAGCATGGG
CBE476_     GTGTTACGTC AAATGGTAGA ATTTATTGTA TTGGGGGATA TGATGGCTCT TCTATTATAC CCGAATGTAG AAGCATATGA TCATCGTATG AAAGCATGGG
CBE477_     GTGTTACGTC AAATGGTAGA ATTTATTGTA TTGGGGGATA TGATGGCTCT TCTATTATAC CCGAATGTAG AAGCATATGA TCATCGTATG AAAGCATGGG
CBE478_     GTGTTACGTC AAATGGTAGA ATTTATTGTA TTGGGGGATA TGATGGCTCT TCTATTATAC CCGAATGTAG AAGCATATGA TCATCGTATG AAAGCATGGG
CBE479_     GTGTTACGTC AAATGGTAGA ATTTATTGTA TTGGGGGATA TGATGGCTCT TCTATTATAC CCGAATGTAG AAGCATATGA TCATCGTATG AAAGCATGGG
CBE480_     GTGTTACGTC AAATGGTAGA ATTTATTGTA TTGGGGGATA TGATGGCTCT TCTATTATAC CCGAATGTAG AAGCATATGA TCATCGTATG AAAGCATGGG
CBE481_     GTGTTACGTC AAATGGTAGA ATTTATTGTA TTGGGGGATA TGATGGCTCT TCTATTATAC CCGAATGTAG AAGCATATGA TCATCGTATG AAAGCATGGG
CBE482_     GTGTTACGTC AAATGGTAGA ATTTATTGTA TTGGGGGATA TGATGGCTCT TCTATTATAC CCGAATGTAG AAGCATATGA TCATCGTATG AAAGCATGGG
```

```
CBE483_     GTGTTACGTC AAATGGTAGA ATTTATTGTA TTGGGGGATA TGATGGCTCT TCTATTATAC CCGAATGTAG AAGCATATGA TCATCGTATG AAAGCATGGG
CBE484_     GTGTTACGTC AAATGGTAGA ATTTATTGTA TTGGGGGATA TGATGGCTCT TCTATTATAC CCGAATGTAG AAGCATATGA TCATCGTATG AAAGCATGGG
CBE485_     GTGTTACGTC AAATGGTAGA ATTTATTGTA TTGGGGGATA TGATGGCTCT TCTATTATAC CCGAATGTAG AAGCATATGA TCATCGTATG AAAGCATGGG
CBE486_     GTGTTACGTC AAATGGTAGA ATTTATTGTA TTGGGGGATA TGATGGCTCT TCTATTATAC CCGAATGTAG AAGCATATGA TCATCGTATG AAAGCATGGG
CBE487_     GTGTTACGTC AAATGGTAGA ATTTATTGTA TTGGGGGATA TGATGGCTCT TCTATTATAC CCGAATGTAG AAGCATATGA TCATCGTATG AAAGCATGGG
CBE488_     GTGTTACGTC AAATGGTAGA ATTTATTGTA TTGGGGGATA TGATGGCTCT TCTATTATAC CCGAATGTAG AAGCATATGA TCATCGTATG AAAGCATGGG
CBE489_     GTGTTACGTC AAATGGTAGA ATTTATTGTA TTGGGGGATA TGATGGCTCT TCTATTATAC CCGAATGTAG AAGCATATGA TCATCGTATG AAAGCATGGG
CBE490_     GTGTTACGTC AAATGGTAGA ATTTATTGTA TTGGGGGATA TGATGGCTCT TCTATTATAC CCGAATGTAG AAGCATATGA TCATCGTATG AAAGCATGGG
CBE491_     GTGTTACGTC AAATGGTAGA ATTTATTGTA TTGGGGGATA TGATGGCTCT TCTATTATAC CCGAATGTAG AAGCATATGA TCATCGTATG AAAGCATGGG
CBE492_     GTGTTACGTC AAATGGTAGA ATTTATTGTA TTGGGGGATA TGATGGCTCT TCTATTATAC CCGAATGTAG AAGCATATGA TCATCGTATG AAAGCATGGG
CBE493_     GTGTTACGTC AAATGGTAGA ATTTATTGTA TTGGGGGATA TGATGGCTCT TTTATTATAC CCGAATGTAG AAGCATATGA TCATCGTATG AAAGCATGGG
CBE494_     GTGTTACGTC AAATGGTAGA ATTTATTGTA TTGGGGGATA TGATGGCTCT TCTATTATAC CCGAATGTAG AAGCATATGA TCATCGTATG AAAGCATGGG

            1701                                                                                                    1800
       3D7  TAGAGGTGGC ACCTTTGAAT ACCCCTAGAT CATCAGCTAT GTGTGTTGCT TTTGATAATA AAATTTATGT CATTGGTGGA ACTAATGGTG AGAGATTAAA
CBE469_     TAGAGGTGGC ACCTTTGAAT ACCCCTAGAT CATCAGCTAT GTGTGTTGCT TTTGATAATA AAATTTATGT CATTGGTGGA ACTAATGGTG AGAGATTAAA
CBE470_     TAGAGGTGGC ACCTTTGAAT ACCCCTAGAT CATCAGCTAT GTGTGTTGCT TTTGATAATA AAATTTATGT CATTGGTGGA ACTAATGGTG AGAGATTAAA
CBE471_     TAGAGGTGGC ACCTTTGAAT ACCCCTAGAT CATCAGCTAT GTGTGTTGCT TTTGATAATA AAATTTATGT CATTGGTGGA ACTAATGGTG AGAGATTAAA
CBE472_     TAGAGGTGGC ACCTTTGAAT ACCCCTAGAT CATCAGCTAT GTGTGTTGCT TTTGATAATA AAATTTATGT CATTGGTGGA ACTAATGGTG AGAGATTAAA
CBE473_     TAGAGGTGGC ACCTTTGAAT ACCCCTAGAT CATCAGCTAT GTGTGTTGCT TTTGATAATA AAATTTATGT CATTGGTGGA ACTAATGGTG AGAGATTAAA
CBE474_     TAGAGGTGGC ACCTTTGAAT ACCCCTAGAT CATCAGCTAT GTGTGTTGCT TTTGATAATA AAATTTATGT CATTGGTGGA ACTAATGGTG AGAGATTAAA
CBE475_     TAGAGGTGGC ACCTTTGAAT ACCCCTAGAT CATCAGCTAT GTGTGTTGCT TTTGATAATA AAATTTATGT CATTGGTGGA ACTAATGGTG AGAGATTAAA
CBE476_     TAGAGGTGGC ACCTTTGAAT ACCCCTAGAT CATCAGCTAT GTGTGTTGCT TTTGATAATA AAATTTATGT CATTGGTGGA ACTAATGGTG AGAGATTAAA
CBE477_     TAGAGGTGGC ACCTTTGAAT ACCCCTAGAT CATCAGCTAT GTGTGTTGCT TTTGATAATA AAATTTATGT CATTGGTGGA ACTAATGGTG AGAGATTAAA
CBE478_     TAGAGGTGGC ACCTTTGAAT ACCCCTAGAT CATCAGCTAT GTGTGTTGCT TTTGATAATA AAATTTATGT CATTGGTGGA ACTAATGGTG AGAGATTAAA
CBE479_     TAGAGGTGGC ACCTTTGAAT ACCCCTAGAT CATCAGCTAT GTGTGTTGCT TTTGATAATA AAATTTATGT CATTGGTGGA ACTAATGGTG AGAGATTAAA
CBE480_     TAGAGGTGGC ACCTTTGAAT ACCCCTAGAT CATCAGCTAT GTGTGTTGCT TTTGATAATA AAATTTATGT CATTGGTGGA ACTAATGGTG AGAGATTAAA
CBE481_     TAGAGGTGGC ACCTTTGAAT ACCCCTAGAT CATCAGCTAT GTGTGTTGCT TTTGATAATA AAATTTATGT CATTGGTGGA ACTAATGGTG AGAGATTAAA
CBE482_     TAGAGGTGGC ACCTTTGAAT ACCCCTAGAT CATCAGCTAT GTGTGTTGCT TTTGATAATA AAATTTATGT CATTGGTGGA ACTAATGGTG AGAGATTAAA
CBE483_     TAGAGGTGGC ACCTTTGAAT ACCCCTAGAT CATCAGCTAT GTGTGTTGCT TTTGATAATA AAATTTATGT CATTGGTGGA ACTAATGGTG AGAGATTAAA
CBE484_     TAGAGGTGGC ACCTTTGAAT ACCCCTAGAT CATCAGCTAT GTGTGTTGCT TTTGATAATA AAATTTATGT CATTGGTGGA ACTAATGGTG AGAGATTAAA
CBE485_     TAGAGGTGGC ACCTTTGAAT ACCCCTAGAT CATCAGCTAT GTGTGTTGCT TTTGATAATA AAATTTATGT CATTGGTGGA ACTAATGGTG AGAGATTAAA
CBE486_     TAGAGGTGGC ACCTTTGAAT ACCCCTAGAT CATCAGCTAT GTGTGTTGCT TTTGATAATA AAATTTATGT CATTGGTGGA ACTAATGGTG AGAGATTAAA
CBE487_     TAGAGGTGGC ACCTTTGAAT ACCCCTAGAT CATCAGCTAT GTGTGTTGCT TTTGATAATA AAATTTATGT CATTGGTGGA ACTAATGGTG AGAGATTAAA
CBE488_     TAGAGGTGGC ACCTTTGAAT ACCCCTAGAT CATCAGCTAT GTGTGTTGCT TTTGATAATA AAATTTATGT CATTGGTGGA ACTAATGGTG AGAGATTAAA
CBE489_     TAGAGGTGGC ACCTTTGAAT ACCCCTAGAT CATCAGCTAT GTGTGTTGCT TTTGATAATA AAATTTATGT CATTGGTGGA ACTAATGGTG AGAGATTAAA
CBE490_     TAGAGGTGGC ACCTTTGAAT ACCCCTAGAT CATCAGCTAT GTGTGTTGCT TTTGATAATA AAATTTATGT CATTGGTGGA ACTAATGGTG AGAGATTAAA
CBE491_     TAGAGGTGGC ACCTTTGAAT ACCCCTAGAT CATCAGCTAT GTGTGTTGCT TTTGATAATA AAATTTATGT CATTGGTGGA ACTAATGGTG AGAGATTAAA
CBE492_     TAGAGGTGGC ACCTTTGAAT ACCCCTAGAT CATCAGCTAT GTGTGTTGCT TTTGATAATA AAATTTATGT CATTGGTGGA ACTAATGGTG AGAGATTAAA
CBE493_     TAGAGGTGGC ACCTTTGAAT ACCCCTAGAT CATCAGCTAT GTGTGTTGCT TTTGATAATA AAATTTATGT CATTGGTGGA ACTAATGGTG AGAGATTAAA
CBE494_     TAGAGGTGGC ACCTTTGAAT ACCCCTAGAT CATCAGCTAT GTGTGTTGCT TTTGATAATA AAATTTATGT CATTGGTGGA ACTAATGGTG AGAGATTAAA
```

```
           1801                                                                                                       1900
       3D7 TTCTATTGAA GTATATGAAG AAAAAATGAA TAAATGGGAA CAATTTCCAT ATGCCTTATT AGAAGCTAGA AGTTCAGGAG CAGCTTTTAA TTACCTTAAT
CBE469_    TTCTATTGAA GTATATGAAG AAAAAATGAA TAAATGGGAA CAATTTCCAT ATGCCTTATT AGAAGCTAGA AGTTCAGGAG CAGCTTTTAA TTACCTTAAT
CBE470_    TTCTATTGAA GTATATGAAG AAAAAATGAA TAAATGGGAA CAATTTCCAT ATGCCTTATT AGAAGCTAGA AGTTCAGGAG CAGCTTTTAA TTACCTTAAT
CBE471_    TTCTATTGAA GTATATGAAG AAAAAATGAA TAAATGGGAA CAATTTCCAT ATGCCTTATT AGAAGCTAGA AGTTCAGGAG CAGCTTTTAA TTACCTTAAT
CBE472_    TTCTATTGAA GTATATGAAG AAAAAATGAA TAAATGGGAA CAATTTCCAT ATGCCTTATT AGAAGCTAGA AGTTCAGGAG CAGCTTTTAA TTACCTTAAT
CBE473_    TTCTATTGAA GTATATGAAG AAAAAATGAA TAAATGGGAA CAATTTCCAT ATGCCTTATT AGAAGCTAGA AGTTCAGGAG CAGCTTTTAA TTACCTTAAT
CBE474_    TTCTATTGAA GTATATGAAG AAAAAATGAA TAAATGGGAA CAATTTCCAT ATGCCTTATT AGAAGCTAGA AGTTCAGGAG CAGCTTTTAA TTACCTTAAT
CBE475_    TTCTATTGAA GTATATGAAG AAAAAATGAA TAAATGGGAA CAATTTCCAT ATGCCTTATT AGAAGCTAGA AGTTCAGGAG CAGCTTTTAA TTACCTTAAT
CBE476_    TTCTATTGAA GTATATGAAG AAAAAATGAA TAAATGGGAA CAATTTCCAT ATGCCTTATT AGAAGCTAGA AGTTCAGGAG CAGCTTTTAA TTACCTTAAT
CBE477_    TTCTATTGAA GTATATGAAG AAAAAATGAA TAAATGGGAA CAATTTCCAT ATGCCTTATT AGAAGCTAGA AGTTCAGGAG CAGCTTTTAA TTACCTTAAT
CBE478_    TTCTATTGAA GTATATGAAG AAAAAATGAA TAAATGGGAA CAATTTCCAT ATGCCTTATT AGAAGCTAGA AGTTCAGGAG CAGCTTTTAA TTACCTTAAT
CBE479_    TTCTATTGAA GTATATGAAG AAAAAATGAA TAAATGGGAA CAATTTCCAT ATGCCTTATT AGAAGCTAGA AGTTCAGGAG CAGCTTTTAA TTACCTTAAT
CBE480_    TTCTATTGAA GTATATGAAG AAAAAATGAA TAAATGGGAA CAATTTCCAT ATGCCTTATT AGAAGCTAGA AGTTCAGGAG CAGCTTTTAA TTACCTTAAT
CBE481_    TTCTATTGAA GTATATGAAG AAAAAATGAA TAAATGGGAA CAATTTCCAT ATGCCTTATT AGAAGCTAGA AGTTCAGGAG CAGCTTTTAA TTACCTTAAT
CBE482_    TTCTATTGAA GTATATGAAG AAAAAATGAA TAAATGGGAA CAATTTCCAT ATGCCTTATT AGAAGCTAGA AGTTCAGGAG CAGCTTTTAA TTACCTTAAT
CBE483_    TTCTATTGAA GTATATGAAG AAAAAATGAA TAAATGGGAA CAATTTCCAT ATGCCTTATT AGAAGCTAGA AGTTCAGGAG CAGCTTTTAA TTACCTTAAT
CBE484_    TTCTATTGAA GTATATGAAG AAAAAATGAA TAAATGGGAA CAATTTCCAT ATGCCTTATT AGAAGCTAGA AGTTCAGGAG CAGCTTTTAA TTACCTTAAT
CBE485_    TTCTATTGAA GTATATGAAG AAAAAATGAA TAAATGGGAA CAATTTCCAT ATGCCTTATT AGAAGCTAGA AGTTCAGGAG CAGCTTTTAA TTACCTTAAT
CBE486_    TTCTATTGAA GTATATGAAG AAAAAATGAA TAAATGGGAA CAATTTCCAT ATGCCTTATT AGAAGCTAGA AGTTCAGGAG CAGCTTTTAA TTACCTTAAT
CBE487_    TTCTATTGAA GTATATGAAG AAAAAATGAA TAAATGGGAA CAATTTCCAT ATGCCTTATT AGAAGCTAGA AGTTCAGGAG CAGCTTTTAA TTACCTTAAT
CBE488_    TTCTATTGAA GTATATGAAG AAAAAATGAA TAAATGGGAA CAATTTCCAT ATGCCTTATT AGAAGCTAGA AGTTCAGGAG CAGCTTTTAA TTACCTTAAT
CBE489_    TTCTATTGAA GTATATGAAG AAAAAATGAA TAAATGGGAA CAATTTCCAT ATGCCTTATT AGAAGCTAGA AGTTCAGGAG CAGCTTTTAA TTACCTTAAT
CBE490_    TTCTATTGAA GTATATGAAG AAAAAATGAA TAAATGGGAA CAATTTCCAT ATGCCTTATT AGAAGCTAGA AGTTCAGGAG CAGCTTTTAA TTACCTTAAT
CBE491_    TTCTATTGAA GTATATGAAG AAAAAATGAA TAAATGGGAA CAATTTCCAT ATGCCTTATT AGAAGCTAGA AGTTCAGGAG CAGCTTTTAA TTACCTTAAT
CBE492_    TTCTATTGAA GTATATGAAG AAAAAATGAA TAAATGGGAA CAATTTCCAT ATGCCTTATT AGAAGCTAGA AGTTCAGGAG CAGCTTTTAA TTACCTTAAT
CBE493_    TTCTATTGAA GTATATGAAG AAAAAATGAA TAAATGGGAA CAATTTCCAT ATGCCTTATT AGAAGCTAGA AGTTCAGGAG CAGCTTTTAA TTACCTTAAT
CBE494_    TTCTATTGAA GTATATGAAG AAAAAATGAA TAAATGGGAA CAATTTCCAT ATGCCTTATT AGAAGCTAGA AGTTCAGGAG CAGCTTTTAA TTACCTTAAT

           1901                                                                                                       2000
       3D7 CAAATATATG TTGTTGGAGG TATTGATAAT GAACATAACA TATTAGATTC CGTTGAACAA TATCAACCAT TTAATAAAAG ATGGCAATTT CTAAATGGTG
CBE469_    CAAATATATG TTGTTGGAGG TATTGATAAT GAACATAACA TATTAGATTC CGTTGAACAA TATCAACCAT TTAATAAAAG ATGGCAATTT CTAAATGGTG
CBE470_    CAAATATATG TTGTTGGAGG TATTGATAAT GAACATAACA TATTAGATTC CGTTGAACAA TATCAACCAT TTAATAAAAG ATGGCAATTT CTAAATGGTG
CBE471_    CAAATATATG TTGTTGGAGG TATTGATAAT GAACATAACA TATTAGATTC CGTTGAACAA TATCAACCAT TTAATAAAAG ATGGCAATTT CTAAATGGTG
CBE472_    CAAATATATG TTGTTGGAGG TATTGATAAT GAACATAACA TATTAGATTC CGTTGAACAA TATCAACCAT TTAATAAAAG ATGGCAATTT CTAAATGGTG
CBE473_    CAAATATATG TTGTTGGAGG TATTGATAAT GAACATAACA TATTAGATTC CGTTGAACAA TATCAACCAT TTAATAAAAG ATGGCAATTT CTAAATGGTG
CBE474_    CAAATATATG TTGTTGGAGG TATTGATAAT GAACATAACA TATTAGATTC CGTTGAACAA TATCAACCAT TTAATAAAAG ATGGCAATTT CTAAATGGTG
CBE475_    CAAATATATG TTGTTGGAGG TATTGATAAT GAACATAACA TATTAGATTC CGTTGAACAA TATCAACCAT TTAATAAAAG ATGGCAATTT CTAAATGGTG
CBE476_    CAAATATATG TTGTTGGAGG TATTGATAAT GAACATAACA TATTAGATTC CGTTGAACAA TATCAACCAT TTAATAAAAG ATGGCAATTT CTAAATGGTG
CBE477_    CAAATATATG TTGTTGGAGG TATTGATAAT GAACATAACA TATTAGATTC CGTTGAACAA TATCAACCAT TTAATAAAAG ATGGCAATTT CTAAATGGTG
```

```
CBE478_     CAAATATATG TTGTTGGAGG TATTGATAAT GAACATAACA TATTAGATTC CGTTGAACAA TATCAACCAT TTAATAAAAG ATGGCAATTT CTAAATGGTG
CBE479_     CAAATATATG TTGTTGGAGG TATTGATAAT GAACATAACA TATTAGATTC CGTTGAACAA TATCAACCAT TTAATAAAAG ATGGCAATTT CTAAATGGTG
CBE480_     CAAATATATG TTGTTGGAGG TATTGATAAT GAACATAACA TATTAGATTC CGTTGAACAA TATCAACCAT TTAATAAAAG ATGGCAATTT CTAAATGGTG
CBE481_     CAAATATATG TTGTTGGAGG TATTGATAAT GAACATAACA TATTAGATTC CGTTGAACAA TATCAACCAT TTAATAAAAG ATGGCAATTT CTAAATGGTG
CBE482_     CAAATATATG TTGTTGGAGG TATTGATAAT GAACATAACA TATTAGATTC CGTTGAACAA TATCAACCAT TTAATAAAAG ATGGCAATTT CTAAATGGTG
CBE483_     CAAATATATG TTGTTGGAGG TATTGATAAT GAACATAACA TATTAGATTC CGTTGAACAA TATCAACCAT TTAATAAAAG ATGGCAATTT CTAAATGGTG
CBE484_     CAAATATATG TTGTTGGAGG TATTGATAAT GAACATAACA TATTAGATTC CGTTGAACAA TATCAACCAT TTAATAAAAG ATGGCAATTT CTAAATGGTG
CBE485_     CAAATATATG TTGTTGGAGG TATTGATAAT GAACATAACA TATTAGATTC CGTTGAACAA TATCAACCAT TTAATAAAAG ATGGCAATTT CTAAATGGTG
CBE486_     CAAATATATG TTGTTGGAGG TATTGATAAT GAACATAACA TATTAGATTC CGTTGAACAA TATCAACCAT TTAATAAAAG ATGGCAATTT CTAAATGGTG
CBE487_     CAAATATATG TTGTTGGAGG TATTGATAAT GAACATAACA TATTAGATTC CGTTGAACAA TATCAACCAT TTAATAAAAG ATGGCAATTT CTAAATGGTG
CBE488_     CAAATATATG TTGTTGGAGG TATTGATAAT GAACATAACA TATTAGATTC CGTTGAACAA TATCAACCAT TTAATAAAAG ATGGCAATTT CTAAATGGTG
CBE489_     CAAATATATG TTGTTGGAGG TATTGATAAT GAACATAACA TATTAGATTC CGTTGAACAA TATCAACCAT TTAATAAAAG ATGGCAATTT CTAAATGGTG
CBE490_     CAAATATATG TTGTTGGAGG TATTGATAAT GAACATAACA TATTAGATTC CGTTGAACAA TATCAACCAT TTAATAAAAG ATGGCGATTT CTAAATGGTG
CBE491_     CAAATATATG TTGTTGGAGG TATTGATAAT GAACATAACA TATTAGATTC CGTTGAACAA TATCAACCAT TTAATAAAAG ATGGCAATTT CTAAATGGTG
CBE492_     CAAATATATG TTGTTGGAGG TATTGATAAT GAACATAACA TATTAGATTC CGTTGAACAA TATCAACCAT TTAATAAAAG ATGGCAATTT CTAAATGGTG
CBE493_     CAAATATATG TTGTTGGAGG TATTGATAAT GAACATAACA TATTAGATTC CGTTGAACAA TATCAACCAT TTAATAAAAG ATGGCAATTT CTAAATGGTG
CBE494_     CAAATATATG TTGTTGGAGG TATTGATAAT GAACATAACA TATTAGATTC CGTTGAACAA TATCAACCAT TTAATAAAAG ATGGCAATTT CTAAATGGTG
```

Senegal Sequences K13
2017

```
            1401                                                                                                      1500
       3D7  ACAATGCTGG CGTATGTGTA CACCTATGTC TACCAAAAAA GCTTATTTTG GAAGTGCTGT ATTGAATAAT TTCTTATACG TTTTTGGTGG TAATAACTAT
BWF473_125  ACAATGCTGG CGTATGTGTA CACCTATGTC TACCAAAAAA GCTTATTTTG GAAGTGCTGT ATTGAATAAT TTCTTATACG TTTTTGGTGG TAATAACTAT
52AI99_193  ACAATGCTGG CGTATGTGTA CACCTATGTC TACCAAAAAA GCTTATTTTG GAAGTGCTGT ATTGAATAAT TTCTTATACG TTTTTGGTGG TAATAACTAT
BWF466_125  ACAATGCTGG CGTATGTGTA CACCTATGTC TACCAAAAAA GCTTATTTTG GAAGTGCTGT ATTGAATAAT TTCTTATACG TTTTTGGTGG TAATAACTAT
BWF485_125  ACAATGCTGG CGTATGTGTA CACCTATGTC TACCAAAAAA GCTTATTTTG GAAGTGCTGT ATTGAATAAT TTCTTATACG TTTTTGGTGG TAATAACTAT
BWF472_125  ACAATGCTGG CGTATGTGTA CACCTATGTC TACCAAAAAA GCTTATTTTG GAAGTGCTGT ATTGAATAAT TTCTTATACG TTTTTGGTGG TAATAACTAT
BWF479_125  ACAATGCTGG CGTATGTGTA CACCTATGTC TACCAAAAAA GCTTATTTTG GAAGTGCTGT ATTGAATAAT TTCTTATACG TTTTTGGTGG TAATAACTAT
BWF468_125  ACAATGCTGG CGTATGTGTA CACCTATGTC TACCAAAAAA GCTTATTTTG GAAGTGCTGT ATTGAATAAT TTCTTATACG TTTTTGGTGG TAATAACTAT
52AJ00_193  ACAATGCTGG CGTATGTGTA CACCTATGTC TACCAAAAAA GCTTATTTTG GAAGTGCTGT ATTGAATAAT TTCTTATACG TTTTTGGTGG TAATAACTAT
BWF465_125  ACAATGCTGG CGTATGTGTA CACCTATGTC TACCAAAAAA GCTTATTTTG GAAGTGCTGT ATTGAATAAT TTCTTATACG TTTTTGGTGG TAATAACTAT
BWF477_125  ACAATGCTGG CGTATGTGTA CACCTATGTC TACCAAAAAA GCTTATTTTG GAAGTGCTGT ATTGAATAAT TTCTTATACG TTTTTGGTGG TAATAACTAT
BWF471_125  ACAATGCTGG CGTATGTGTA CACCTATGTC TACCAAAAAA GCTTATTTTG GAAGTGCTGT ATTGAATAAT TTCTTATACG TTTTTGGTGG TAATAACTAT
BWF489_125  ACAATGCTGG CGTATGTGTA CACCTATGTC TACCAAAAAA GCTTATTTTG GAAGTGCTGT ATTGAATAAT TTCTTATACG TTTTTGGTGG TAATAACTAT

            1501                                                                                                      1600
       3D7  GATTATAAGG CTTTATTTGA AACTGAGGTG TATGATCGTT TAAGAGATGT ATGGTATGTT TCAAGTAATT TAAATATACC TAGAAGAAAT AATTGTGGTG
BWF473_125  GATTATAAGG CTTTATTTGA AACTGAGGTG TATGATCGTT TAAGAGATGT ATGGTATGTT TCAAGTAATT TAAATATACC TAGAAGAAAT AATTGTGGTG
52AI99_193  GATTATAAGG CTTTATTTGA AACTGAGGTG TATGATCGTT TAAGAGATGT ATGGTATGTT TCAAGTAATT TAAATATACC TAGAAGAAAT AATTGTGGTG
BWF466_125  GATTATAAGG CTTTATTTGA AACTGAGGTG TATGATCGTT TAAGAGATGT ATGGTATGTT TCAAGTAATT TAAATATACC TAGAAGAAAT AATTGTGGTG
```

```
BWF485_125  GATTATAAGG CTTTATTTGA AACTGAGGTG TATGATCGTT TAAGAGATGT ATGGTATGTT TCAAGTAATT TAAATATACC TAGAAGAAAT AATTGTGGTG
BWF472_125  GATTATAAGG CTTTATTTGA AACTGAGGTG TATGATCGTT TAAGAGATGT ATGGTATGTT TCAAGTAATT TAAATATACC TAGAAGAAAT AATTGTGGTG
BWF479_125  GATTATAAGG CTTTATTTGA AACTGAGGTG TATGATCGTT TAAGAGATGT ATGGTATGTT TCAAGTAATT TAAATATACC TAGAAGAAAT AATTGTGGTG
BWF468_125  GATTATAAGG CTTTATTTGA AACTGAGGTG TATGATCGTT TAAGAGATGT ATGGTATGTT TCAAGTAATT TAAATATACC TAGAAGAAAT AATTGTGGTG
52AJ00_193  GATTATAAGG CTTTATTTGA AACTGAGGTG TATGATCGTT TAAGAGATGT ATGGTATGTT TCAAGTAATT TAAATATACC TAGAAGAAAT AATTGTGGTG
BWF465_125  GATTATAAGG CTTTATTTGA AACTGAGGTG TATGATCGTT TAAGAGATGT ATGGTATGTT TCAAGTAATT TAAATATACC TAGAAGAAAT AATTGTGGTG
BWF477_125  GATTATAAGG CTTTATTTGA AACTGAGGTG TATGATCGTT TAAGAGATGT ATGGTATGTT TCAAGTAATT TAAATATACC TAGAAGAAAT AATTGTGGTG
BWF471_125  GATTATAAGG CTTTATTTGA AACTGAGGTG TATGATCGTT TAAGAGATGT ATGGTATGTT TCAAGTAATT TAAATATACC TAGAAGAAAT AATTGTGGTG
BWF489_125  GATTATAAGG CTTTATTTGA AACTGAGGTG TATGATCGTT TAAGAGATGT ATGGTATGTT TCAAGTAATT TAAATATACC TAGAAGAAAT AATTGTGGTG

            1601                                                                                                    1700
       3D7  TTACGTCAAA TGGTAGAATT TATTGTATTG GGGGATATGA TGGCTCTTCT ATTATACCGA ATGTAGAAGC ATATGATCAT CGTATGAAAG CATGGGTAGA
BWF473_125  TTACGTCAAA TGGTAGAATT TATTGTATTG GGGGATATGA TGGCTCTTCT ATTATACCGA ATGTAGAAGC ATATGATCAT CGTATGAAAG CATGGGTAGA
52AI99_193  TTACGTCAAA TGGTAGAATT TATTGTATTG GGGGATATGA TGGCTCTTCT ATTATACCGA ATGTAGAAGC ATATGATCAT CGTATGAAAG CATGGGTAGA
BWF466_125  TTACGTCAAA TGGTAGAATT TATTGTATTG GGGGATATGA TGGCTCTTCT ATTATACCGA ATGTAGAAGC ATATGATCAT CGTATGAAAG CATGGGTAGA
BWF485_125  TTACGTCAAA TGGTAGAATT TATTGTATTG GGGGATATGA TGGCTCTTCT ATTATACCGA ATGTAGAAGC ATATGATCAT CGTATGAAAG CATGGGTAGA
BWF472_125  TTACGTCAAA TGGTAGAATT TATTGTATTG GGGGATATGA TGGCTCTTCT ATTATACCGA ATGTAGAAGC ATATGATCAT CGTATGAAAG CATGGGTAGA
BWF479_125  TTACGTCAAA TGGTAGAATT TATTGTATTG GGGGATATGA TGGCTCTTCT ATTATACCGA ATGTAGAAGC ATATGATCAT CGTATGAAAG CATGGGTAGA
BWF468_125  TTACGTCAAA TGGTAGAATT TATTGTATTG GGGGATATGA TGGCTCTTCT ATTATACCGA ATGTAGAAGC ATATGATCAT CGTATGAAAG CATGGGTAGA
52AJ00_193  TTACGTCAAA TGGTAGAATT TATTGTATTG GGGGATATGA TGGCTCTTCT ATTATACCGA ATGTAGAAGC ATATGATCAT CGTATGAAAG CATGGGTAGA
BWF465_125  TTACGTCAAA TGGTAGAATT TATTGTATTG GGGGATATGA TGGCTCTTCT ATTATACCGA ATGTAGAAGC ATATGATCAT CGTATGAAAG CATGGGTAGA
BWF477_125  TTACGTCAAA TGGTAGAATT TATTGTATTG GGGGATATGA TGGCTCTTCT ATTATACCGA ATGTAGAAGC ATATGATCAT CGTATGAAAG CATGGGTAGA
BWF471_125  TTACGTCAAA TGGTAGAATT TATTGTATTG GGGGATATGA TGGCTCTTCT ATTATACCGA ATGTAGAAGC ATATGATCAT CGTATGAAAG CATGGGTAGA
BWF489_125  TTACGTCAAA TGGTAGAATT TATTGTATTG GGGGATATGA TGGCTCTTCT ATTATACCGA ATGTAGAAGC ATATGATCAT CGTATGAAAG CATGGGTAGA

            1701                                                                                                    1800
       3D7  GGTGGCACCT TTGAATACCC CTAGATCATC AGCTATGTGT GTTGCTTTTG ATAATAAAAT TTATGTCATT GGTGGAACTA ATGGTGAGAG ATTAAATTCT
BWF473_125  GGTGGCACCT TTGAATACCC CTAGATCATC AGCTATGTGT GTTGCTTTTG ATAATAAAAT TTATGTCATT GGTGGAACTA ATGGTGAGAG ATTAAATTCT
52AI99_193  GGTGGCACCT TTGAATACCC CTAGATCATC AGCTATGTGT GTTGCTTTTG ATAATAAAAT TTATGTCATT GGTGGAACTA ATGGTGAGAG ATTAAATTCT
BWF466_125  GGTGGCACCT TTGAATACCC CTAGATCATC AGCTATGTGT GTTGCTTTTG ATAATAAAAT TTATGTCATT GGTGGAACTA ATGGTGAGAG ATTAAATTCT
BWF485_125  GGTGGCACCT TTGAATACCC CTAGATCATC AGCTATGTGT GTTGCTTTTG ATAATAAAAT TTATGTCATT GGTGGAACTA ATGGTGAGAG ATTAAATTCT
BWF472_125  GGTGGCACCT TTGAATACCC CTAGATCATC AGCTATGTGT GTTGCTTTTG ATAATAAAAT TTATGTCATT GGTGGAACTA ATGGTGAGAG ATTAAATTCT
BWF479_125  GGTGGCACCT TTGAATACCC CTAGATCATC AGCTATGTGT GTTGCTTTTG ATAATAAAAT TTATGTCATT GGTGGAACTA ATGGTGAGAG ATTAAATTCT
BWF468_125  GGTGGCACCT TTGAATACCC CTAGATCATC AGCTATGTGT GTTGCTTTTG ATAATAAAAT TTATGTCATT GGTGGAACTA ATGGTGAGAG ATTAAATTCT
52AJ00_193  GGTGGCACCT TTGAATACCC CTAGATCATC AGCTATGTGT GTTGCTTTTG ATAATAAAAT TTATGTCATT GGTGGAACTA ATGGTGAGAG ATTAAATTCT
BWF465_125  GGTGGCACCT TTGAATACCC CTAGATCATC AGCTATGTGT GTTGCTTTTG ATAATAAAAT TTATGTCATT GGTGGAACTA ATGGTGAGAG ATTAAATTCT
BWF477_125  GGTGGCACCT TTGAATACCC CTAGATCATC AGCTATGTGT GTTGCTTTTG ATAATAAAAT TTATGTCATT GGTGGAACTA ATGGTGAGAG ATTAAATTCT
BWF471_125  GGTGGCACCT TTGAATACCC CTAGATCATC AGCTATGTGT GTTGCTTTTG ATAATAAAAT TTATGTCATT GGTGGAACTA ATGGTGAGAG ATTAAATTCT
BWF489_125  GGTGGCACCT TTGAATACCC CTAGATCATC AGCTATGTGT GTTGCTTTTG ATAATAAAAT TTATGTCATT GGTGGAACTA ATGGTGAGAG ATTAAATTCT
 Consensus  GGTGGCACCT TTGAATACCC CTAGATCATC AGCTATGTGT GTTGCTTTTG ATAATAAAAT TTATGTCATT GGTGGAACTA ATGGTGAGAG ATTAAATTCT
```

```
             1801                                                                                                  1900
         3D7  ATTGAAGTAT ATGAAGAAAA AATGAATAAA TGGGAACAAT TTCCATATGC CTTATTAGAA GCTAGAAGTT CAGGAGCAGC TTTTAATTAC CTTAATCAAA
  BWF473_125  ATTGAAGTAT ATGAAGAAAA AATGAATAAA TGGGAACAAT TTCCATATGC CTTATTAGAA GCTAGAAGTT CAGGAGCAGC TTTTAATTAC CTTAATCAAA
  52AI99_193  ATTGAAGTAT ATGAAGAAAA AATGAATAAA TGGGAACAAT TTCCATATGC CTTATTAGAA GCTAGAAGTT CAGGAGCAGC TTTTAATTAC CTTAATCAAA
  BWF466_125  ATTGAAGTAT ATGAAGAAAA AATGAATAAA TGGGAACAAT TTCCATATGC CTTATTAGAA GCTAGAAGTT CAGGAGCAGC TTTTAATTAC CTTAATCAAA
  BWF485_125  ATTGAAGTAT ATGAAGAAAA AATGAATAAA TGGGAACAAT TTCCATATGC CTTATTAGAA GCTAGAAGTT CAGGAGCAGC TTTTAATTAC CTTAATCAAA
  BWF472_125  ATTGAAGTAT ATGAAGAAAA AATGAATAAA TGGGAACAAT TTCCATATGC CTTATTAGAA GCTAGAAGTT CAGGAGCAGC TTTTAATTAC CTTAATCAAA
  BWF479_125  ATTGAAGTAT ATGAAGAAAA AATGAATAAA TGGGAACAAT TTCCATATGC CTTATTAGAA GCTAGAAGTT CAGGAGCAGC TTTTAATTAC CTTAATCAAA
  BWF468_125  ATTGAAGTAT ATGAAGAAAA AATGAATAAA TGGGAACAAT TTCCATATGC CTTATTAGAA GCTAGAAGTT CAGGAGCAGC TTTTAATTAC CTTAATCAAA
  52AJ00_193  ATTGAAGTAT ATGAAGAAAA AATGAATAAA TGGGAACAAT TTCCATATGC CTTATTAGAA GCTAGAAGTT CAGGAGCAGC TTTTAATTAC CTTAATCAAA
  BWF465_125  ATTGAAGTAT ATGAAGAAAA AATGAATAAA TGGGAACAAT TTCCATATGC CTTATTAGAA GCTAGAAGTT CAGGAGCAGC TTTTAATTAC CTTAATCAAA
  BWF477_125  ATTGAAGTAT ATGAAGAAAA AATGAATAAA TGGGAACAAT TTCCATATGC CTTATTAGAA GCTAGAAGTT CAGGAGCAGC TTTTAATTAC CTTAATCAAA
  BWF471_125  ATTGAAGTAT ATGAAGAAAA AATGAATAAA TGGGAACAAT TTCCATATGC CTTATTAGAA GCTAGAAGTT CAGGAGCAGC TTTTAATTAC CTTAATCAAA
  BWF489_125  ATTGAAGTAT ATGAAGAAAA AATGAATAAA TGGGAACAAT TTCCATATGC CTTATTAGAA GCTAGAAGTT CAGGAGCAGC TTTTAATTAC CTTAATCAAA

             1901                                                                                                  2000
         3D7  TATATGTTGT TGGAGGTATT GATAATGAAC ATAACATATT AGATTCCGTT GAACAATATC AACCATTTAA TAAAAGATGG CAATTTCTAA ATGGTGTACC
  BWF473_125  TATATGTTGT TGGAGGTATT GATAATGAAC ATAACATATT AGATTCCGTT GAACAATATC AACCATTTAA TAAAAGATGG CAATTTCTAA ATGGTGTACC
  52AI99_193  TATATGTTGT TGGAGGTATT GATAATGAAC ATAACATATT AGATTCCGTT GAACAATATC AACCATTTAA TAAAAGATGG CAATTTCTAA ATGGTGTACC
  BWF466_125  TATATGTTGT TGGAGGTATT GATAATGAAC ATAACATATT AGATTCCGTT GAACAATATC AACCATTTAA TAAAAGATGG CAATTTCTAA ATGGTGTACC
  BWF485_125  TATATGTTGT TGGAGGTATT GATAATGAAC ATAACATATT AGATTCCGTT GAACAATATC AACCATTTAA TAAAAGATGG CAATTTCTAA ATGGTGTACC
  BWF472_125  TATATGTTGT TGGAGGTATT GATAATGAAC ATAACATATT AGATTCCGTT GAACAATATC AACCATTTAA TAAAAGATGG CAATTTCTAA ATGGTGTACC
  BWF479_125  TATATGTTGT TGGAGGTATT GATAATGAAC ATAACATATT AGATTCCGTT GAACAATATC AACCATTTAA TAAAAGATGG CAATTTCTAA ATGGTGTACC
  BWF468_125  TATATGTTGT TGGAGGTATT GATAATGAAC ATAACATATT AGATTCCGTT GAACAATATC AACCATTTAA TAAAAGATGG CAATTTCTAA ATGGTGTACC
  52AJ00_193  TATATGTTGT TGGAGGTATT GATAATGAAC ATAACATATT AGATTCCGTT GAACAATATC AACCATTTAA TAAAAGATGG CAATTTCTAA ATGGTGTACC
  BWF465_125  TATATGTTGT TGGAGGTATT GATAATGAAC ATAACATATT AGATTCCGTT GAACAATATC AACCATTTAA TAAAAGATGG CAATTTCTAA ATGGTGTACC
  BWF477_125  TATATGTTGT TGGAGGTATT GATAATGAAC ATAACATATT AGATTCCGTT GAACAATATC AACCATTTAA TAAAAGATGG CAATTTCTAA ATGGTGTACC
  BWF471_125  TATATGTTGT TGGAGGTATT GATAATGAAC ATAACATATT AGATTCCGTT GAACAATATC AACCATTTAA TAAAAGATGG CGATTTCTAA ATGGTGTACC
  BWF489_125  TATATGTTGT TGGAGGTATT GATAATGAAC ATAACATATT AGATTCCGTT GAACAATATC AACCATTTAA TAAAAGATGG CAATTTCTAA ATGGTGTACC
   Consensus  TATATGTTGT TGGAGGTATT GATAATGAAC ATAACATATT AGATTCCGTT GAACAATATC AACCATTTAA TAAAAGATGG CAATTTCTAA ATGGTGTACC


             1401                                                                                                  1500
         3D7  AACAATGCTG GCGTATGTGT ACACCTATGT CTACCAAAAA AGCTTATTTT GGAAGTGCTG TATTGAATAA TTTCTTATAC GTTTTTGGTG GTAATAACTA
  BWF462_125  AACAATGCTG GCGTATGTGT ACACCTATGT CTACCAAAAA AGCTTATTTT GGAAGTGCTG TATTGAATAA TTTCTTATAC GTTTTTGGTG GTAATAACTA
  BWF475_125  AACAATGCTG GCGTATGTGT ACACCTATGT CTACCAAAAA AGCTTATTTT GGAAGTGCTG TATTGAATAA TTTCTTATAC GTTTTTGGTG GTAATAACTA
  BWF487_125  AACAATGCTG GCGTATGTGT ACACCTATGT CTACCAAAAA AGCTTATTTT GGAAGTGCTG TATTGAATAA TTTCTTATAC GTTTTTGGTG GTAATAACTA
  BWF490_125  AACAATGCTG GCGTATGTGT ACACCTATGT CTACCAAAAA AGCTTATTTT GGAAGTGCTG TATTGAATAA TTTCTTATAC GTTTTTGGTG GTAATAACTA
  BWF461_125  AACAATGCTG GCGTATGTGT ACACCTATGT CTACCAAAAA AGCTTATTTT GGAAGTGCTG TATTGAATAA TTTCTTATAC GTTTTTGGTG GTAATAACTA
  BWF492_125  AACAATGCTG GCGTATGTGT ACACCTATGT CTACCAAAAA AGCTTATTTT GGAAGTGCTG TATTGAATAA TTTCTTATAC GTTTTTGGTG GTAATAACTA
  BWF483_125  AACAATGCTG GCGTATGTGT ACACCTATGT CTACCAAAAA AGCTTATTTT GGAAGTGCTG TATTGAATAA TTTCTTATAC GTTTTTGGTG GTAATAACTA
```

```
BWF482_125  AACAATGCTG GCGTATGTGT ACACCTATGT CTACCAAAAA AGCTTATTTT GGAAGTGCTG TATTGAATAA TTTCTTATAC GTTTTTGGTG GTAATAACTA
BWF476_125  AACAATGCTG GCGTATGTGT ACACCTATGT CTACCAAAAA AGCTTATTTT GGAAGTGCTG TATTGAATAA TTTCTTATAC GTTTTTGGTG GTAATAACTA
BWF480_125  AACAATGCTG GCGTATGTGT ACACCTATGT CTACCAAAAA AGCTTATTTT GGAAGTGCTG TATTGAATAA TTTCTTATAC GTTTTTGGTG GTAATAACTA
BWF481_125  AACAATGCTG GCGTATGTGT ACACCTATGT CTACCAAAAA AGCTTATTTT GGAAGTGCTG TATTGAATAA TTTCTTATAC GTTTTTGGTG GTAATAACTA
BWF460_125  AACAATGCTG GCGTATGTGT ACACCTATGT CTACCAAAAA AGCTTATTTT GGAAGTGCTG TATTGAATAA TTTCTTATAC GTTTTTGGTG GTAATAACTA
BWF464_125  AACAATGCTG GCGTATGTGT ACACCTATGT CTACCAAAAA AGCTTATTTT GGAAGTGCTG TATTGAATAA TTTCTTATAC GTTTTTGGTG GTAATAACTA
BWF491_125  AACAATGCTG GCGTATGTGT ACACCTATGT CTACCAAAAA AGCTTATTTT GGAAGTGCTG TATTGAATAA TTTCTTATAC GTTTTTGGTG GTAATAACTA
BWF470_125  AACAATGCTG GCGTATGTGT ACACCTATGT CTACCAAAAA AGCTTATTTT GGAAGTGCTG TATTGAATAA TTTCTTATAC GTTTTTGGTG GTAATAACTA
BWF488_125  AACAATGTTG GCGTATGTGT ACACCTATGT CTACCAAAAA AGCTTATTTT GGAAGTGCTG TATTGAATAA TTTCTTATAC GTTTTTGGTG GTAATAACTA
BWF484_125  AACAATGCTG GCGTATGTGT ACACCTATGT CTACCAAAAA AGCTTATTTT GGAAGTGCTG TATTGAATAA TTTCTTATAC GTTTTTGGTG GTAATAACTA
BWF469_125

            1501                                                                                                  1600
       3D7  TGATTATAAG GCTTTATTTG AAACTGAGGT GTATGATCGT TTAAGAGATG TATGGTATGT TTCAAGTAAT TTAAATATAC CTAGAAGAAA TAATTGTGGT
BWF462_125  TGATTATAAG GCTTTATTTG AAACTGAGGT GTATGATCGT TTAAGAGATG TATGGTATGT TTCAAGTAAT TTAAATATAC CTAGAAGAAA TAATTGTGGT
BWF475_125  TGATTATAAG GCTTTATTTG AAACTGAGGT GTATGATCGT TTAAGAGATG TATGGTATGT TTCAAGTAAT TTAAATATAC CTAGAAGAAA TAATTGTGGT
BWF487_125  TGATTATAAG GCTTTATTTG AAACTGAGGT GTATGATCGT TTAAGAGATG TATGGTATGT TTCAAGTAAT TTAAATATAC CTAGAAGAAA TAATTGTGGT
BWF490_125  TGATTATAAG GCTTTATTTG AAACTGAGGT GTATGATCGT TTAAGAGATG TATGGTATGT TTCAAGTAAT TTAAATATAC CTAGAAGAAA TAATTGTGGT
BWF461_125  TGATTATAAG GCTTTATTTG AAACTGAGGT GTATGATCGT TTAAGAGATG TATGGTATGT TTCAAGTAAT TTAAATATAC CTAGAAGAAA TAATTGTGGT
BWF492_125  TGATTATAAG GCTTTATTTG AAACTGAGGT GTATGATCGT TTAAGAGATG TATGGTATGT TTCAAGTAAT TTAAATATAC CTAGAAGAAA TAATTGTGGT
BWF483_125  TGATTATAAG GCTTTATTTG AAACTGAGGT GTATGATCGT TTAAGAGATG TATGGTATGT TTCAAGTAAT TTAAATATAC CTAGAAGAAA TAATTGTGGT
BWF482_125  TGATTATAAG GCTTTATTTG AAACTGAGGT GTATGATCGT TTAAGAGATG TATGGTATGT TTCAAGTAAT TTAAATATAC CTAGAAGAAA TAATTGTGGT
BWF476_125  TGATTATAAG GCTTTATTTG AAACTGAGGT GTATGATCGT TTAAGAGATG TATGGTATGT TTCAAGTAAT TTAAATATAC CTAGAAGAAA TAATTGTGGT
BWF480_125  TGATTATAAG GCTTTATTTG AAACTGAGGT GTATGATCGT TTAAGAGATG TATGGTATGT TTCAAGTAAT TTAAATATAC CTAGAAGAAA TAATTGTGGT
BWF481_125  TGATTATAAG GCTTTATTTG AAACTGAGGT GTATGATCGT TTAAGAGATG TATGGTATGT TTCAAGTAAT TTAAATATAC CTAGAAGAAA TAATTGTGGT
BWF460_125  TGATTATAAG GCTTTATTTG AAACTGAGGT GTATGATCGT TTAAGAGATG TATGGTATGT TTCAAGTAAT TTAAATATAC CTAGAAGAAA TAATTGTGGT
BWF464_125  TGATTATAAG GCTTTATTTG AAACTGAGGT GTATGATCGT TTAAGAGATG TATGGTATGT TTCAAGTAAT TTAAATATAC CTAGAAGAAA TAATTGTGGT
BWF491_125  TGATTATAAG GCTTTATTTG AAACTGAGGT GTATGATCGT TTAAGAGATG TATGGTATGT TTCAAGTAAT TTAAATATAC CTAGAAGAAA TAATTGTGGT
BWF470_125  TGATTATAAG GCTTTATTTG AAACTGAGGT GTATGATCGT TTAAGAGATG TATGGTATGT TTCAAGTAAT TTAAATATAC CTAGAAGAAA TAATTGTGGT
BWF488_125  TGATTATAAG GCTTTATTTG AAACTGAGGT GTATGATCGT TTAAGAGATG TATGGTATGT TTCAAGTAAT TTAAATATAC CTAGAAGAAA TAATTGTGGT
BWF484_125  TGATTATAAG GCTTTATTTG AAACTGAGGT GTATGATCGT TTAAGAGATG TATGGTATGT TTCAAGTAAT TTAAATATAC CTAGAAGAAA TAATTGTGGT
BWF469_125

            1601                                                                                                  1700
       3D7  GTTACGTCAA ATGGTAGAAT TTATTGTATT GGGGGATATG ATGGCTCTTC TATTATACCG AATGTAGAAG CATATGATCA TCGTATGAAA GCATGGGTAG
BWF462_125  GTTACGTCAA ATGGTAGAAT TTATTGTATT GGGGGTTATG ATGGCTCTTC TATTATACCG AATGTAGAAG CATATGATCA TCGTATGAAA GCATGGGTAG
BWF475_125  GTTACGTCAA ATGGTAGAAT TTATTGTATT GGGGGATATG ATGGCTCTTC TATTATACCG AATGTAGAAG CATATGATCA TCGTATGAAA GCATGGGTAG
BWF487_125  GTTACGTCAA ATGGTAGAAT TTATTGTATT GGGGGATATG ATGGCTCTTC TATTATACCG AATGTAGAAG CATATGATCA TCGTATGAAA GCATGGGTAG
BWF490_125  GTTACGTCAA ATGGTAGAAT TTATTGTATT GGGGGATATG ATGGCTCTTC TATTATACCG AATGTAGAAG CATATGATCA TCGTATGAAA GCATGGGTAG
BWF461_125  GTTACGTCAA ATGGTAGAAT TTATTGTATT GGGGGATATG ATGGCTCTTC TATTATACCG AATGTAGAAG CATATGATCA TCGTATGAAA GCATGGGTAG
BWF492_125  GTTACGTCAA ATGGTAGAAT TTATTGTATT GGGGGATATG ATGGCTCTTC TATTATACCG AATGTAGAAG CATATGATCA TCGTATGAAA GCATGGGTAG
```

```
BWF483_125  GTTACGTCAA ATGGTAGAAT TTATTGTATT GGGGGATATG ATGGCTCTTC TATTATACCG AATGTAGAAG CATATGATCA TCGTATGAAA GCATGGGTAG
BWF482_125  GTTACGTCAA ATGGTAGAAT TTATTGTATT GGGGGATATG ATGGCTCTTC TATTATACCG AATGTAGAAG CATATGATCA TCGTATGAAA GCATGGGTAG
BWF476_125  GTTACGTCAA ATGGTAGAAT TTATTGTATT GGGGGATATG ATGGCTCTTC TATTATACCG AATGTAGAAG CATATGATCA TCGTATGAAA GCATGGGTAG
BWF480_125  GTTACGTCAA ATGGTAGAAT TTATTGTATT GGGGGATATG ATGGCTCTTC TATTATACCG AATGTAGAAG CATATGATCA TCGTATGAAA GCATGGGTAG
BWF481_125  GTTACGTCAA ATGGTAGAAT TTATTGTATT GGGGGATATG ATGGCTCTTC TATTATACCG AATGTAGAAG CATATGATCA TCGTATGAAA GCATGGGTAG
BWF460_125  GTTACGTCAA ATGGTAGAAT TTATTGTATT GGGGGATATG ATGGCTCTTC TATTATACCG AATGTAGAAG CATATGATCA TCGTATGAAA GCATGGGTAG
BWF464_125  GTTACGTCAA ATGGTAGAAT TTATTGTATT GGGGGATATG ATGGCTCTTC TATTATACCG AATGTAGAAG CATATGATCA TCGTATGAAA GCATGGGTAG
BWF491_125  GTTACGTCAA ATGGTAGAAT TTATTGTATT GGGGGATATG ATGGCTCTTC TATTATACCG AATGTAGAAG CATATGATCA TCGTATGAAA GCATGGGTAG
BWF470_125  GTTACGTCAA ATGGTAGAAT TTATTGTATT GGGGGATATG ATGGCTCTTC TATTATACCG AATGTAGAAG CATATGATCA TCGTATGAAA GCATGGGTAG
BWF488_125  GTTACGTCAA ATGGTAGAAT TTATTGTATT GGGGGATATG ATGGCTCTTC TATTATACCG AATGTAGAAG CATATGATCA TCGTATGAAA GCATGGGTAG
BWF484_125  GTTACGTCAA ATGGTAGAAT TTATTGTATT GGGGGATATG ATGGCTCTTC TATTATACCG AATGTAGAAG CATATGATCA TCGTATGAAA GCATGGGTAG
BWF469_125                                           G ATGGCTCTTC TATTATACCG AATGTAGAAG CATATGATCA TCGTATGAAA GCATGGGTAG

            1701                                                                                                   1800
       3D7  AGGTGGCACC TTTGAATACC CCTAGATCAT CAGCTATGTG TGTTGCTTTT GATAATAAAA TTTATGTCAT TGGTGGAACT AATGGTGAGA GATTAAATTC
BWF462_125  AGGTGGCACC TTTGAATACC CCTAGATCAT CAGCTATGTG TGTTGCTTTT GATAATAAAA TTTATGTCAT TGGTGGAACT AATGGTGAGA GATTAAATTC
BWF475_125  AGGTGGCACC TTTGAATACC CCTAGATCAT CAGCTATGTG TGTTGCTTTT GATAATAAAA TTTATGTCAT TGGTGGAACT AATGGTGAGA GATTAAATTC
BWF487_125  AGGTGGCACC TTTGAATACC CCTAGATCAT CAGCTATGTG TGTTGCTTTT GATAATAAAA TTTATGTCAT TGGTGGAACT AATGGTGAGA GATTAAATTC
BWF490_125  AGGTGGCACC TTTGAATACC CCTAGATCAT CAGCTATGTG TGTTGCTTTT GATAATAAAA TTTATGTCAT TGGTGGAACT AATGGTGAGA GATTAAATTC
BWF461_125  AGGTGGCACC TTTGAATACC CCTAGATCAT CAGCTATGTG TGTTGCTTTT GATAATAAAA TTTATGTCAT TGGTGGAACT AATGGTGAGA GATTAAATTC
BWF492_125  AGGTGGCACC TTTGAATACC CCTAGATCAT CAGCTATGTG TGTTGCTTTT GATAATAAAA TTTATGTCAT TGGTGGAACT AATGGTGAGA GATTAAATTC
BWF483_125  AGGTGGCACC TTTGAATACC CCTAGATCAT CAGCTATGTG TGTTGCTTTT GATAATAAAA TTTATGTCAT TGGTGGAACT AATGGTGAGA GATTAAATTC
BWF482_125  AGGTGGCACC TTTGAATACC CCTAGATCAT CAGCTATGTG TGTTGCTTTT GATAATAAAA TTTATGTCAT TGGTGGAACT AATGGTGAGA GATTAAATTC
BWF476_125  AGGTGGCACC TTTGAATACC CCTAGATCAT CAGCTATGTG TGTTGCTTTT GATAATAAAA TTTATGTCAT TGGTGGAACT AATGGTGAGA GATTAAATTC
BWF480_125  AGGTGGCACC TTTGAATACC CCTAGATCAT CAGCTATGTG TGTTGCTTTT GATAATAAAA TTTATGTCAT TGGTGGAACT AATGGTGAGA GATTAAATTC
BWF481_125  AGGTGGCACC TTTGAATACC CCTAGATCAT CAGCTATGTG TGTTGCTTTT GATAATAAAA TTTATGTCAT TGGTGGAACT AATGGTGAGA GATTAAATTC
BWF460_125  AGGTGGCACC TTTGAATACC CCTAGATCAT CAGCTATGTG TGTTGCTTTT GATAATAAAA TTTATGTCAT TGGTGGAACT AATGGTGAGA GATTAAATTC
BWF464_125  AGGTGGCACC TTTGAATACC CCTAGATCAT CAGCTATGTG TGTTGCTTTT GATAATAAAA TTTATGTCAT TGGTGGAACT AATGGTGAGA GATTAAATTC
BWF491_125  AGGTGGCACC TTTGAATACC CCTAGATCAT CAGCTATGTG TGTTGCTTTT GATAATAAAA TTTATGTCAT TGGTGGAACT AATGGTGAGA GATTAAATTC
BWF470_125  AGGTGGCACC TTTGAATACC CCTAGATCAT CAGCTATGTG TGTTGCTTTT GATAATAAAA TTTATGTCAT TGGTGGAACT AATGGTGAGA GATTAAATTC
BWF488_125  AGGTGGCACC TTTGAATACC CCTAGATCAT CAGCTATGTG TGTTGCTTTT GATAATAAAA TTTATGTCAT TGGTGGAACT AATGGTGAGA GATTAAATTC
BWF484_125  AGGTGGCACC TTTGAATACC CCTAGATCAT CAGCTATGTG TGTTGCTTTT GATAATAAAA TTTATGTCAT TGGTGGAACT AATGGTGAGA GATTAAATTC
BWF469_125  AGGTGGCACC TTTGAATACC CCTAGATCAT CAGCTATGTG TGTTGCTTTT GATAATAAAA TTTATGTCAT TGGTGGAACT AATGGTGAGA GATTAAATTC

            1801                                                                                                   1900
       3D7  TATTGAAGTA TATGAAGAAA AAATGAATAA ATGGGAACAA TTTCCATATG CCTTATTAGA AGCTAGAAGT TCAGGAGCAG CTTTTAATTA CCTTAATCAA
BWF462_125  TATTGAAGTA TATGAAGAAA AAATGAATAA ATGGGAACAA TTTCCATATG CCTTATTAGA AGCTAGAAGT TCAGGAGCAG CTTTTAATTA CCTTAATCAA
BWF475_125  TATTGAAGTA TATGAAGAAA AAATGAATAA ATGGGAACAA TTTCCATATG CCTTATTAGA AGCTAGAAGT TCAGGAGCAG CTTTTAATTA CCTTAATCAA
BWF487_125  TATTGAAGTA TATGAAGAAA AAATGAATAA ATGGGAACAA TTTCCATATG CCTTATTAGA AGCTAGAAGT TCAGGAGCAG CATTTAATTA CCTTAATCAA
BWF490_125  TATTGAAGTA TATGAAGAAA AAATGAATAA ATGGGAACAA TTTCCATATG CCTTATTAGA AGCTAGAAGT TCAGGAGCAG CTTTTAATTA CCTTAATCAA
BWF461_125  TATTGAAGTA TATGAAGAAA AAATGAATAA ATGGGAACAA TTTCCATATG CCTTATTAGA AGCTAGAAGT TCAGGAGCAG CTTTTAATTA CCTTAATCAA
```

```
BWF492_125  TATTGAAGTA TATGAAGAAA AAATGAATAA ATGGGAACAA TTTCCATATG CCTTATTAGA AGCTAGAAGT TCAGGAGCAG CTTTTAATTA CCTTAATCAA
BWF483_125  TATTGAAGTA TATGAAGAAA AAATGAATAA ATGGGAACAA TTTCCATATG CCTTATTAGA AGCTAGAAGT TCAGGAGCAG CTTTTAATTA CCTTAATCAA
BWF482_125  TATTGAAGTA TATGAAGAAA AAATGAATAA ATGGGAACAA TTTCCATATG CCTTATTAGA AGCTAGAAGT TCAGGAGCAG CTTTTAATTA CCTTAATCAA
BWF476_125  TATTGAAGTA TATGAAGAAA AAATGAATAA ATGGGAACAA TTTCCATATG CCTTATTAGA AGCTAGAAGT TCAGGAGCAG CTTTTAATTA CCTTAATCAA
BWF480_125  TATTGAAGTA TATGAAGAAA AAATGAATAA ATGGGAACAA TTTCCATATG CCTTATTAGA AGCTAGAAGT TCAGGAGCAG CTTTTAATTA CCTTAATCAA
BWF481_125  TATTGAAGTA TATGAAGAAA AAATGAATAA ATGGGAACAA TTTCCATATG CCTTATTAGA AGCTAGAAGT TCAGGAGCAG CTTTTAATTA CCTTAATCAA
BWF460_125  TATTGAAGTA TATGAAGAAA AAATGAATAA ATGGGAACAA TTTCCATATG CCTTATTAGA AGCTAGAAGT TCAGGAGCAG CTTTTAATTA CCTTAATCAA
BWF464_125  TATTGAAGTA TATGAAGAAA AAATGAATAA ATGGGAACAA TTTCCATATG CCTTATTAGA AGCTAGAAGT TCAGGAGCAG CTTTTAATTA CCTTAATCAA
BWF491_125  TATTGAAGTA TATGAAGAAA AAATGAATAA ATGGGAACAA TTTCCATATG CCTTATTAGA AGCTAGAAGT TCAGGAGCAG CTTTTAATTA CCTTAATCAA
BWF470_125  TATTGAAGTA TATGAAGAAA AAATGAATAA ATGGGAACAA TTTCCATATG CCTTATTAGA AGCTAGAAGT TCAGGAGCAG CTTTTAATTA CCTTAATCAA
BWF488_125  TATTGAAGTA TATGAAGAAA AAATGAATAA ATGGGAACAA TTTCCATATG CCTTATTAGA AGCTAGAAGT TCAGGAGCAG CTTTTAATTA CCTTAATCAA
BWF484_125  TATTGAAGTA TATGAAGAAA AAATGAATAA ATGGGAACAA TTTCCATATG CCTTATTAGA AGCTAGAAGT TCAGGAGCAG CTTTTAATTA CCTTAATCAA
BWF469_125  TATTGAAGTA TATGAAGAAA AAATGAATAA ATGGGAACAA TTTCCATATG CCTTATTAGA AGCTAGAAGT TCAGGAGCAG CTTTTAATTA CCTTAATCAA

            1901                                                                                                  2000
       3D7  ATATATGTTG TTGGAGGTAT TGATAATGAA CATAACATAT TAGATTCCGT TGAACAATAT CAACCACTTT AATAAAAGAT GGCAATTTCT AAATGGTGTA
BWF462_125  ATATATGTTG TTGGAGGTAT TGATAATGAA CATAACATAT TAGATTCCGT TGAACAATAT CAACCACTTT AATAAAAGAT GGCAATTTCT AAATGGTGTA
BWF475_125  ATATATGTTG TTGGAGGTAT TGATAATGAA CATAACATAT TAGATTCCGT TGAACAATAT CAACCACTTT AATAAAAGAT GGCAATTTCT AAATGGTGTA
BWF487_125  ATATATGTTG TTGGAGGTAT TGATAATGAA CATAACATAT TAGATTCCGT TGAACAATAT CAACCACTTT AATAAAAGAT GGCAATTTCT AAATGGTGTA
BWF490_125  ATATATGTTG TTGGAGGTAT TGATAATGAA CATAACATAT TAGATTCCGT TGAACAATAT CAACCACTTT AATAAAAGAT GGCAATTTCT AAATGGTGTA
BWF461_125  ATATATGTTG TTGGAGGTAT TGATAATGAA CATAACATAT TAGATTCCGT TGAACAATAT CAACCACTTT AATAAAAGAT GGCAATTTCT AAATGGTGTA
BWF492_125  ATATATGTTG TTGGAGGTAT TGATAATGAA CATAACATAT TAGATTCCGT TGAACAATAT CAACCACTTT AATAAAAGAT GGCAATTTCT AAATGGTGTA
BWF483_125  ATATATGTTG TTGGAGGTAT TGATAATGAA CATAACATAT TAGATTCCGT TGAACAATAT CAACCACTTT AATAAAAGAT GGCAATTTCT AAATGGTGTA
BWF482_125  ATATATGTTG TTGGAGGTAT TGATAATGAA CATAACATAT TAGATTCCGT TGAACAATAT CAACCACTTT AATAAAAGAT GGCAATTTCT AAATGGTGTA
BWF476_125  ATATATGTTG TTGGAGGTAT TGATAATGAA CATAACATAT TAGATTCCGT TGAACAATAT CAACCACTTT AATAAAAGAT GGCAATTTCT AAATGGTGTA
BWF480_125  ATATATGTTG TTGGAGGTAT TGATAATGAA CATAACATAT TAGATTCCGT TGAACAATAT CAACCACTTT AATAAAAGAT GGCAATTTCT AAATGGTGTA
BWF481_125  ATATATGTTG TTGGAGGTAT TGATAATGAA CATAACATAT TAGATTCCGT TGAACAATAT CAACCACTTT AATAAAAGAT GGCAATTTCT AAATGGTGTA
BWF460_125  ATATATGTTG TTGGAGGTAT TGATAATGAA CATAACATAT TAGATTCCGT TGAACAATAT CAACCACTTT AATAAAAGAT GGCAATTTCT AAATGGTGTA
BWF464_125  ATATATGTTG TTGGAGGTAT TGATAATGAA CATAACATAT TAGATTCCGT TGAACAATAT CAACCACTTT AATAAAAGAT GGCAATTTCT AAATGGTGTA
BWF491_125  ATATATGTTG TTGGAGGTAT TGATAATGAA CATAACATAT TAGATTCCGT TGAACAATAT CAACCACTTT AATAAAAGAT GGCAATTTCT AAATGGTGTA
BWF470_125  ATATATGTTG TTGGAGGTAT TGATAATGAA CATAACATAT TAGATTCCGT TGAACAATAT CAACCACTTT AATAAAAGAT GGCAATTTCT AAATGGTGTA
BWF488_125  ATATATGTTG TTGGAGGTAT TGATAATGAA CATAACATAT TAGATTCCGT TGAACGATAT CAACCACTTT AATAAAAGAT GGCAATTTCT AAATGGTGTA
BWF484_125  ATATATGTTG TTGGAGGTAT TGATAATGAA CATAACATAT TAGATTCCGT TGAACAATAT CAACCACTTT AATAAAAGAT GGCAATTTCT A
BWF469_125  ATATATGTTG TTGGAGGTAT TGATAATGAA CATAACATAT TAGATTCCGT TGAACA

            1401                                                                                                  1500
       3D7  ACAATGCTGG CGTATGTGTA CACCTATGTC TACCAAAAAA GCTTATTTTG GAAGTGCTGT ATTGAATAAT TTCTTATACG TTTTTGGTGG TAATAACTAT
BWF487_125  ACAATGCTGG CGTATGTGTA CACCTATGTC TACCAAAAAA GCTTATTTTG GAAGTGCTGT ATTGAATAAT TTCTTATACG TTTTTGGTGG TAATAACTAT
52AI999_19  ACAATGCTGG CGTATGTGTA CACCTATGTC TACCAAAAAA GCTTATTTTG GAAGTGCTGT ATTGAATAAT TTCTTATACG TTTTTGGTGG TAATAACTAT
52AJ1000_1  ACAATGCTGG CGTATGTGTA CACCTATGTC TACCAAAAAA GCTTATTTTG GAAGTGCTGT ATTGAATAAT TTCTTATACG TTTTTGGTGG TAATAACTAT
52AJ1001_1  ACAATGCTGG CGTATGTGTA CACCTATGTC TACCAAAAAA GCTTATTTTG GAAGTGCTGT ATTGAATAAT TTCTTATACG TTTTTGGTGG TAATAACTAT
```

```
             1501                                                                                                          1600
        3D7  GATTATAAGG CTTTATTTGA AACTGAGGTG TATGATCGTT TAAGAGATGT ATGGTATGTT TCAAGTAATT TAAATATACC TAGAAGAAAT AATTGTGGTG
BWF487_125   GATTATAAGG CTTTATTTGA AACTGAGGTG TATGATCGTT TAAGAGATGT ATGGTATGTT TCAAGTAATT TAAATATACC TAGAAGAAAT AATTGTGGTG
52AI999_19   GATTATAAGG CTTTATTTGA AACTGAGGTG TATGATCGTT TAAGAGATGT ATGGTATGTT TCAAGTAATT TAAATATACC TAGAAGAAAT AATTGTGGTG
52AJ1000_1   GATTATAAGG CTTTATTTGA AACTGAGGTG TATGATCGTT TAAGAGATGT ATGGTATGTT TCAAGTAATT TAAATATACC TAGAAGAAAT AATTGTGGTG
52AJ1001_1   GATTATAAGG CTTTATTTGA AACTGAGGTG TATGATCGTT TAAGAGATGT ATGGTATGTT TCAAGTAATT TAAATATACC TAGAAGAAAT AATTGTGGTG

             1601                                                                                                          1700
        3D7  TTACGTCAAA TGGTAGAATT TATTGTATTG GGGGATATGA TGGCTCTTCT ATTATACCGA ATGTAGAAGC ATATGATCAT CGTATGAAAG CATGGGTAGA
BWF487_125   TTACGTCAAA TGGTAGAATT TATTGTATTG GGGGATATGA TGGCTCTTCT ATTATACCGA ATGTAGAAGC ATATGATCAT CGTATGAAAG CATGGGTAGA
52AI999_19   TTACGTCAAA TGGTAGAATT TATTGTATTG GGGGATATGA TGGCTCTTCT ATTATACCGA ATGTAGAAGC ATATGATCAT CGTATGAAAG CATGGGTAGA
52AJ1000_1   TTACGTCAAA TGGTAGAATT TATTGTATTG GGGGATATGA TGGCTCTTCT ATTATACCGA ATGTAGAAGC ATATGATCAT CGTATGAAAG CATGGGTAGA
52AJ1001_1   TTACGTCAAA TGGTAGAATT TATTGTATTG GGGGATATGA TGGCTCTTCT ATTATACCGA ATGTAGAAGC ATATGATCAT CGTATGAAAG CATGGGTAGA

             1701                                                                                                          1800
        3D7  GGTGGCACCT TTGAATACCC CTAGATCATC AGCTATGTGT GTTGCTTTTG ATAATAAAAT TTATGTCATT GGTGGAACTA ATGGTGAGAG ATTAAATTCT
BWF487_125   GGTGGCACCT TTGAATACCC CTAGATCATC AGCTATGTGT GTTGCTTTTG ATAATAAAAT TTATGTCATT GGTGGAACTA ATGGTGAGAG ATTAAATTCT
52AI999_19   GGTGGCACCT TTGAATACCC CTAGATCATC AGCTATGTGT GTTGCTTTTG ATAATAAAAT TTATGTCATT GGTGGAACTA ATGGTGAGAG ATTAAATTCT
52AJ1000_1   GGTGGCACCT TTGAATACCC CTAGATCATC AGCTATGTGT GTTGCTTTTG ATAATAAAAT TTATGTCATT GGTGGAACTA ATGGTGAGAG ATTAAATTCT
52AJ1001_1   GGTGGCACCT TTGAATACCC CTAGATCATC AGCTATGTGT GTTGCTTTTG ATAATAAAAT TTATGTCATT GGTGGAACTA ATGGTGAGAG ATTAAATTCT

             1801                                                                                                          1900
        3D7  ATTGAAGTAT ATGAAGAAAA AATGAATAAA TGGGAACAAT TTCCATATGC CTTATTAGAA GCTAGAAGTT CAGGAGCAGC TTTTAATTAC CTTAATCAAA
BWF487_125   ATTGAAGTAT ATGAAGAAAA AATGAATAAA TGGGAACAAT TTCCATATGC CTTATTAGAA GCTAGAAGTT CAGGAGCAGC ATTTAATTAC CTTAATCAAA
52AI999_19   ATTGAAGTAT ATGAAGAAAA AATGAATAAA TGGGAACAAT TTCCATATGC CTTATTAGAA GCTAGAAGTT CAGGAGCAGC TTTTAATTAC CTTAATCAAA
52AJ1000_1   ATTGAAGTAT ATGAAGAAAA AATGAATAAA TGGGAACAAT TTCCATATGC CTTATTAGAA GCTAGAAGTT CAGGAGCAGC TTTTAATTAC CTTAATCAAA
52AJ1001_1   ATTGAAGTAT ATGAAGAAAA AATGAATAAA TGGGAACAAT TTCCATATGC CTTATTAGAA GCTAGAAGTT CAGGAGCAGC TTTTAATTAC CTTAATCAAA

             1901                                                                                                          2000
        3D7  TATATGTTGT TGGAGGTATT GATAATGAAC ATAACATATT AGATTCCGTT GAACAATATC AACCATTTAA TAAAAGATGG CAATTTCTAA ATGGTGTACC
BWF487_125   TATATGTTGT TGGAGGTATT GATAATGAAC ATAACATATT AGATTCCGTT GAACAATATC AACCATTTAA TAAAAGATGG CAATTTCTAA ATGGTGTACC
52AI999_19   TATATGTTGT TGGAGGTATT GATAATGAAC ATAACATATT AGATTCCGTT GAACAATATC AACCATTTAA TAAAAGATGG CAATTTCTAA ATGGTGTACC
52AJ1000_1   TATATGTTGT TGGAGGTATT GATAATGAAC ATAACATATT AGATTCCGTT GAACAATATC AACCATTTAA TAAAAGATGG CAATTTCTAA ATGGTGTACC
52AJ1001_1   TATATGTTGT TGGAGGTATT GATAATGAAC ATAACATATT AGATTCCGTT GAACAATATC AACCATTTAA TAAAAGATGG CAATTTCTAA ATGGTGTACC
```
